# MtsslWizard: In Silico Spin-Labeling and Generation of Distance Distributions in PyMOL

**DOI:** 10.1007/s00723-012-0314-0

**Published:** 2012-02-03

**Authors:** Gregor Hagelueken, Richard Ward, James H. Naismith, Olav Schiemann

**Affiliations:** Biomedical Sciences Research Complex, The University of St. Andrews, Fife, KY16 9ST UK

## Abstract

MtsslWizard is a computer program, which operates as a plugin for the PyMOL molecular graphics system. MtsslWizard estimates distances between spin labels on proteins quickly with user-configurable options through a simple graphical interface. In default mode, the program searches for ensembles of possible MTSSL conformations that do not clash with a static model of the protein. Once conformations are assigned, distance distributions between two or more ensembles are calculated, displayed, and can be exported to other software. The program’s use is evaluated in a number of challenging test cases and its strengths and weaknesses evaluated. The benefits of the program are its accuracy and simplicity.

**Electronic supplementary material:**

The online version of this article (doi:10.1007/s00723-012-0314-0) contains supplementary material, which is available to authorized users.

## Introduction

Distance determination by pulsed electron–electron double resonance (PELDOR) [or double electron–electron resonance (DEER)] spectroscopy has become a popular tool to determine exact distances in bio-macromolecules [19, 28, 29]. Since many proteins do not contain intrinsically electron paramagnetic resonance (EPR)-active (paramagnetic) centers, PELDOR relies on the introduction of spin labels, in most cases nitroxide groups. Most commonly, site-directed spin labeling (SDSL) of cysteine residues is used to introduce spin labels on the molecular surface of a protein [2]. The distances between these labels can then be determined and, if multiple sites are labeled, distance fingerprints can be produced, even for large membrane proteins [5, 11, 13, 16]. If conditions are known that drive the protein of interest into a different conformation, another fingerprint can be prepared under these conditions and the conformational changes can be investigated [13].

**Fig. 1:**
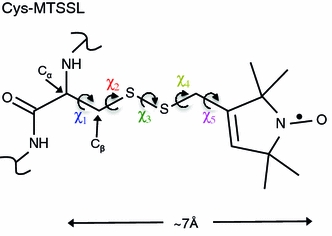
MTSSL. The position of the unpaired electron is indicated. The variable Chi-angles 1–5 are labeled

An intrinsic limitation of SDSL is due to the widely used methanethiosulfonate spin label (MTSSL [4]), which introduces five torsion angles and a >7 Å separation between the spin center and the Cα of the protein (Fig. 1). The five torsion angles lead to a very large number of possible positions for the unpaired electron. This causes uncertainty in the interpretation of experimental data in structural terms.

Different approaches have been used to deal with this problem. The uncertainty can be allowed for by adding ±14 Å to any distance restraints (e.g., [22]). This approach undermines the ability of PELDOR to identify structural changes that are well within the intrinsic error of measurement. A more sophisticated approach has been to use a priori knowledge, i.e., that the nitroxide has to be located somewhere inside a conical space around the attachment site (“tether-in-a-cone-model” [1, 14, 17, 18, 36]). This cone can be further reduced by using rotamer libraries of the free label derived from crystal structures [12, 14, 23] or molecular dynamics (MD) simulations [3, 27]. This method aims to predict in which area of the cone the label will most likely reside. The most time-intensive and rigorous method is a full-atom MD simulation of MTSSL attached to the protein (e.g., [3, 8, 9, 25, 26, 30, 31]).

We present here a computer program, mtsslWizard that allows the user to attach MTSSL labels to selected positions of a protein by pointing and clicking with a computer mouse. The program is designed as a plugin for the PyMOL (http://www.pymol.org) molecular graphics program (PyMOL plugins are often called wizards, hence our choice of name). PyMOL is widely used for the generation of figures of macromolecules in publications and its source code is available free of charge (http://sourceforge.net/projects/pymol/). Our program has a graphical user interface (GUI) that is fully integrated into PyMOL.

MtsslWizard is inspired by the “tether-in-a-cone” approach [17, 18, 36] in that the program searches for all MTSSL rotamers that do not clash with the protein structure (within a variable tolerance). The approach does not make assumptions about rotamer probability, nor does it estimate interaction energies. Despite this, in many cases, mtsslWizard predicts experimental data rather well, in a simple and rapid (seconds) manner.

## Results and Discussion

### Overview and Usage of MtsslWizard

**Fig. 2:**
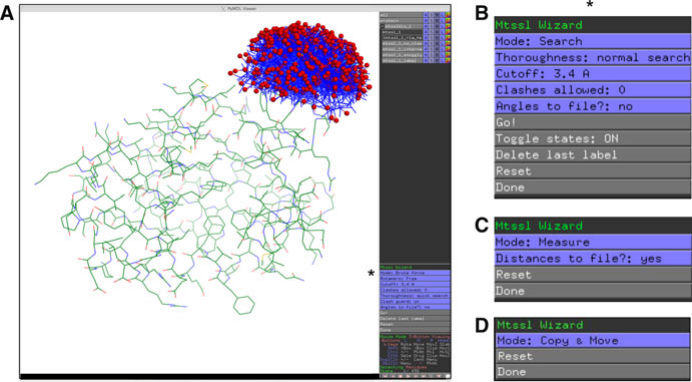
**a** The mtsslWizard GUI is integrated into PyMOL (http://www.pymol.org). The mtsslWizard menu on the *bottom right* is marked with an *asterisk*. Depending on the selection in “Mode”, the appearance of the interface changes as shown in panels **b**–**d**. **b** Menu for the “Search” mode, **c** menu for distance calculation, **d** “Copy and Move” menu allowing to transfer ensembles of conformations to symmetry related positions in multimeric proteins

MtsslWizard is launched via the PyMOL GUI and integrates seamlessly into it. Once a structure of interest has been loaded into PyMOL and mtsslWizard has been started, the residue to be labeled can be selected with a mouse click and the user can adjust the thoroughness of the search procedure, if needed (Fig. 2). In brief, the search algorithm of the program generates random values for each of the five Chi-angles and checks the corresponding conformation of MTSSL for clashes with the protein. A clash is here defined as a violation of a “vdW cutoff” between label and protein, and not as two atoms having exactly the same position in space (see below). Depending on the “thoroughness” setting, different amounts of generation/check cycles are performed for each of the five Chi-angles: 1,500 in “painstaking search”, 300 in “thorough search”, 90 in “normal search” or 10 in “quick search”. In contrast to a full-brute search, this method allows the search procedure to be shortened (if desired) without systematically missing conformations. Each generated conformation is also checked for clashes within the label itself. If internal clashes occur, the corresponding conformation is discarded.

Depending on the search parameters and the size of the protein, the calculation for one site typically takes on the order of 10–15 s on a standard laptop computer. For very large protein assemblies (>2,000 residues), calculation times on the order of 2–10 min may occur. Wherever the protein is a symmetric multimer, only one calculation per unique site is required; the distribution is easily transferred to other symmetry related positions by simply clicking in “Copy and Move” mode of the program.

Wherever the label is attached close to a hydrophobic surface pocket, conformations that place the label inside the pocket might be particularly stable. Such a fixed conformation would give a very different distance distribution than models assuming a flexible spin label. Identifying such a pocket would be key to correctly interpreting PELDOR data and avoiding “identifying” a conformational change where none occurred. As a possible solution to this problem, mtsslWizard sums the number of atoms that reside outside the vdW cutoff of a non-clashing conformation but are still within a 4.5 Å sphere around the same label conformation. We then use the number of these contacts as a surrogate for fit to the protein surface. The conformation with the highest number of such contacts is flagged as a “snuggly fit”, as are all those conformations that have at least 75% of this maximum value (see Spa15 example, below). The “snuggly fit” algorithm is only activated when the program runs with the thoroughness set to “painstaking” since otherwise the conformational space is not sampled well enough for this feature to work properly. We would like to stress that the “snuggly fit” algorithm merely flags up possibilities for the scientist to further investigate.

MtsslWizard was initially designed as a tool to assist in the selection of labeling sites for PELDOR experiments. The program gives the user an immediate idea if a planed labeling site is too occluded for the attachment of MTSSL and if the distance between two planed labeling positions will fall into the PELDOR window of 15–80 Å. Furthermore, we found that the program is very useful to interpret PELDOR data if two or more models for a protein structure are available. In this case, a comparison of the experimental distance distributions with the in silico data can often help to decide which of the available models shows the best agreement with the experimental data.

### Accounting for Conformational Changes at the Labeling Site

Almost by definition, SDSL is performed on solvent-accessible surface residues; however, the local environment of the protein structure can change due to the introduction of the spin label. A good example for this is the spin-labeled Spa15 structure which shows some significant conformational changes compared to the wild-type structure [24, 32]. It is also important to remember that the protein structure, especially with respect to surface residues is not as static as it appears in a molecular graphics program. Surface residues are often not well defined in electron density or have multiple conformations and as yet there is no simple way to capture such uncertainty. If no experimental electron density map is available, crystallographers rely in such cases. Further, crystal packing can introduce conformational artifacts of individual surface residues. Nuclear magnetic resonance (NMR)-derived structures are a very important source of data and model structural variability explicitly by producing a structural ensemble. Any approach which implicitly assumes that a single structure is a perfect model for the solution state of the protein runs the risk of errors, since clashes in the static model may not exist in the solution structure. It is for this reasons that we have allowed the user control over two parameters in mtsslWizzard: “vdW cutoff” and “allowed clashes”, which allow conformations with apparent label/protein clashes. The “vdW cutoff” determines when a contact between label and protein is counted as a clash and is by default set to “3.4 Å” which is a typical distance (carbon–carbon) between residues in the hydrophobic core of a protein structure. This value can be changed by the user between values of 2.6 Å (length of a typical hydrogen bond, ignoring the hydrogen) and 3.4 Å. The mtsslWizard employs this cutoff value for all atom types. Although vdW radii differ between, e.g., oxygen (1.52 Å) and sulfur (1.80 Å), we found that the error of the distance prediction (~3.5 Å) of mtsslWizard and, e.g., MMM dwarfs this difference by far. The “allowed clashes” parameter is by default set to “0” but can be adjusted to values between “0” and “5”. As mentioned above, a clash is here defined as a violation of the above described “vdW cutoff” and does not mean that two atoms share exactly the same position in space. A conformation that seriously protrudes into the protein surface would have far more than five such clashes. We would envisage such parameters being varied for low-resolution structures, flexible regions, for NMR structures or homology models from SWISS-PROT or if it is known that a certain site can be labeled but no conformations can be found using the default parameters. Again we stress that the scientists have to exercise their judgment with respect to what degree they deviate from the defaults, and the program specifically cautions when the parameters are varied.

### Distance Calculation

Once two or more sites of a protein have been labeled in silico, the mtsslWizard is switched into “Distance” mode and all distances between a pair of label ensembles are determined (N–N distances between nitroxide groups), displayed and written out to a file if desired (Fig. 2c). For a quick overview, a text-based histogram of all distances is calculated and displayed together with statistical information (arithmetic mean, median, the shortest, and the longest distance) and the Cβ–Cβ distance between the selected sites. The speed of the distance calculation depends on the number of conformations at the two selected sites but typically takes ~10 s.

### Comparison of MtsslWizard Predictions with Experimental Data

#### Example 1: T4 Lysozyme and H3–H4 Histone

**Fig. 3:**
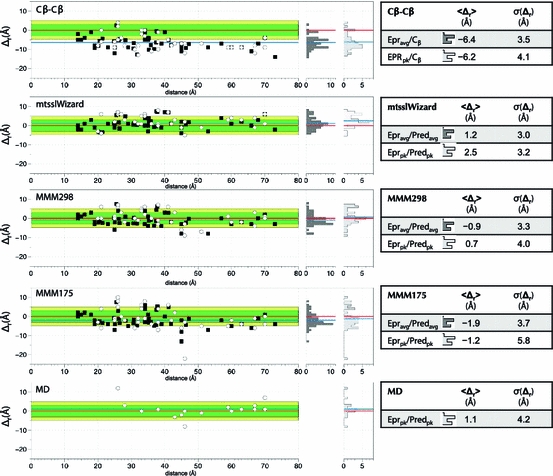
PELDOR distances derived from the T4L and Histone datasets (compare Tables 1, 2) are plotted against different in silico predictions. The *x*-axis shows the experimental distance and the *y*-axis the difference of the experimental value to the prediction (Δ_*r*_). The ideal *y* = 0 line is marked in *red*, the areas corresponding to different prediction errors are shaded with different colors (*green*: ≤3 Å, *yellow*: ≤5 Å, *white*: >5 Å). Each graph contains two plots, *white circles* show a comparison of the highest peaks of experiment and prediction (*r*(EPR)_pk_/*r*(Pred)_pk_) and *black squares* compare averages of experiment and predictions (*r*(EPR)_avg_/*r*(Pred)_avg_). The two vertical histograms (*dark gray*: EPR_avg_/Pred_avg_; *light gray*: EPR_pk_/Pred_pk_) on the *right* of each plot visualize the spread of the residuals for both plots. The average difference (<Δ_*r*_>) between prediction and experiment and the standard deviation of this value (*σ(Δ*_*r*_)) are given in the table to the *right* of each plot. The average difference (*<Δ*_*r*_ *>*) is also marked by *blue horizontal lines* in the histograms. The *blue line* in the residual plots (*left*) corresponds to the average difference for EPR_avg_/Pred_avg_. The MD values were taken from [7]

We tested mtsslWizard against a dataset of 52 published PELDOR and CW-EPR-derived distances of double spin-labeled T4 lysozyme (T4L) and (H3–H4)_2_ histone [7]. The program was run with the default settings except for the cases indicated in Tables 1, 2. In these cases the program could not find any conformations that do not clash with the protein using the default settings. Since the sites could be labeled experimentally the vdW cutoff parameter was adjusted in these cases. In the tables and wherever available, the weighted mean (<*r*>) and the most probable/peak distances *r*_pk_ are listed. The reason for the usage of the mean distance is that the value for the mean distance is more stable in DEER analysis-derived distance distributions than the peak distance or the asymmetry of the distribution, especially in the case of broad distributions [20]. The residual plots in Fig. 3 show that on average, mtsslWizard gives a significantly better (<Δ_*r*_> = 1.2 Å, σ(Δ_*r*_) = 3.0 Å) estimate of the PELDOR experimental distance than the Cβ–Cβ distance (<Δ_*r*_> = −6.4 Å, σ(Δ_*r*_) = 3.5 Å) and performs on par with the MMM program [27] (<Δ_*r*_> = −0.9 Å, σ(Δ_*r*_) = 3.3 Å). Here, <Δ_*r*_> is the mean value of the difference between in silico and experimentally derived distance and σ(Δ_*r*_) is its standard deviation. Thus, mtsslWizard is “safe” to use, it is not worse than MMM, the current gold standard on average. Bowman et al. [7] used MD simulations to predict the PELDOR distance distributions of the histone distances in our test dataset. The data in Table 2 and Fig. 3 show that on average and within error, mtsslWizard and MMM perform very similar to this more sophisticated and time-consuming approach. The correlation plot for the Cβ–Cβ distances reveals (Fig. 3a) that the usage of Cβ–Cβ distances underestimates the experimental distance by about 6.5 Å. Adding this value to the Cβ–Cβ distances leaves a standard deviation of σ(Δ_*r*_) = 3.5 Å that is not much worse than any of the simulation approaches. Thus, as a rule of thumb, 6.5 Å can be added to any Cβ–Cβ distance to get a good idea of which experimental distance is to be expected between a pair of MTSSL spin labels. However, the benefit of simulation programs is the calculation of distributions that can be compared to experimental data.

**Table 1:** EPR-derived distances of spin-labeled T4L (PDB entry 2LZM, [33]) compared to predictions from mtsslWizard and MMM in 298K and 175K mode

AA	EPR <*r*>/*r*_pk_	Cβ–Cβ	MtsslWizard <*r*>/*r*_pk_	MMM 298 K <*r*>/*r*_pk_	MMM 175 K <*r*>/*r*_pk_
061–135^d^	47.2/–	40	49/50	47/47	46/46
065–135^d^	46.3/–	37	47/47	44/44	42/41
061–086^d^	37.5/–	37	45/46	42/43	41/42
065–086^d^	37.4/–	31	37/38	36/36	33/30
080–135^d^	36.8/–	27	36/38	33/36	32/35
061–080^d^	34/–	29	34/33	31/31	31/31
065–080^d^	26.5/–	22	25/24	24/24	23/23
119–131^b^	25/–	13	24/25	19/23	18/17
123–131^b^	23/–	14	24/25	20/22	19/19
065–076^d^	21.4/–	17	18/19	19/20	18/16
116–131^b^	19/–	11	18/19	17/20	17/20
119–128^b^	19/–	9.7	18/19	16/19	15/12
140–151^b^	18/–	17	21/21	21/20	19/20
089–093^b^	16/–	12	17/17	18/16	18/18
086–119^b^	15/–	10	13/12	16/14	16/17
120–131^b^	14/–	9	12/12 (120: vdW 3.2, t)^a^	14/13	14/14
127–151^b^	14/–	12	14/14	16/17	16/18
59/159^c^	41.9/42	34	43/43	40/40	38/40
60/90^c^	37.8/38	37	45/46	44/45	43/42
60/94^c^	25.5/26	28	31/32	33/33	33/36
60/109^c^	35.2/34	31	39/40	35/36	34/33
60/154^c^	34.1/34	34	38/39 (154: t)	38/40	40/42
62/134^c^	41.1/41	36	48/48 (both: t)	44/47	46/47
64/122^c^	34.1/33	33	38/38	38/38	38/39
82/94^c^	30.7/32	24	32/33	31/31	29/31
82/132^c^	26.3/29	21	28/27	26/27	25/25
82/155^c^	35.8/38	28	38/39	35/37	33/31
83/123^c^	20.5/21	15	17/17	19/21	18/15
93/112^c^	26.1/26	21	32/33 (112:vdW3.2, t)	30/32	29/31
93/123^c^	24.8/25	19	26/27	25/26	24/24
93/154^c^	25.1/25	16	26/27 (154:t)	23/25	21/23
94/132^c^	31.7/32	19	32/33 (both: t)	29/31	27/29
108/155^c^	35.2/36	25	37/37	34/36	32/32
109/134^c^	30.6/31	21	31/33	28/31	27/25
115/155^c^	28.2/27	23	33/33	29/30	26/27
116/134^c^	20.2/20	12	17/17	18/20	17/17

Distances are given in angström. MtsslWizard was run with default parameters (“normal search”, “vdW cutoff: 3.4”, “allowed clashes: 0”) if not stated otherwise. For the EPR data, the average values <*r*> are taken from the indicated publications. If available the distance distributions were digitized and the highest peak of the distribution* r*_pk_ is also given. For the prediction programs <*r*> and *r*_pk_ are given

*t* “thorough search” was used in mtsslWizard

^a^Position 120 could only be labeled with the vdW cutoff set to 3.2 and the thoroughness set to “thorough”

^b^[1]

^c^[21]

^d^[6]

**Table 2:** PELDOR derived distances of spin-labeled H3-H4 histone [7] (PDB entry 1TZY, [34]) compared to distances predicted by mtsslWizard and MMM in 298 K and 175 K mode

AA	EPR <*r* >/*r*_pk_	Cβ–Cβ	MtsslWizard <*r* >/*r*_pk_	MMM298 K <*r* >/*r*_pk_	MMM175 K <*r*>/*r*_pk_	MD * r*_pk_
H3						
V46	60/60	51	61/64	57/58	55/58	60
R49	63/63	54	65/66	61/61	59/59	64
L65	70/70	66	76/76	72/71	71/70	77
Q76	68/70	61	71/71	68/70	67/66	71
M90	57/59	50	61/62	57/60	55/54	61
Q125	29/28	20	30/31	26/26	24/26	31
H4						
N25	73/67	59	71/71	70/70	69/66	70
T30	66/67	55	66/66	63/63	60/61	68
R45	35/33	31	35/34	36/37	36/37	33
S47	35/26	30	32/33	31/33	32/34	38
L49	45/46	34	41/41	37/37	32/24	38
V60	39/38	32	38/38	35/35	35/35	39
E63	40/46	39	47/48	43/43	40/43	46
R67	45/45	38	44/44 (p)	37/39	37/39	43
T71	45/43	36	47/46 (t, vdW3.0)	42/46	43/45	40
T82	53/51	44	50/50 (t, vdW 3.0)	45/44	46/46	50

Distances are given in angström. MtsslWizard was run with default parameters (“normal search”, “vdW cutoff: 3.4”, “allowed clashes: 0”) if not stated otherwise and the average distance that the program calculates is listed. For MMM, the highest peak in the calculated distance distribution is listed. The MD values are taken from [7]. For the EPR data, the average distance and the highest peak of the distance distribution is given. For the prediction programs the average distance is given as well as the distance that corresponds to the highest peak in the predicted distance distribution

*t* thorough search, *p* painstaking search

### Unrestricted Search versus Rotamer Libraries

**Fig. 4:**
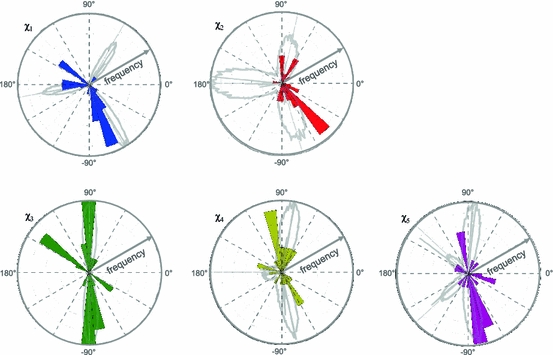
Comparison of MTSSL Chi-angles taken from available X-ray structures with a computationally produced library for the free spin label [27]. The datasets are represented by polar histograms. The experimentally found angles are color coded as follows: Chi_1_-*blue*, Chi_2_-*red*, Chi_3_-*green*, Chi_4_-*yellow*, Chi_5_-*purple*. The computed Chi-angles are overlaid as gray polar histograms and are taken from Polyhach et al. [27] Experimental Chi-angles where taken from the following PDB entries: 1zwn, 1zyt, 2cuu, 2igc, 2nth, 2ou8, 2ou9, 2q9d, 2q9e, 3g3v, 3g3w, 3g3x, 3ifx, 3m8b, 3m8d, 2w8h. In addition, the Chi-angles reported by Langen et al. [23] were used

For the comparison above, MMM was run in default mode (assuming a temperature of 175 K) and in 298 K mode. In 175 K mode we found that MMM often produces very sharp but spiky distance distributions, which can be difficult to interpret and that are usually not found in PELDOR experiments. At 298 K the MMM distributions are much more smooth and rather wide, which means that most of the MTSSL rotamers that MMM predicts for a certain site are assumed to be populated at this temperature (Supplementary Figure 1). In essence, this comes close to the mtsslWizard strategy of sampling the whole conformational space of the label at this site and calculating an average distance. Furthermore, Fig. 3 shows that for both mtsslWizard and MMM the predictions within our dataset are more reliable (smaller standard deviation) if the average of the calculated distribution is compared to the average of the experimental distribution as opposed to the peak values of experiment and prediction. This is especially obvious for MMM when run in 178 K mode where the weighting that MMM applies to the distinct rotamers becomes more apparent. This might on one hand be due to errors in the experimental distance distributions themselves (see above and [20]) or on the other hand this may indicate, that the MTSSL rotamer libraries need to be combined with a more sophisticated energy scoring function taking the protein environment, solvent and electrostatics explicitly into account. This would however come at great computational costs and only slight variations between experiment and simulation (ionic strength, pH, temperature, concentration of ethylene glycol,…) might render the outcome of such efforts futile. The influence of the protein environment on the conformation of the label becomes apparent in Fig. 4 which shows Chi-angles from PDB entries that contain MTSSL in comparison with the computationally derived rotamer library used in MMM [27]. Although the overall fit of the computational library to the experimentally observed conformations is good, there are experimental Chi-angles that are not contained in the libraries or are predicted to have a very low probability. For whatever reason—the local environment of the spin label seems to promote Chi-angles that are energetically unfavorable for the free label. We believe that such Chi-angles ought not to be excluded without good reason. Thus, mtsslWizard searches all possible Chi-angles restricted only by vdW clashes.

#### Example 2: Potassium Ion Channel KcsA

**Fig. 5:**
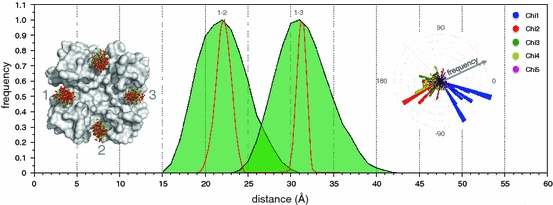
Example 2: the KcsA potassium transporter spin-labeled with mtsslWizard.* Left*: Found conformations of MTSSL are shown as* green sticks*. The O1 oxygens of MTSSL are shown as *red spheres*.* Middle*: distance histograms (*green*) produced for distances 1–2 and 1–3. The experimental PELDOR distance distributions [11] are shown as *red line*. *Right*: polar histogram plots of all Chi-angles from the MTSSL conformations shown in the *left* part of the figure

In 2009, Endeward et al. [11] analyzed spin-labeled KcsA (KcsA R64C) by PELDOR spectroscopy and derived orientation of the label from PELDOR data. We used the published results as another test for mtsslWizard and tried to predict the experimental data. The program was run using the KcsA structure by Zhou et al. [35]. Figure 5 shows the resulting spin-label ensembles. Since the label is situated in a cone-shaped cavity, the spread of the allowed conformations is rather small. The 1–2 and 1–3 distances were then calculated with mtsslWizard and a distance histogram was prepared. The maximum of the distance histogram coincides exactly with the published PELDOR results of 22 and 31 Å for 1–2 and 1–3, respectively.

We also prepared polar histograms for the Chi-angles of the mtsslWizard ensembles to compare them to values found experimentally by Endeward et al. [11]. Interestingly, Chi_1_ and Chi_2_ of the predicted ensemble are close to the experimental values of −60 and 180° indicating that in this case the protein environment drives the label into this conformation (Fig. 5).

#### Example 3: Type III Secretion Chaperone Spa15

**Fig. 6:**
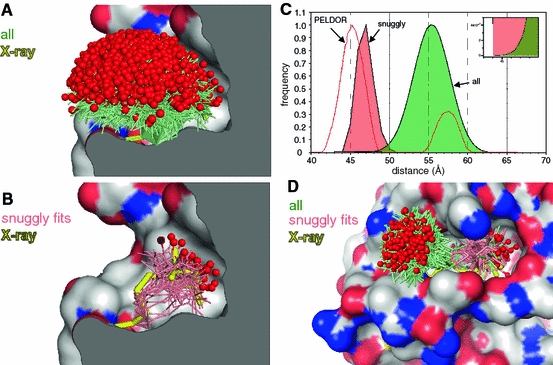
Example 3: The Spa15 chaperone. **a** All MTSSL conformations predicted by mtsslWizard are shown as *green sticks*. Note that the program was run in “painstaking” mode to activate the “snuggly fit” feature and the vdW cutoff was lowered to 2.8 Å. The MTSSL conformation found in the X-ray structure (2×ga) is colored *yellow*). The molecular surface of the protein is colored according to atom type. Carbon: *gray*, oxygen: *red*, nitrogen: *blue*. **b** Same as (**a)**, but for the subset of conformations (*salmon*) that were flagged as “snuggly fit” by mtsslWizard. The conformation observed in the crystal structure is thicker and highlighted in *yellow*. **c** The respective distance histograms after labeling both monomers of the Spa 15 dimer (for visualization purposes, both are individually scaled to values between 0 and 1). Experimental PELDOR data were digitized from [24] and is shown as *red line*. The* inset* shows a magnification of the overlapping part of the two histograms indicating that the “snuggly fitting” conformations are truly a subset of all conformations. **d** MtsslWizard results compared to the X-ray structure of spin-labeled (pos 118) T4L. The “snuggly fit” algorithm (*red sticks*) predicts the location of the spin label in a hydrophobic surface pocket, as found by crystallography. Conformations that were not flagged as “snuggly fit” by the program are colored *green*

The type III secretion effector chaperone Spa15 is an interesting test case because a crystal structure of the spin-labeled protein shows the label to reside in a confined hydrophobic surface pocket. This led to unexpected PELDOR results and the MMM program [27] was reported as failing to model the observed data, even when the crystal structure of the spin-labeled chaperone was used for the modeling efforts [24].The mtsslWizard was run with the spin-labeled Spa15 structure and a broad spread of possible conformations was found with the most frequent distance at ~56 Å. This is more than 10 Å longer than the experimentally determined PELDOR distance of 45 Å, and similar to the discrepancy that occurs with the MMM approach (Fig. 6) [24].

This problem is an ideal test case for our above mentioned “snuggly fit” algorithm and in the case of Spa15 the subsets of snuggly fitting conformations are located in the surface pocket and match quite well with the location of the label observed by crystallography (Fig. 6). The distance histogram derived from the snuggly fitting conformations peaks at 46.5 Å, close to the experimentally observed distance of 45 Å (Fig. 6). Note that the program was run in “painstaking” mode to activate the “snuggly fit” feature and the vdW cutoff was lowered to 2.8 Å. Another example that demonstrates the usability of this approach is T4L spin labeled at position 118. A crystal structure of this spin-labeled mutant was solved and shows the spin label buried in a surface cavity [15]. We “spin labeled” this position with mtsslWizard and the 15 conformations (of 265 total found conformations) that were flagged as “snugglyFits” by the program are all contained within the surface pocket close to the position found by crystallography (Fig. 6d).

#### Example 4: The *Escherichia coli* Capsule Export Channel Wza; Limitations of Current Snuggly Fit Approach

Wza is a bacterial outer membrane protein that has been crystallized with and without attached spin label [10, 16]. An MTSSL spin label attached to position Q335C of the protein was found to have two conformations by crystallography, explaining a pronounced shoulder in the PELDOR data [16].

**Fig. 7:**
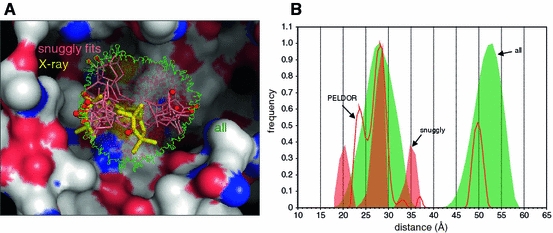
Example 4: The octameric outer membrane channel Wza. **a** All conformations found by mtsslWizard are shown in translucent and surrounded by a green outline to indicate the size of the whole distribution. Those conformations flagged as “snuggly fits” are shown as *red sticks*. The two conformations from the X-ray structure are colored *yellow*. The molecular surface of the protein is colored according to atom type. Carbon: *gray*, oxygen: *red*, nitrogen: *blue*. **b** The corresponding histograms for the 1–2 and 1–3 distances of Wza. The histograms for all conformations are filled *green*; the histograms for the “snuggly fit” conformations are colored *red*. Experimental PELDOR data were taken from [16]. Note that both the *red* and *green* histograms are individually normalized, otherwise the “snuggly fit” histogram would not be visible on the chosen *y*-axis scale

We used mtsslWizard to see if the PELDOR results including the double peak can be predicted. Distances derived from the ensemble of found conformations closely coincide with the experimental data of 28.6 and 54 Å (main peaks in [16], Fig. 7). The pronounced shoulder that was observed in the PELDOR data is however not observed in the mtsslWizard results. Figure 7 shows that both crystallographic conformations are contained in the predicted conformations, but their contribution to the histograms is overwhelmed by the other possible conformations and thus no double peak is simulated. The distance histograms of the “snuggly fitting” conformations do show multiple peaks, but deviate from the observed distances (Fig. 7). Although the simple scoring of “snuggly fit” does fail in this case, the approach does provide models for user inspection and possible further analysis.

In the Wza mutant Q335C the label is attached to the bottom of a relatively large hydrophilic crevice in the molecular surface (Fig. 6, compare molecular surface to Fig. 5). In comparison to the Spa15 and T4L examples above, this shows that our current “snuggly fit” approach may only be valid for hydrophobic pockets and less reliable for hydrophilic environments. Intuitively this seems reasonable as vdW interactions will sum but polar interactions have important geometric considerations or can be repulsive. Improvement of the “snuggly fit” algorithm can come from two sources, further increase in experimental data, which defines pockets that the label can occupy, or a more sophisticated scoring function. A more sophisticated scoring algorithm would differentiate between repulsive and attractive forces and treat polar atoms differently. The challenge for future development is to balance an increasingly sophisticated treatment of label–protein interactions against simplicity and speed.

### Interpreting MtsslWizard Results

The T4L and histone examples show that the mean distance calculated by mtsslWizard predicts the experimental values quite well and we found that in many cases the shape of the distance histogram derived from mtsslWizard is also a reasonable match to the PELDOR derived distance distribution (Supplementary Figure 1). Since mtsslWizard is based on the “tether-in-a-cone” approach [1, 14, 17, 18] the logical implication is that indeed in many cases the spin label does sample a range of conformations.

Wherever the spin label is not disordered but adopts a fixed or restricted set of conformations due to interaction with the protein, only a fraction of the possible conformations that mtsslWizard finds will match the observed data. Additional parameters such as the surface complementarity score (“snuggly fit”) have been introduced to improve the predictions (example 3: Spa15). We also found that it is often helpful to interpret mtsslWizard results in the light of room temperature CW spectra because the latter contain experimental information about the flexibility of the spin label and would thus hint to whether a label is indeed trapped in a pocket or pointing into the solvent.

## Conclusion

MtsslWizard is a simple to use and freely available program that allows users to generate plausible for MTSSL labels attached to a protein, to measure distances between labels and indeed to vary parameters to allow superficially implausible models. We have argued above that there are sound reasons (e.g. errors or uncertainties in experimental structures) not to assume each side chain of an amino acid is positioned correctly (especially on the protein surface).

As we would predict our approach produces similar results to MMM in most cases, any benefit in such cases would derive only from it being a PyMOL plugin, rather than MATLAB. Importantly, the integration of the program into a freely available, sophisticated and powerful molecular graphics program like PyMOL makes it easy for users to critically evaluate mtsslWizard results. We believe the advantage of our program occurs when PELDOR data does not match the simple model. Incorrectly interpreting disagreement between PELDOR data and a distance model in functional terms must be avoided. Determining crystal structures to resolve such anomalies is definitive but may not be possible in each case. Our approach allows the scientist to explore whether the measured distances are plausible from a range of models. As we show the program does a very good job for a number of test cases where there are unusual positions of the spin label, we stress the program is intended to provide a basis for further analysis and experimentation. By eliminating alternative explanations for discrepancy between model and data, scientists will be able to confidently identify conformational changes.

The source code of mtsslWizard is freely available at htp://www.pymolwiki.org/index.php/mtsslWizard.

## Electronic supplementary material

Below is the link to the electronic supplementary material.

Supplementary Figure 1 Superposition of distance predictions by mtsslWizard (*green*) and MMM in 175 K (*blue*, *dashed*) and 298 K (*blue*) mode. If available experimental distributions were overlaid in red. The T4L distributions were taken from [21]. The histone data is taken from [7]. (PDF 1.25 mb)

Supplementary Figure 2 (PDF 380 kb)

## Note added in proof

During the analysis of the T4L and histone datasets with mtsslWizard the vdW cutoff was in 4 cases lowered to a minimum of 3.0 Å (see Table 1 and 2). This prompted us to re-analyse the whole dataset with this lower cutoff and we found that the prediction results are the same within statistical error (see supplementary figure 2). Thus, in cases where large datasets have to be analysed it is safe to use a cutoff of 3.0 Å to increase the consistency of the analysis.
