# Supplementary material for: MtsslWizard: In Silico Spin-Labeling and Generation of Distance Distributions in PyMOL
Source: Appl Magn Reson. 2012 Feb 3;42(3):377–91. doi: 10.1007/s00723-012-0314-0 (PMC3296949; doi:10.1007/s00723-012-0314-0)
Supplement: Supplementary file 1 — Superposition of distance predictions by mtsslWizard (green) and MMM in 175 K (blue, dashed) and 298 K (blue) mode. If available experimental distributions were overlaid in red. The T4L distributions were taken from [21]. The histone data is taken from [7]. (PDF 1.25 mb) [file 723_2012_314_MOESM1_ESM.pdf]

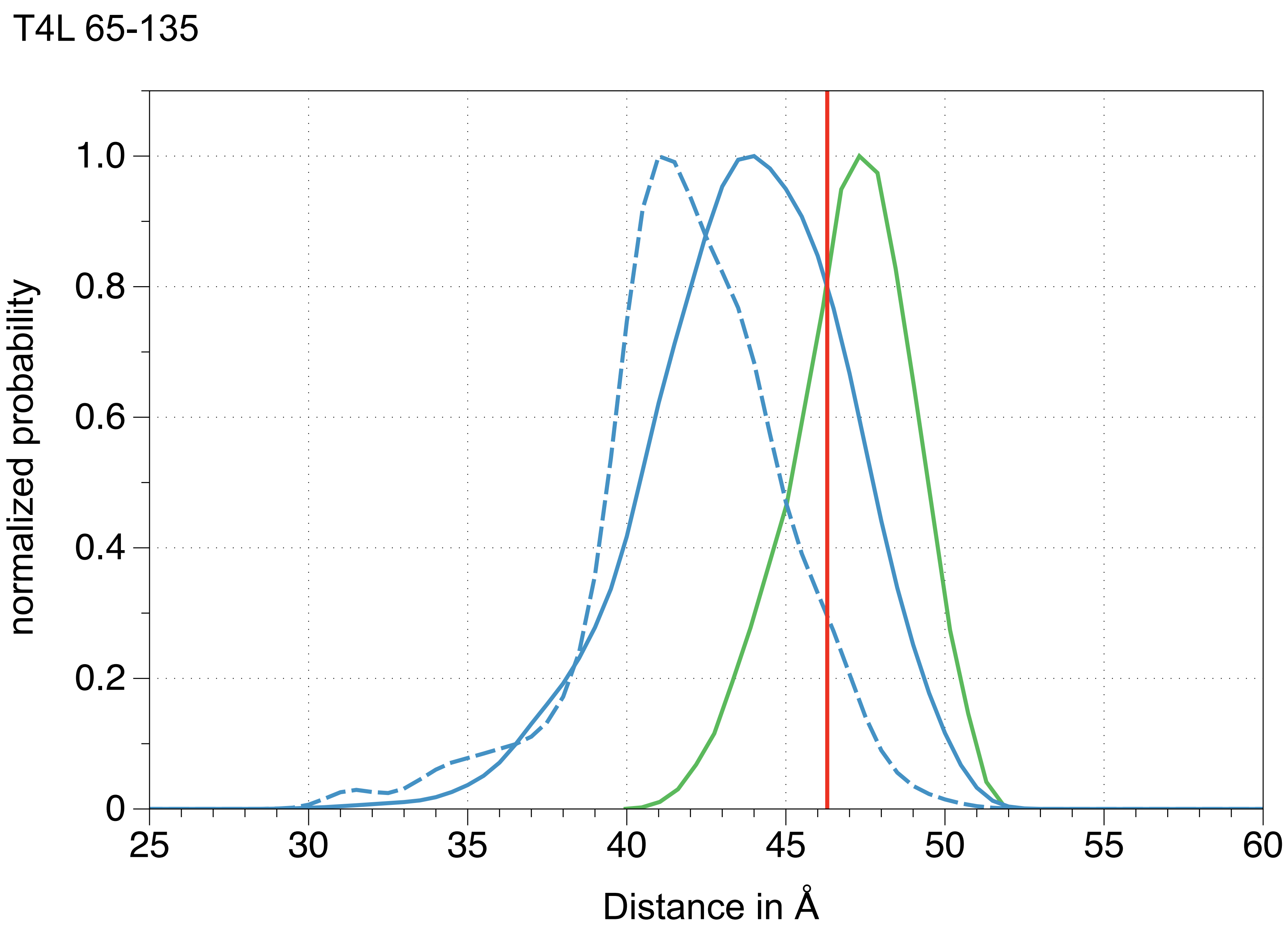

T4L 65-135
normalized probability
0
0.2
0.4
0.6
0.8
1.0
25
30
35
40
45
50
55
60
Distance in Å

T4L 61-135

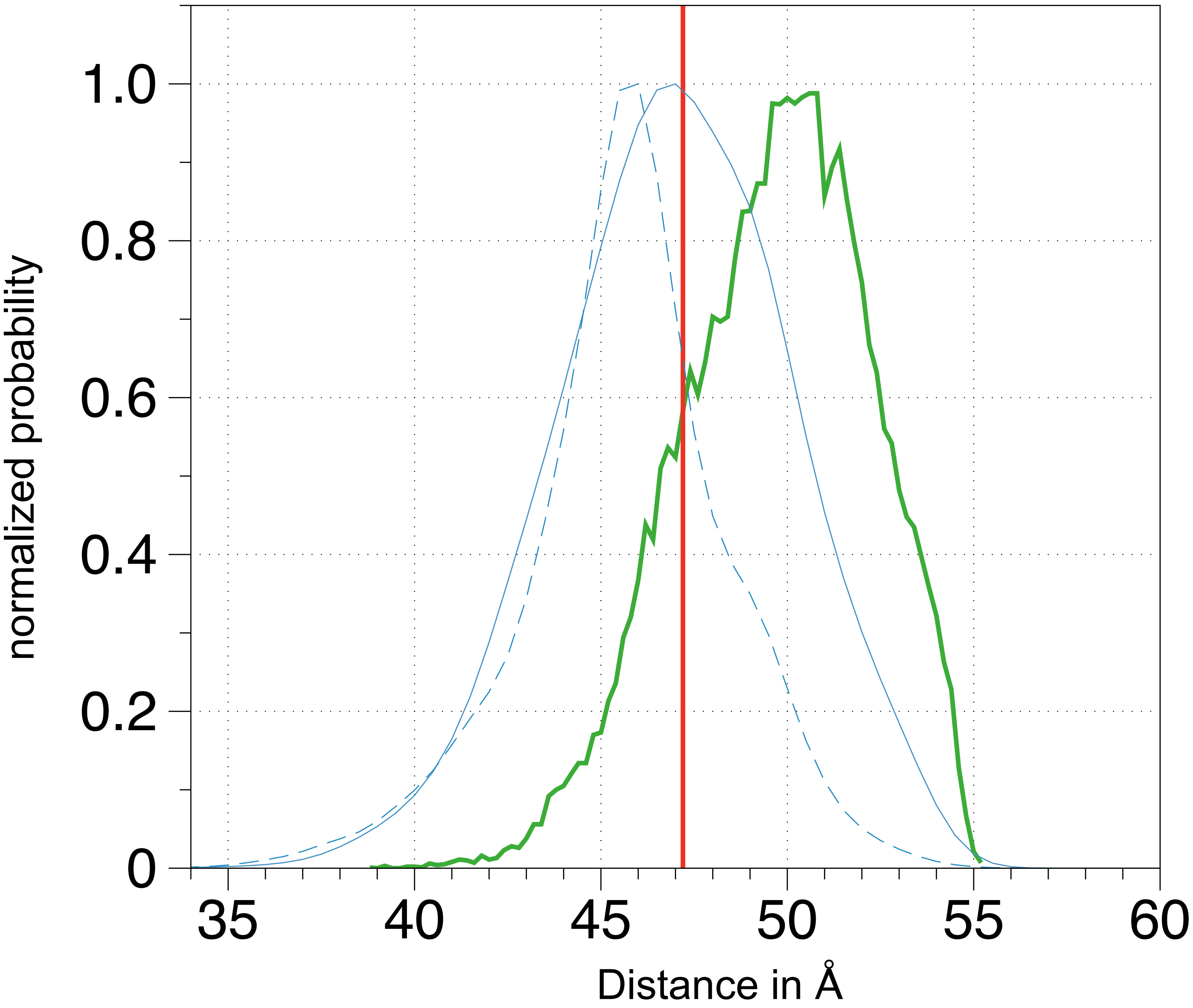

normalized probability
1.0
0.8
0.6
0.4
0.2
0
35
40
45
50
55
60
Distance in Å

T4L 59-159

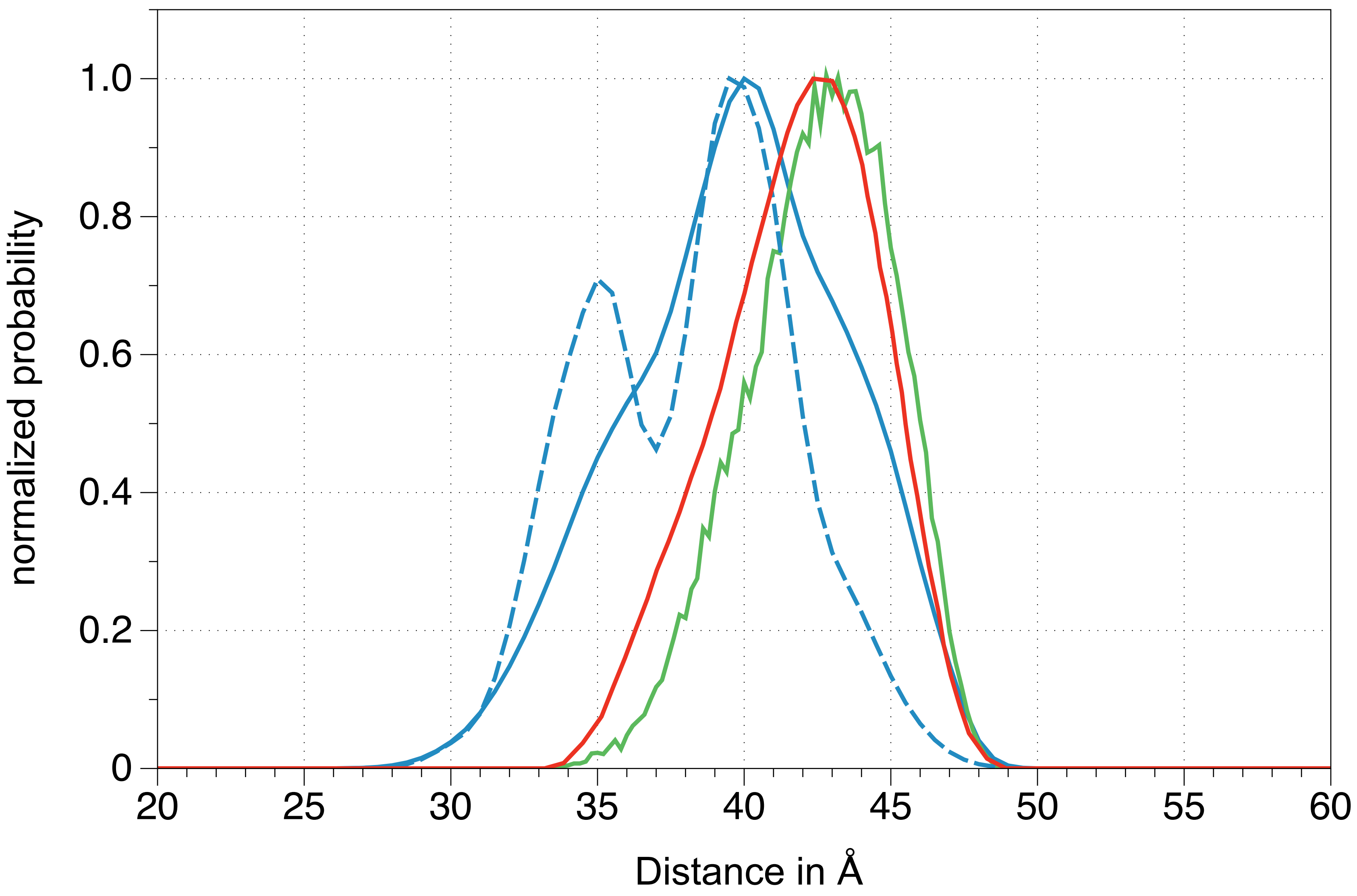

normalized probability
1.0
0.8
0.6
0.4
0.2
0
20
25
30
35
40
45
50
55
60
Distance in Å

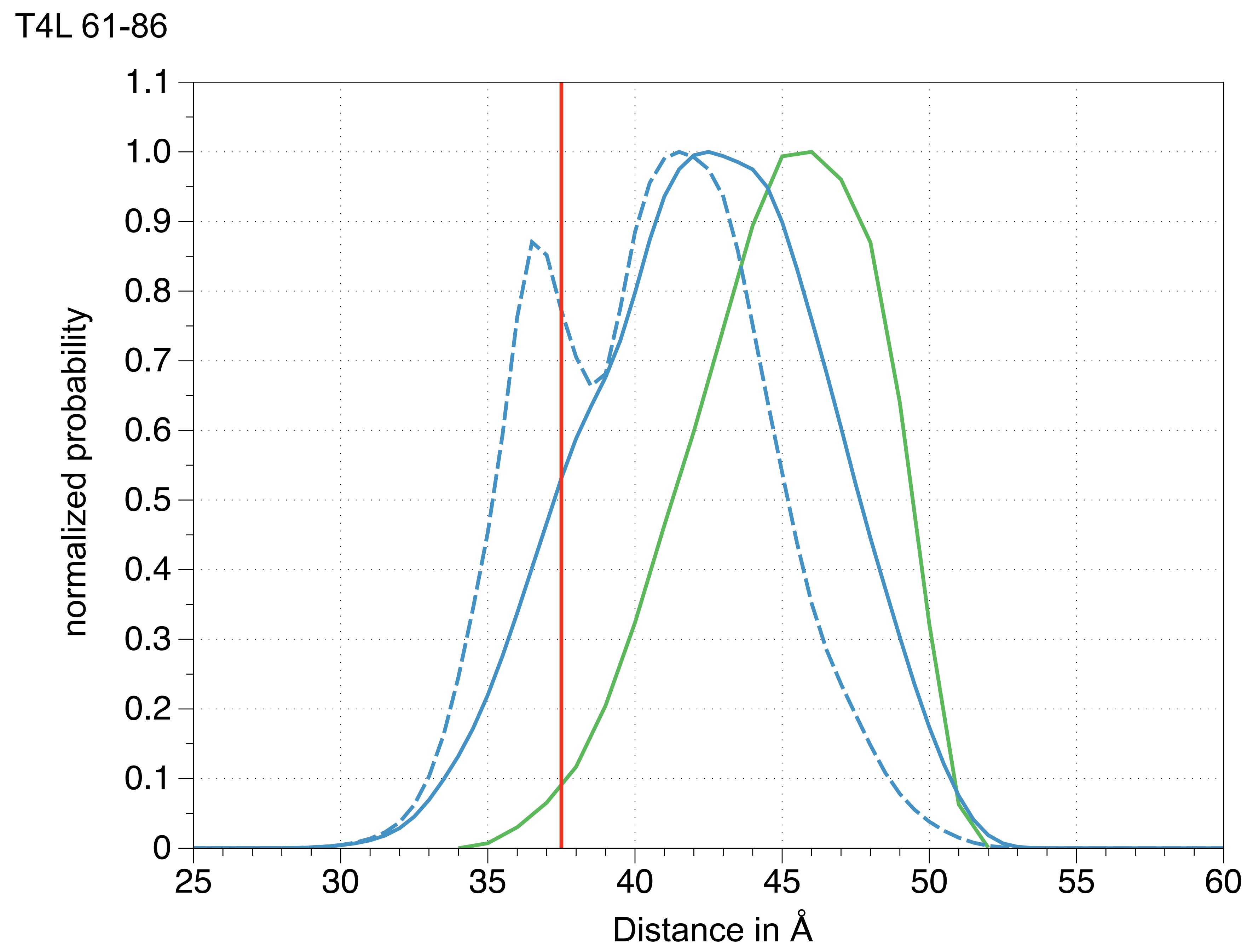
T4L 61-86
normalized probability
0
0.1
0.2
0.3
0.4
0.5
0.6
0.7
0.8
0.9
1.0
1.1
25
30
35
40
45
50
55
60
Distance in Å

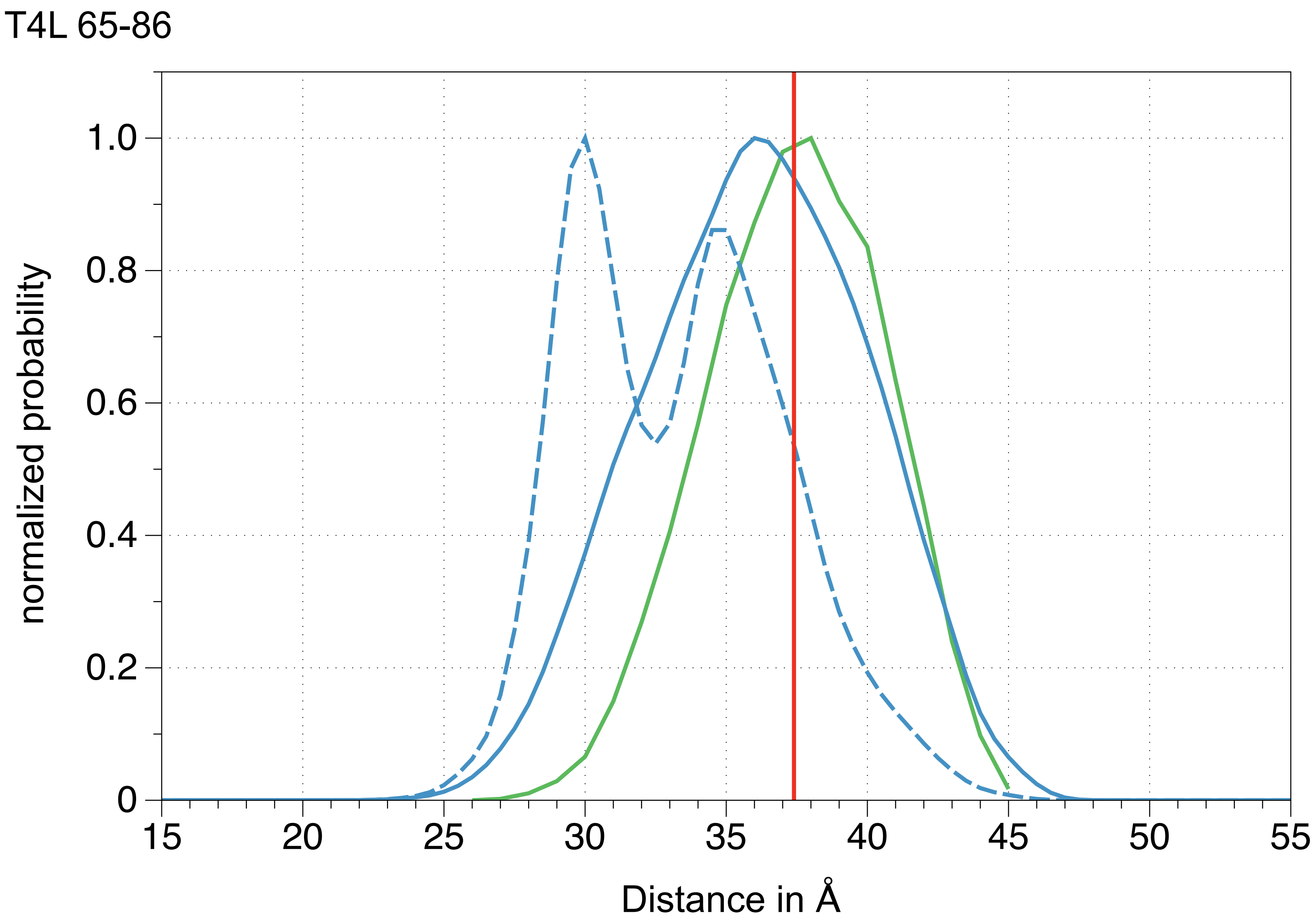
T4L 65-86
normalized probability
0
0.2
0.4
0.6
0.8
1.0
15
20
25
30
35
40
45
50
55
Distance in Å

T4L 80-135

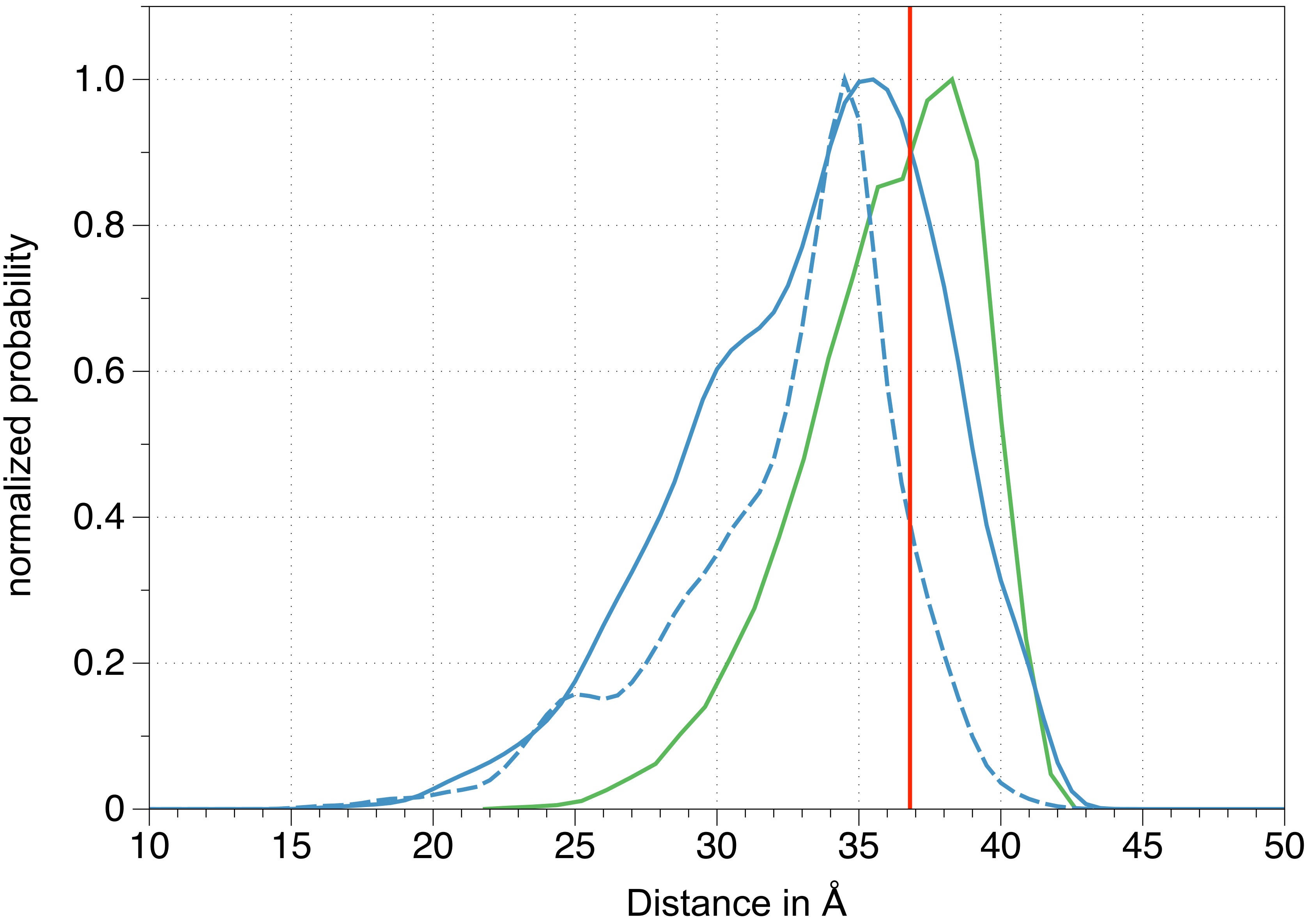

1.0
0.8
0.6
0.4
0.2
0
normalized probability
10
15
20
25
30
35
40
45
50
Distance in Å

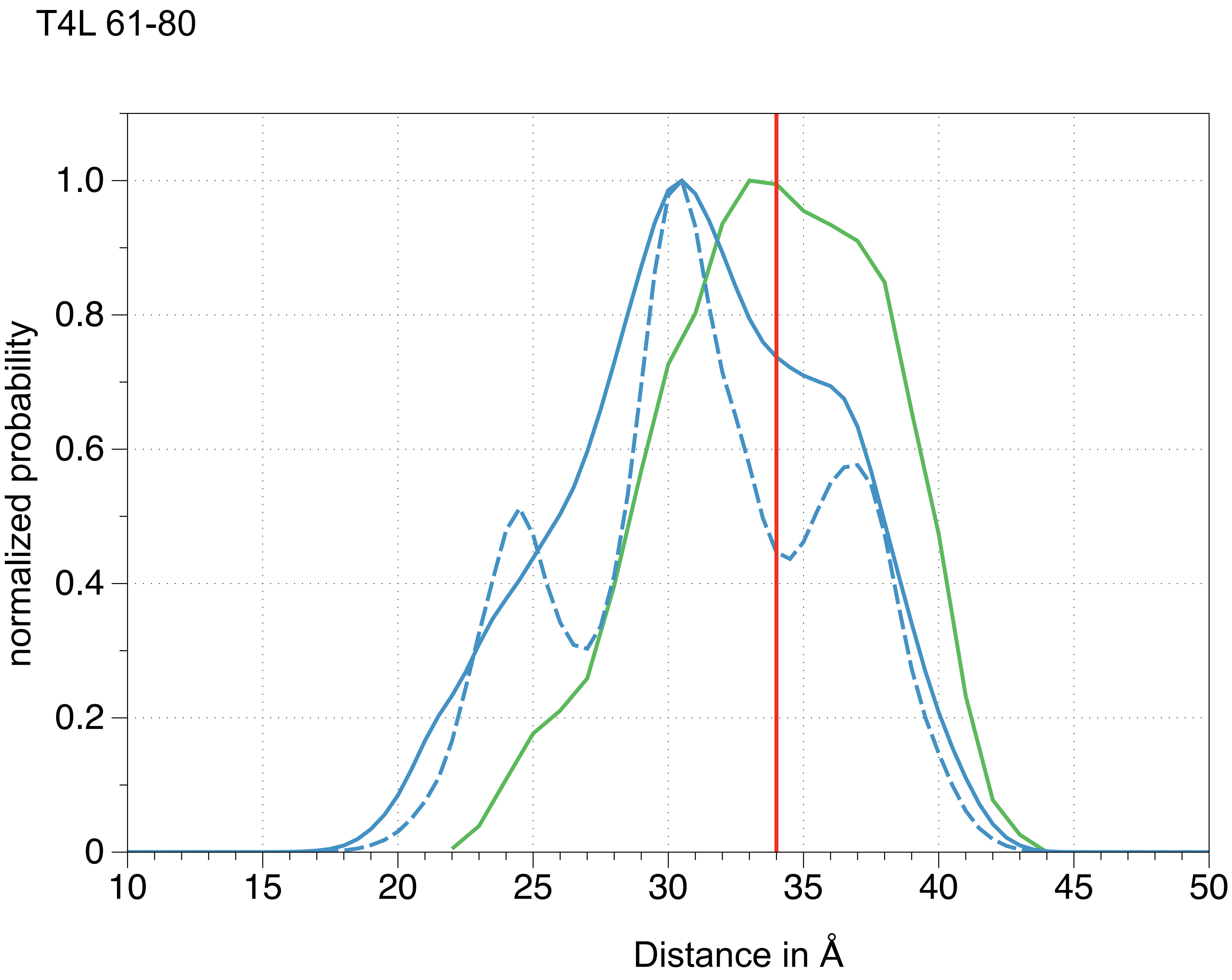

T4L 61-80
normalized probability
1.0
0.8
0.6
0.4
0.2
0
10
15
20
25
30
35
40
45
50
Distance in Å

T4L 65-80

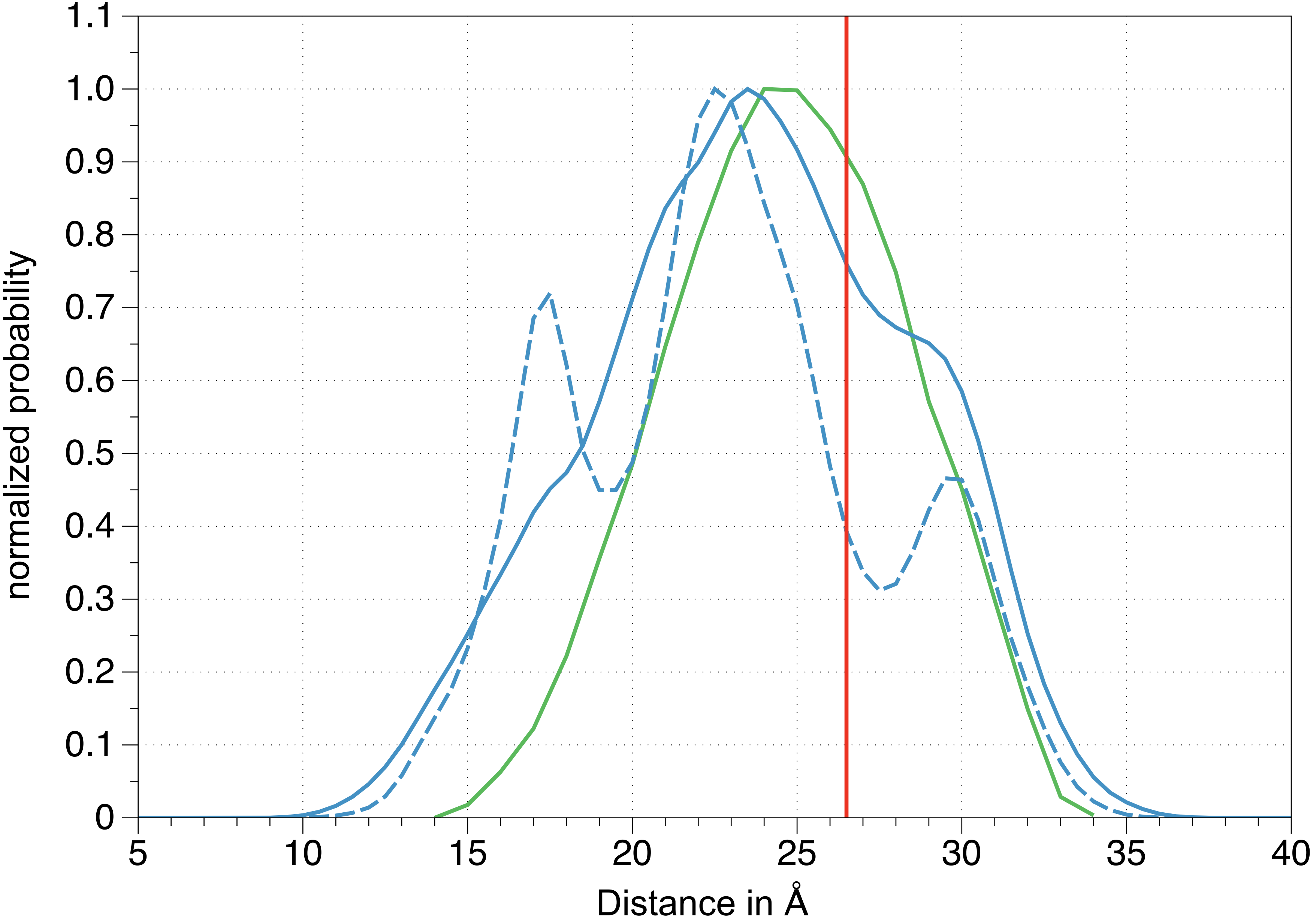
1.1
1.0
0.9
0.8
0.7
0.6
0.5
0.4
0.3
0.2
0.1
0
normalized probability
5
10
15
20
25
30
35
40
Distance in Å

T4L 119-131

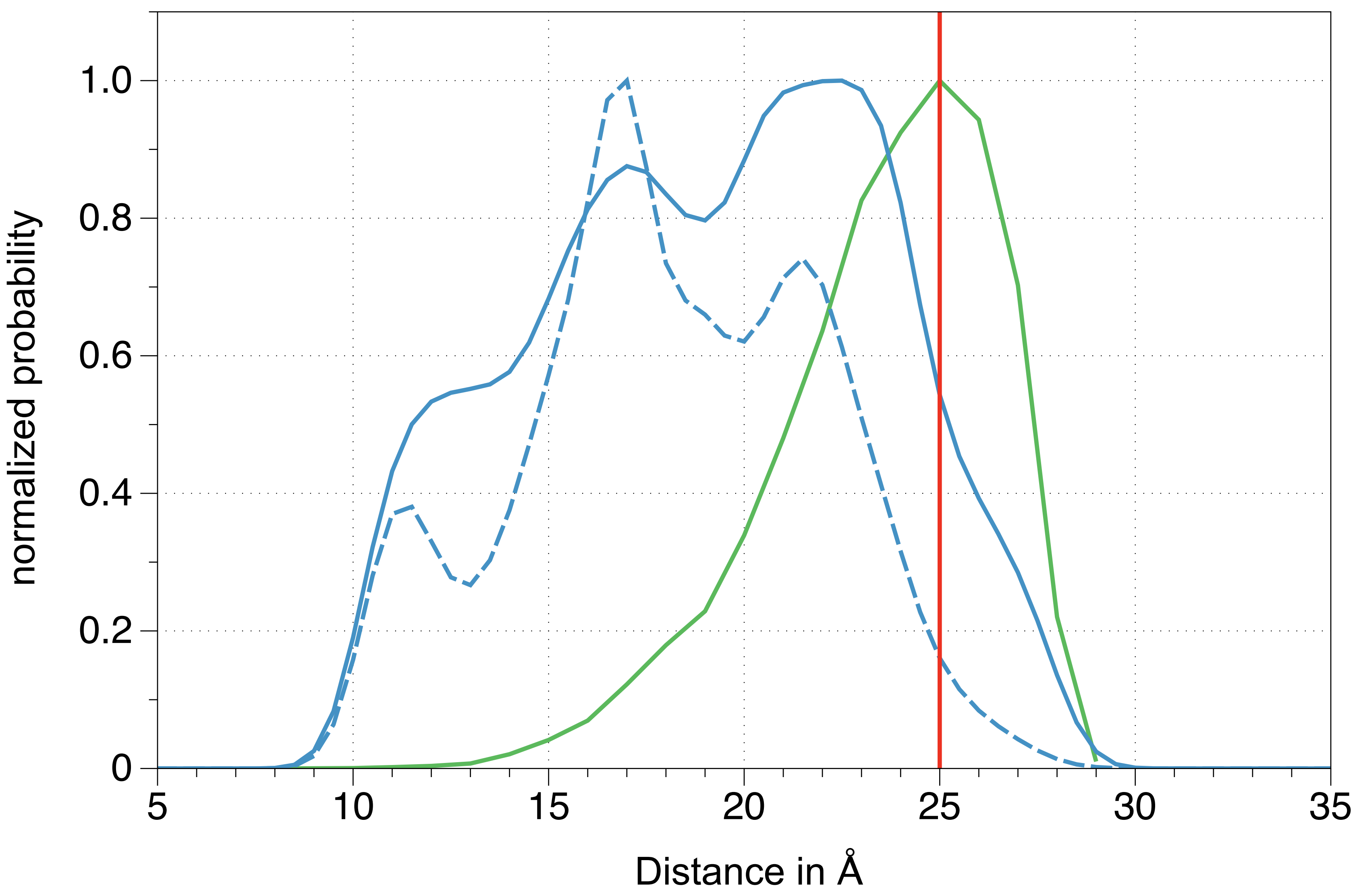

normalized probability
1.0
0.8
0.6
0.4
0.2
0
5
10
15
20
25
30
35
Distance in Å

T4L 123-131

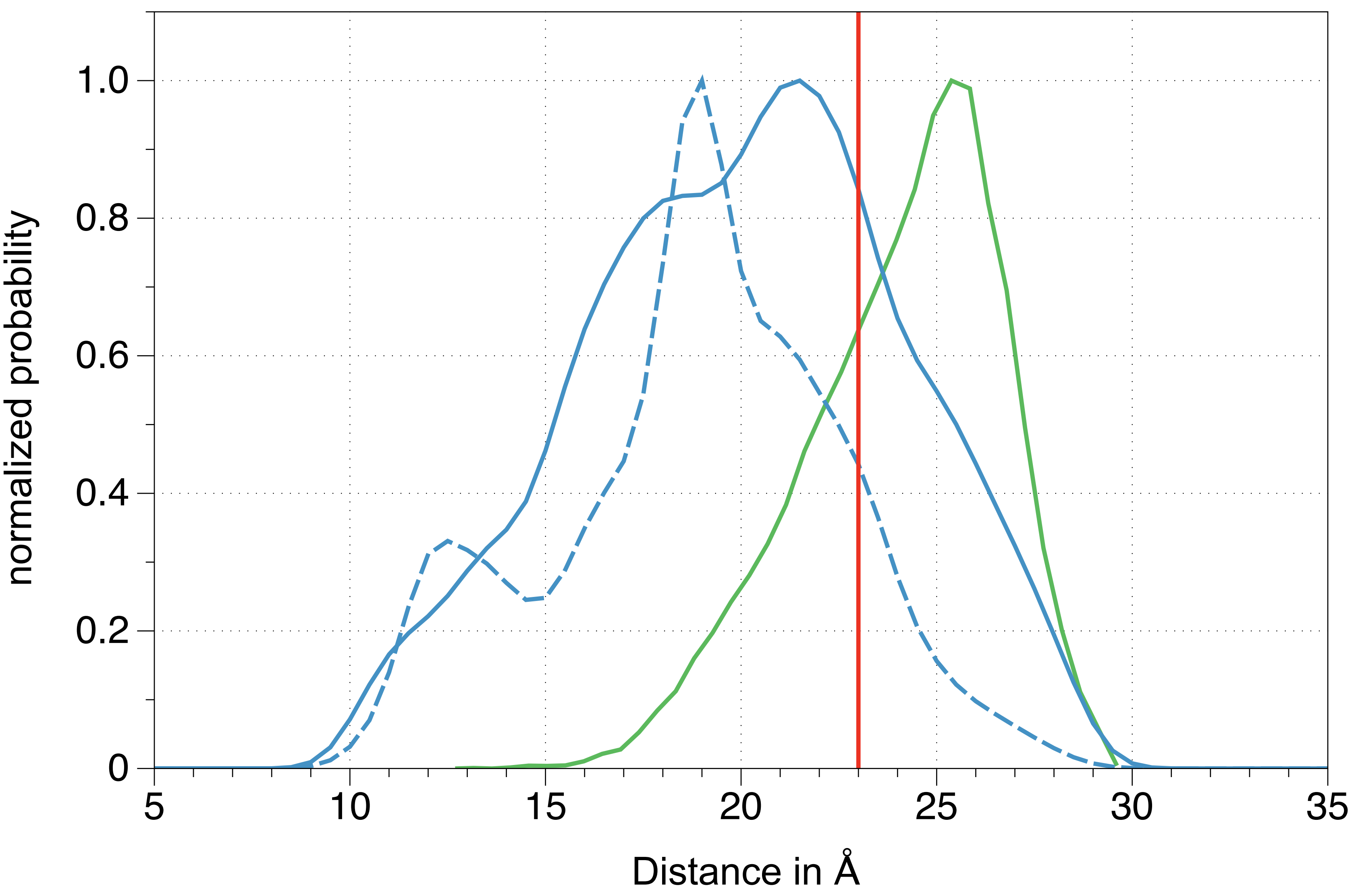

normalized probability
0
0.2
0.4
0.6
0.8
1.0
5
10
15
20
25
30
35
Distance in Å

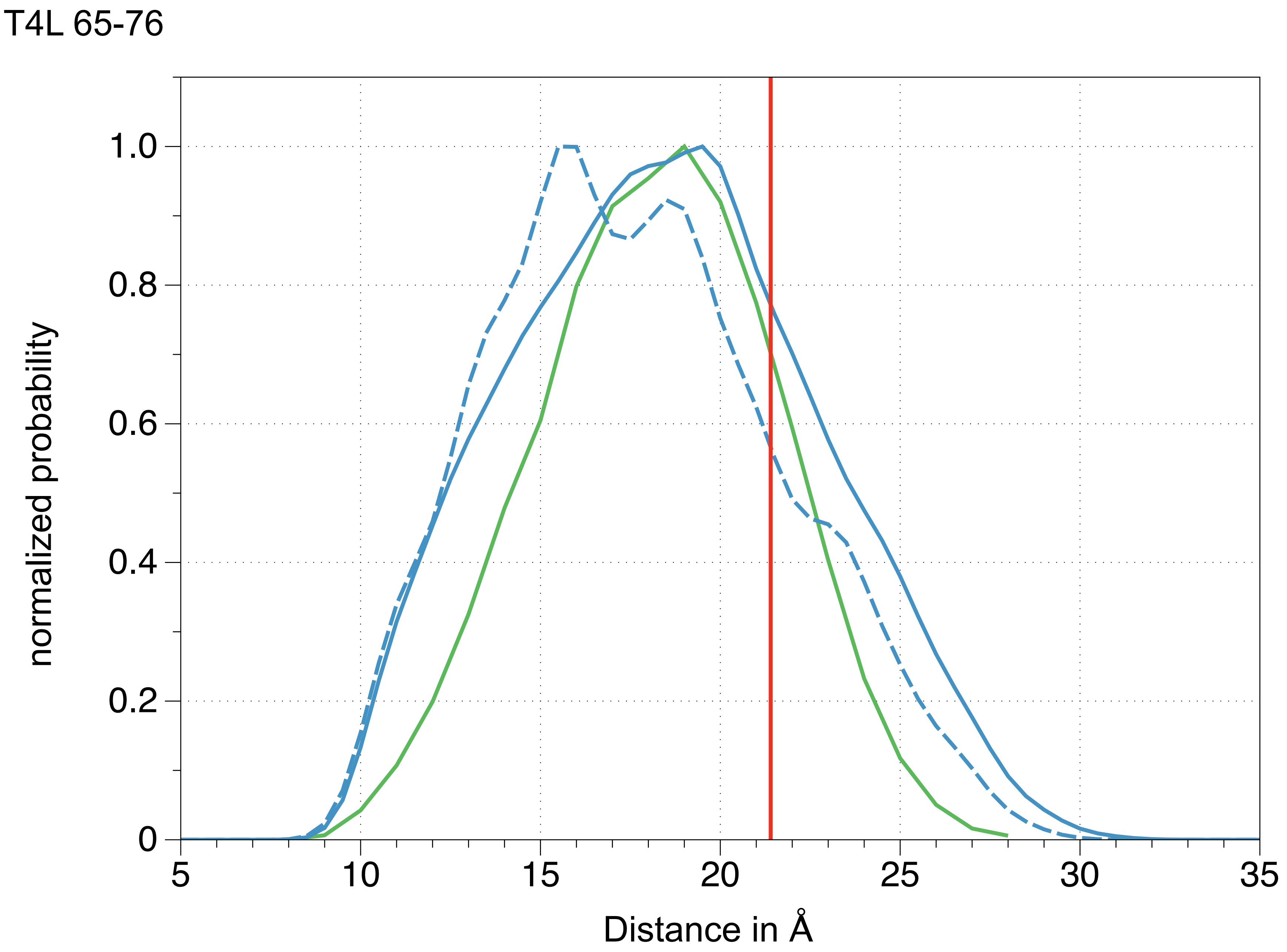
T4L 65-76
normalized probability
0
0.2
0.4
0.6
0.8
1.0
5
10
15
20
25
30
35
Distance in Å

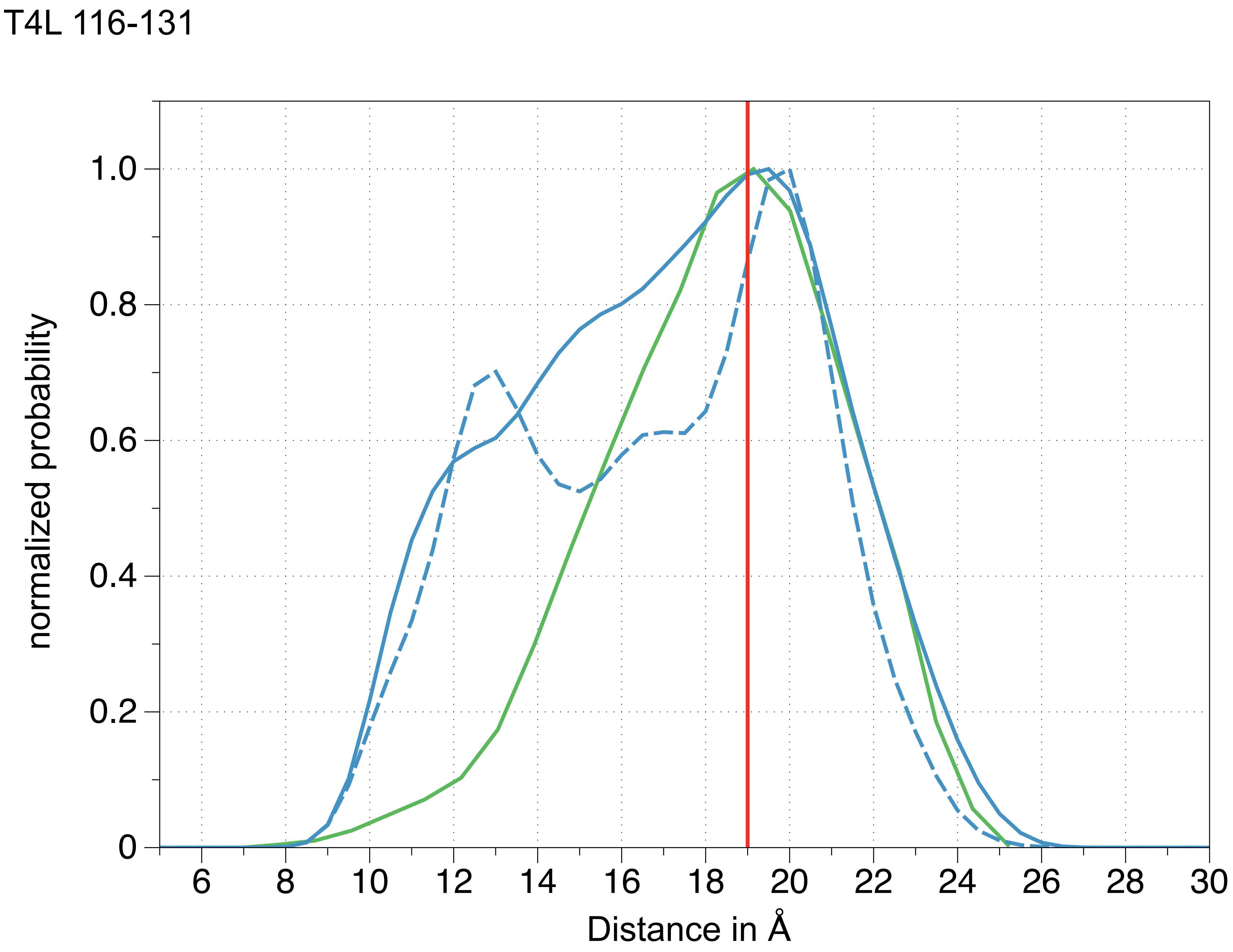

T4L 116-131
normalized probability
1.0
0.8
0.6
0.4
0.2
0
6
8
10
12
14
16
18
20
22
24
26
28
30
Distance in Å

T4L 119-128

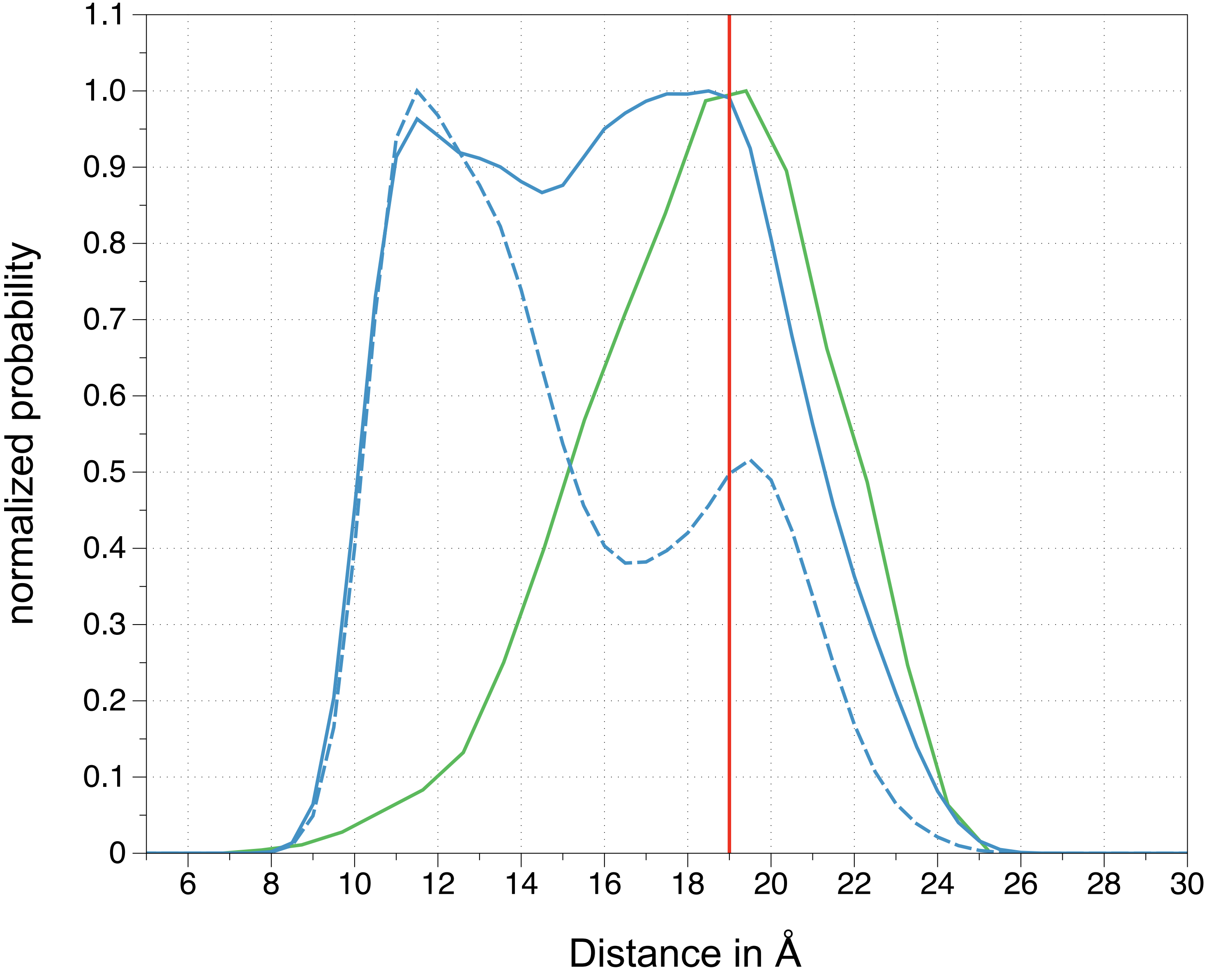
normalized probability
Distance in Å
0
0.1
0.2
0.3
0.4
0.5
0.6
0.7
0.8
0.9
1.0
1.1
6
8
10
12
14
16
18
20
22
24
26
28
30

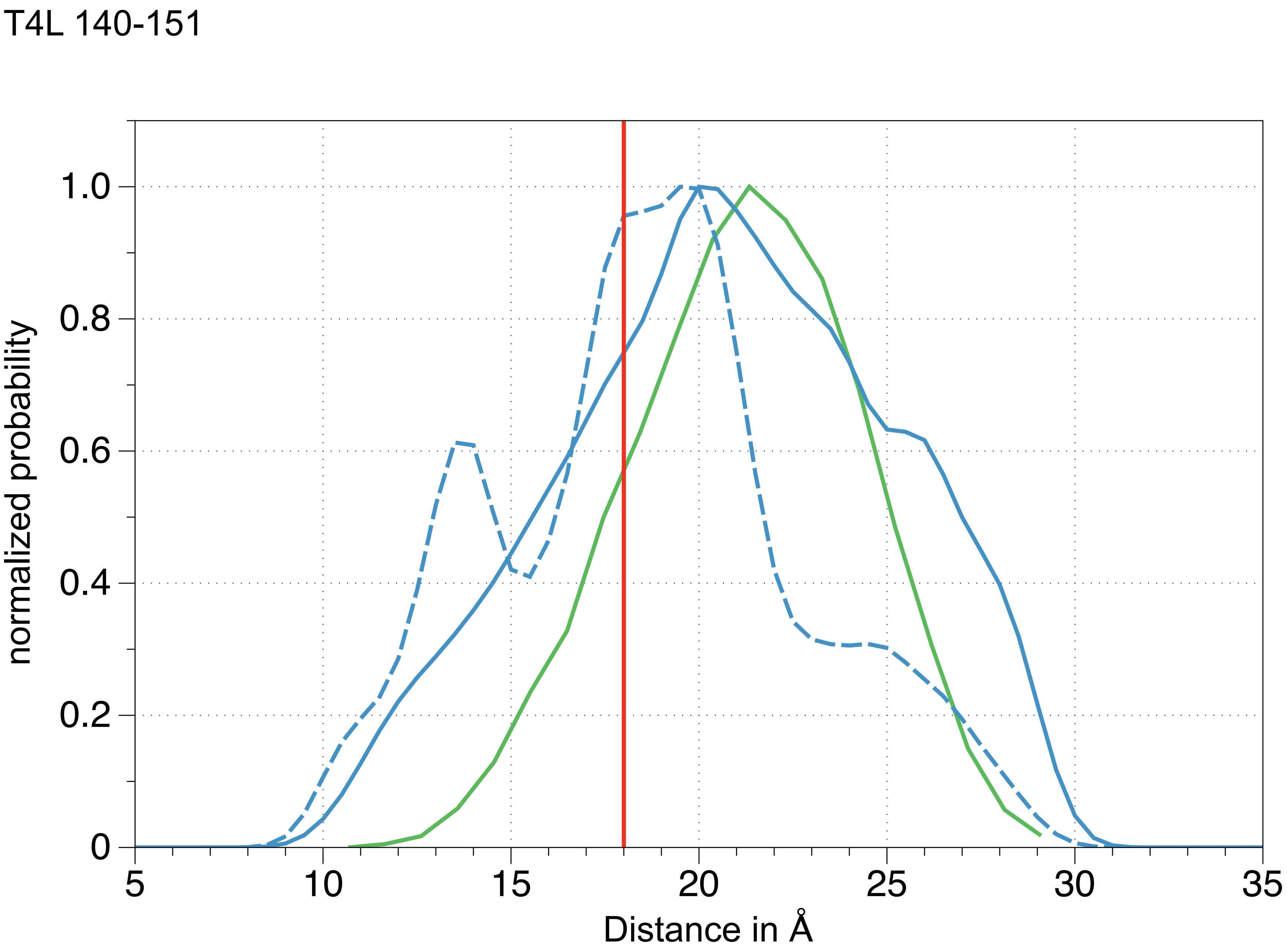
T4L 140-151
normalized probability
1.0
0.8
0.6
0.4
0.2
0
5
10
15
20
25
30
35
Distance in Å

T4L 89-93

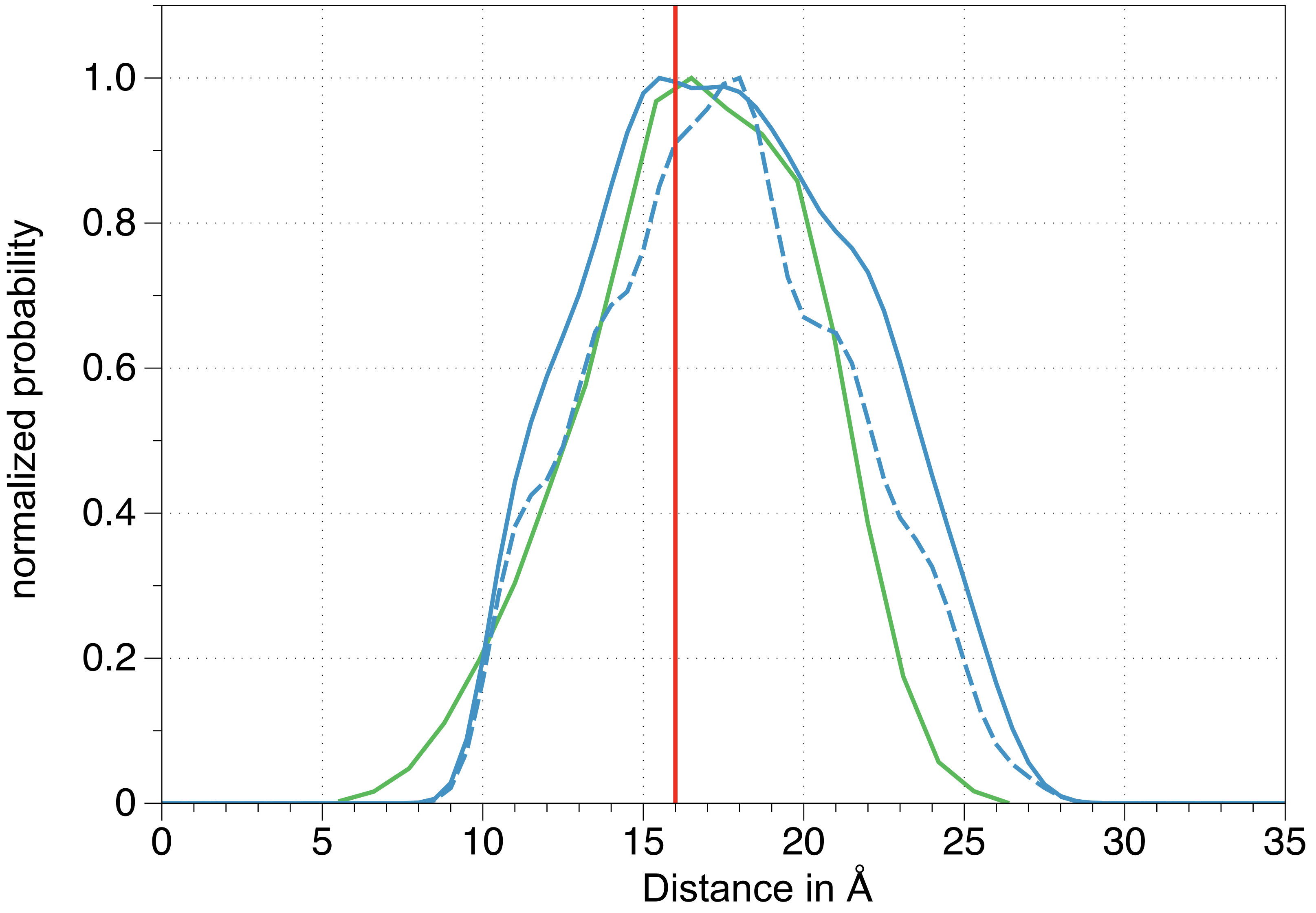

1.0
0.8
0.6
0.4
0.2
0
normalized probability
0
5
10
15
20
25
30
35
Distance in Å

T4L 86-119

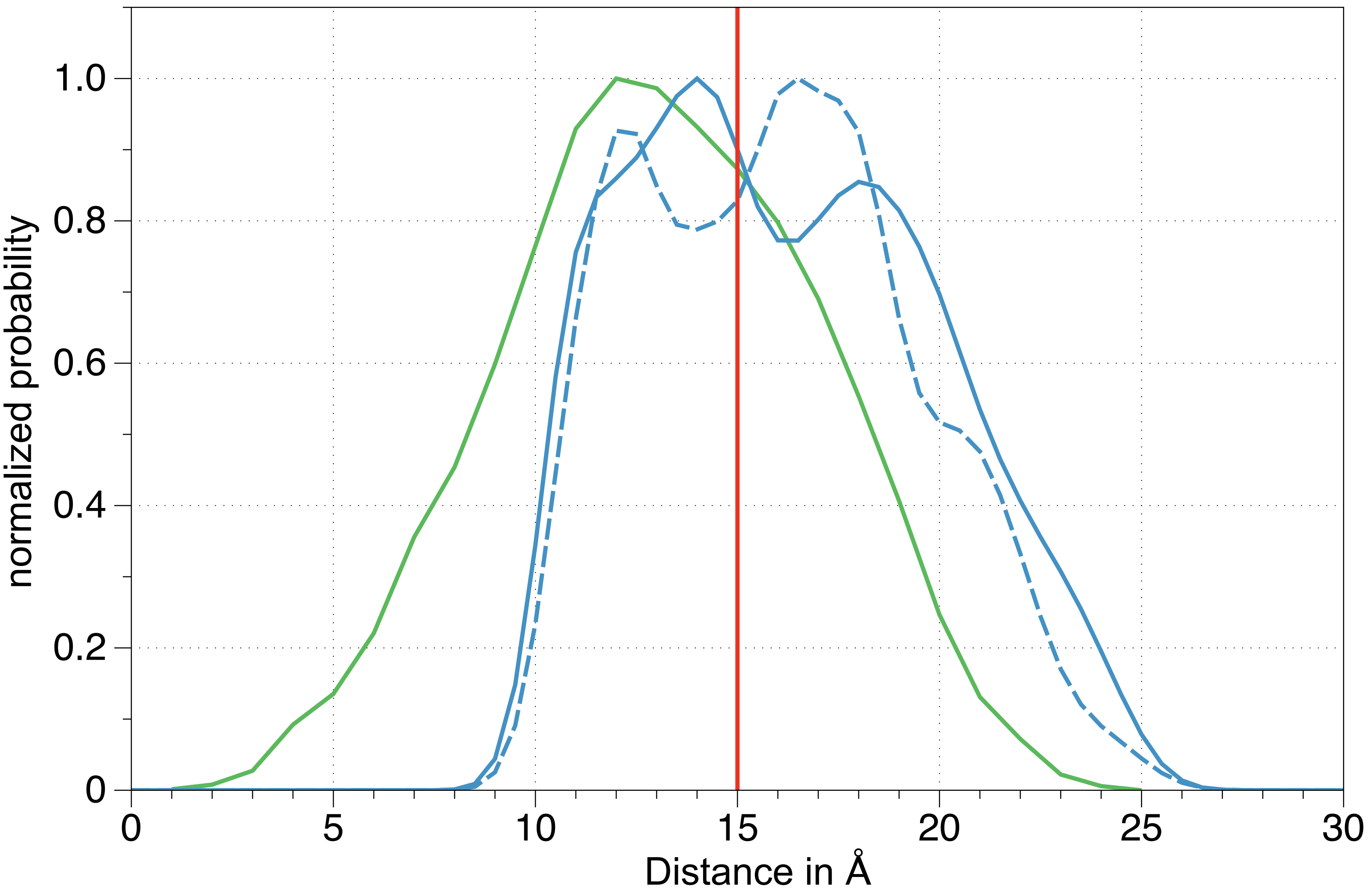

normalized probability
1.0
0.8
0.6
0.4
0.2
0
0
5
10
15
20
25
30
Distance in Å

T4L 60-90

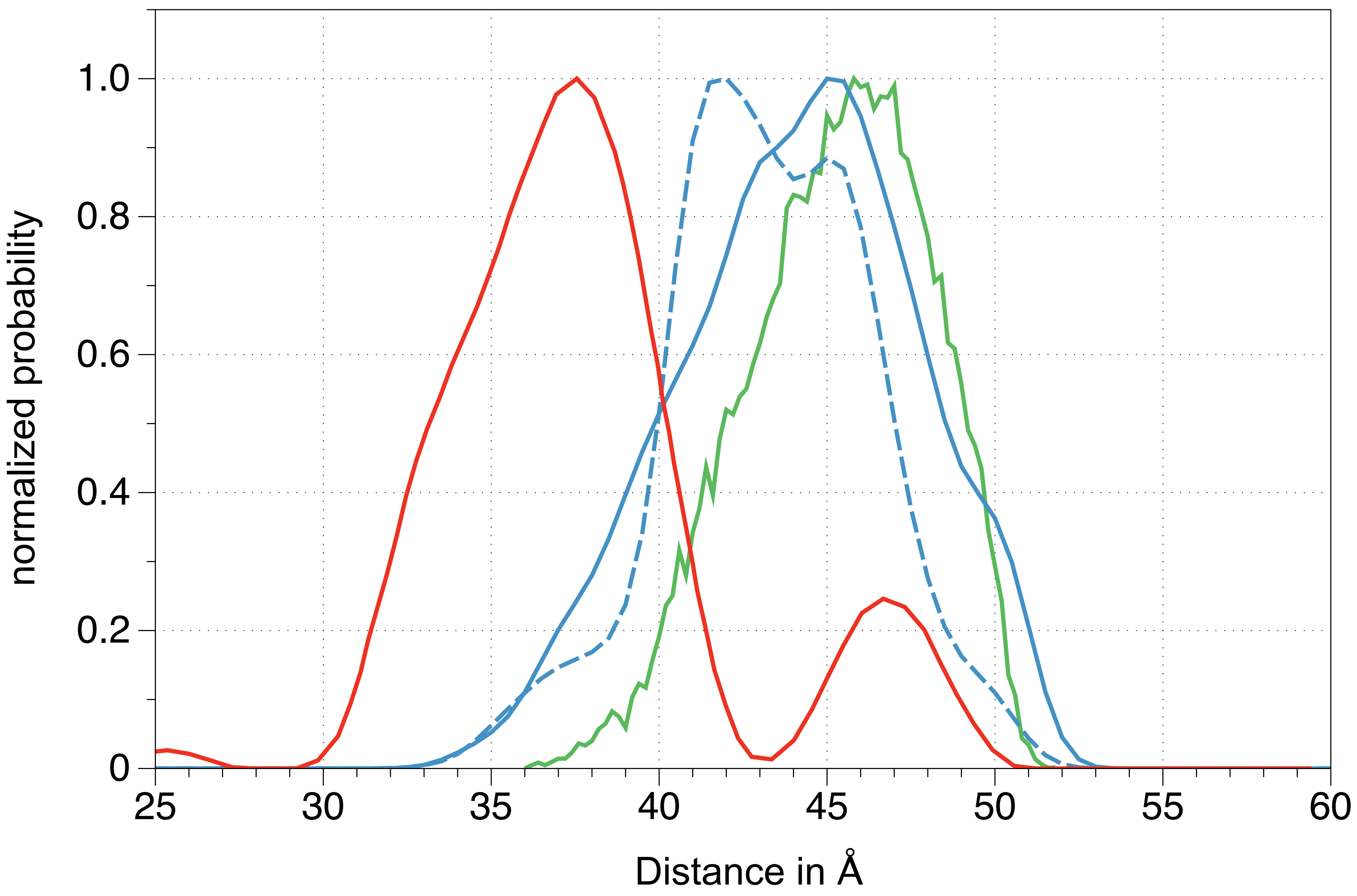

normalized probability
1.0
0.8
0.6
0.4
0.2
0
25
30
35
40
45
50
55
60
Distance in Å

T4L 60-109

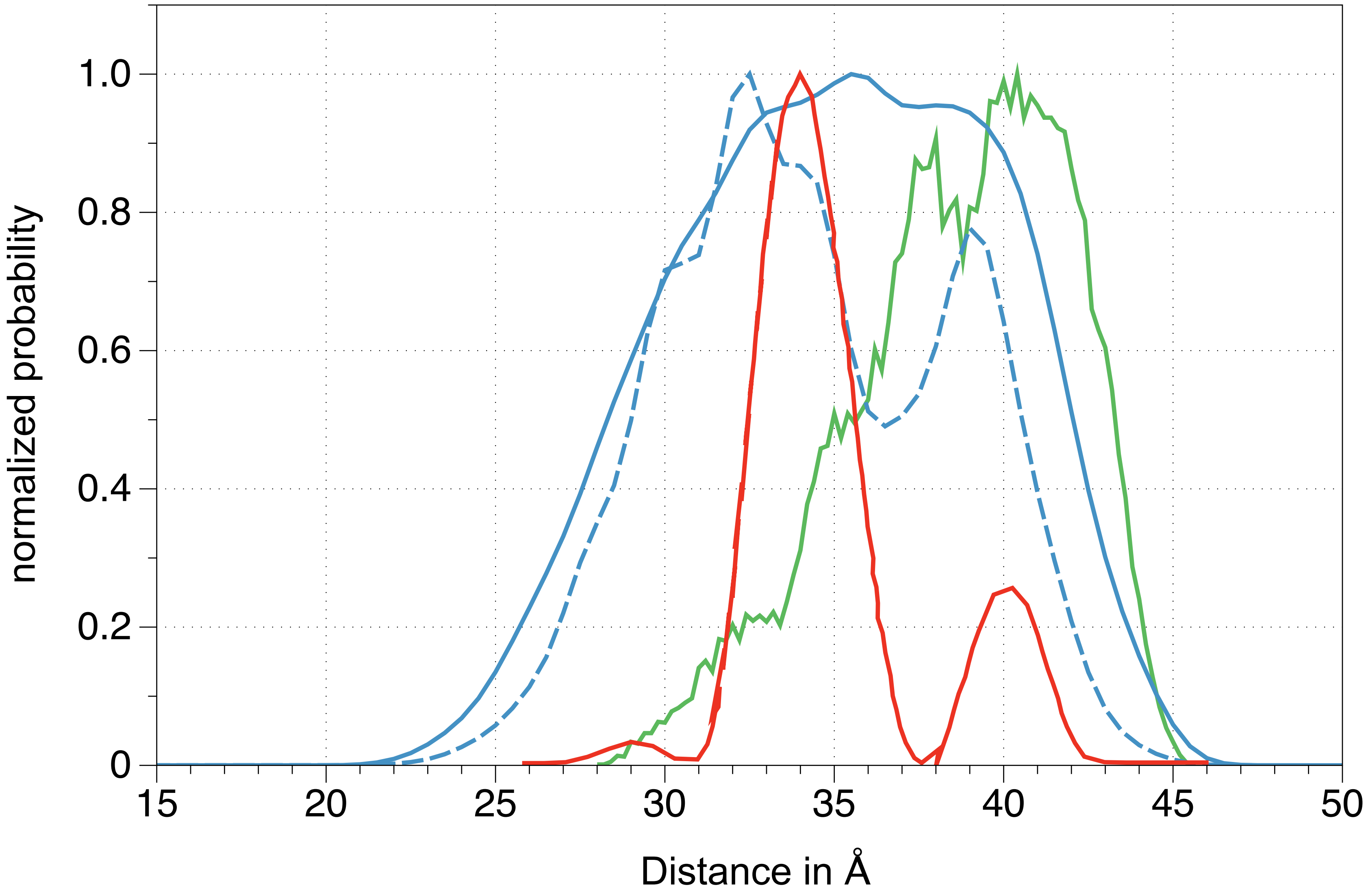

1.0
0.8
0.6
0.4
0.2
0
15
20
25
30
35
40
45
50
normalized probability
Distance in Å

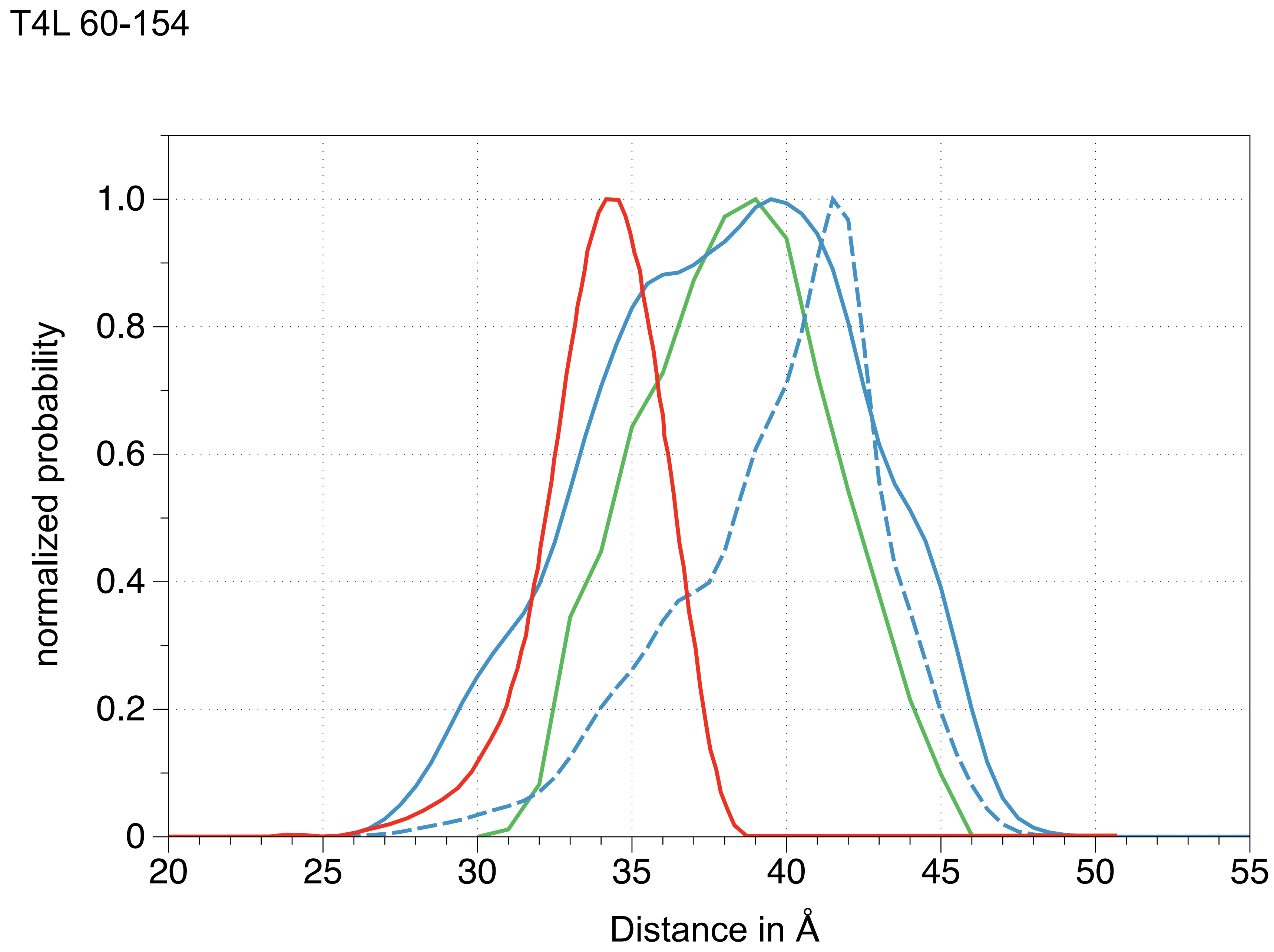
T4L 60-154
normalized probability
0
0.2
0.4
0.6
0.8
1.0
20
25
30
35
40
45
50
55
Distance in Å

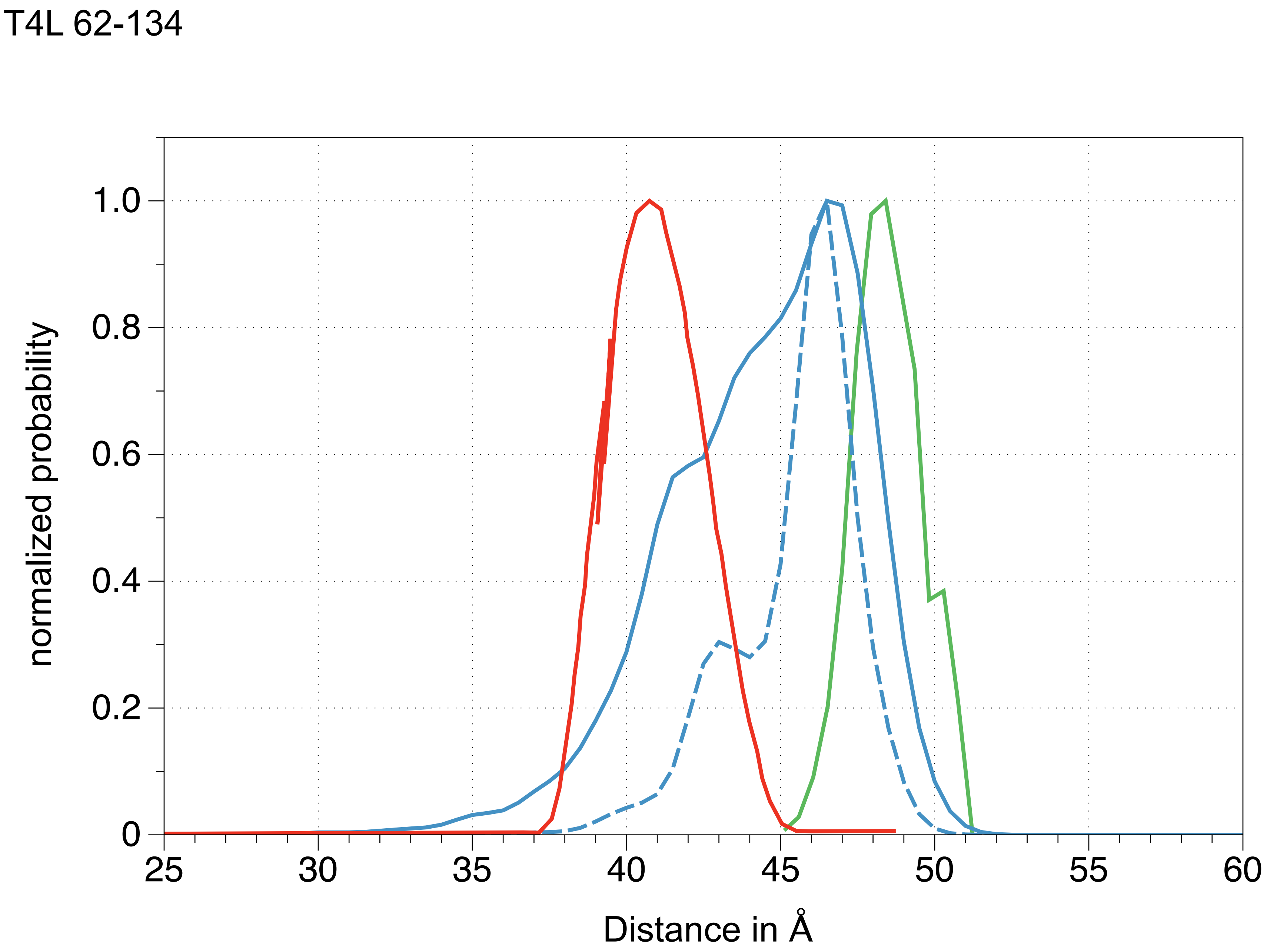

T4L 62-134
normalized probability
1.0
0.8
0.6
0.4
0.2
0
25
30
35
40
45
50
55
60
Distance in Å

T4L 64-122

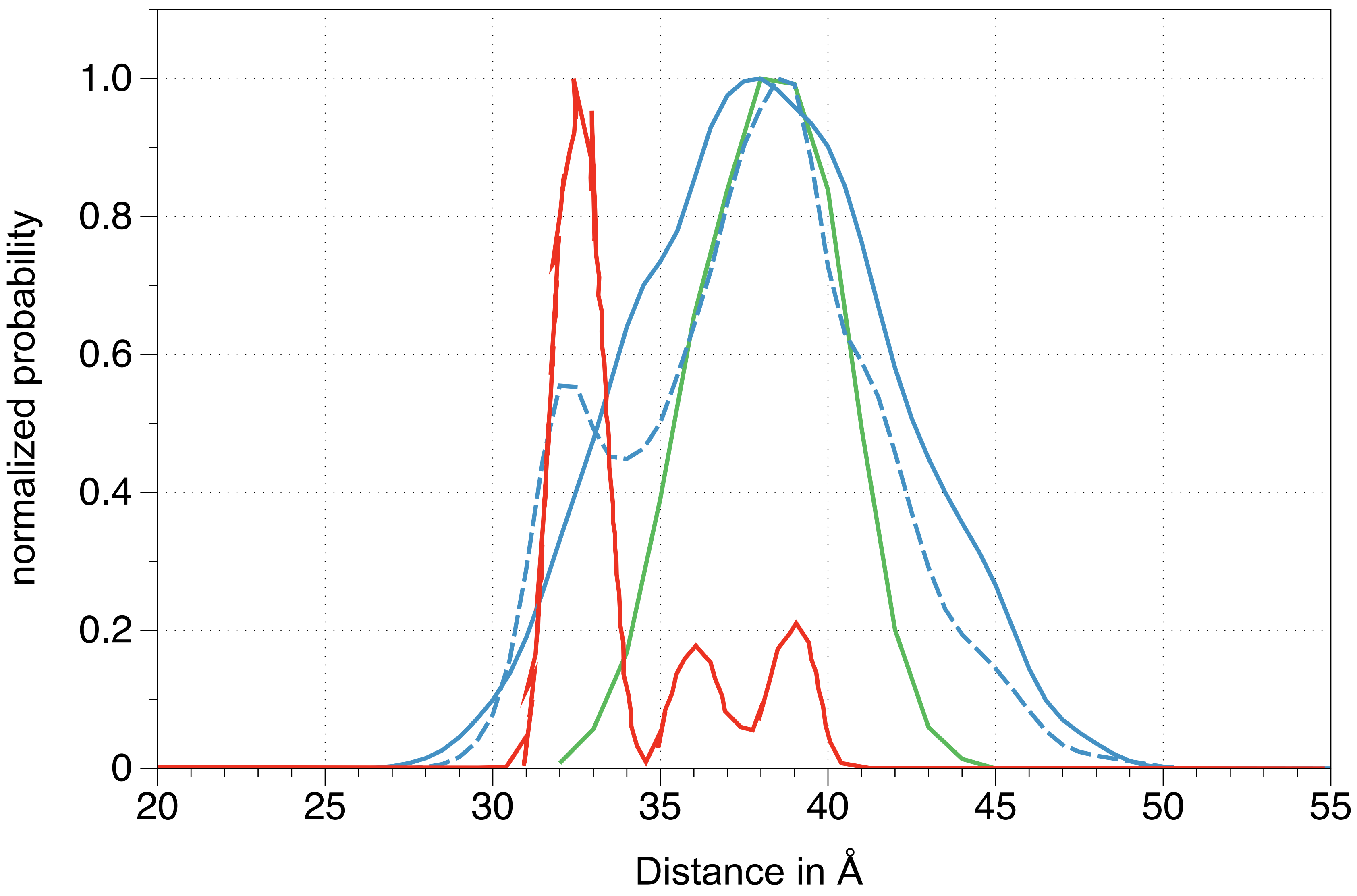
normalized probability
Distance in Å
0
0.2
0.4
0.6
0.8
1.0
20
25
30
35
40
45
50
55

T4L 82-94

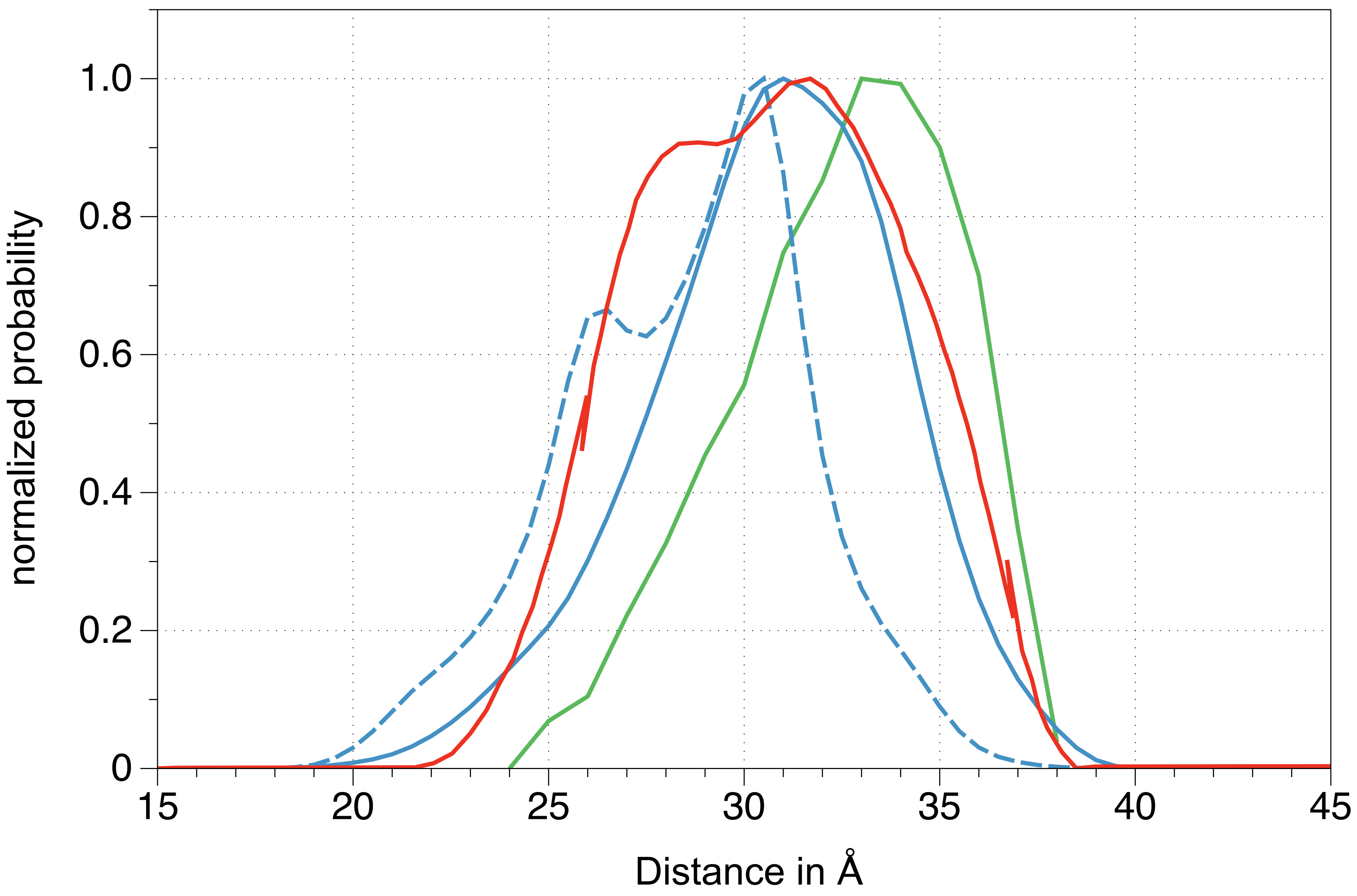

normalized probability
1.0
0.8
0.6
0.4
0.2
0
15
20
25
30
35
40
45
Distance in Å

T4L 82-132

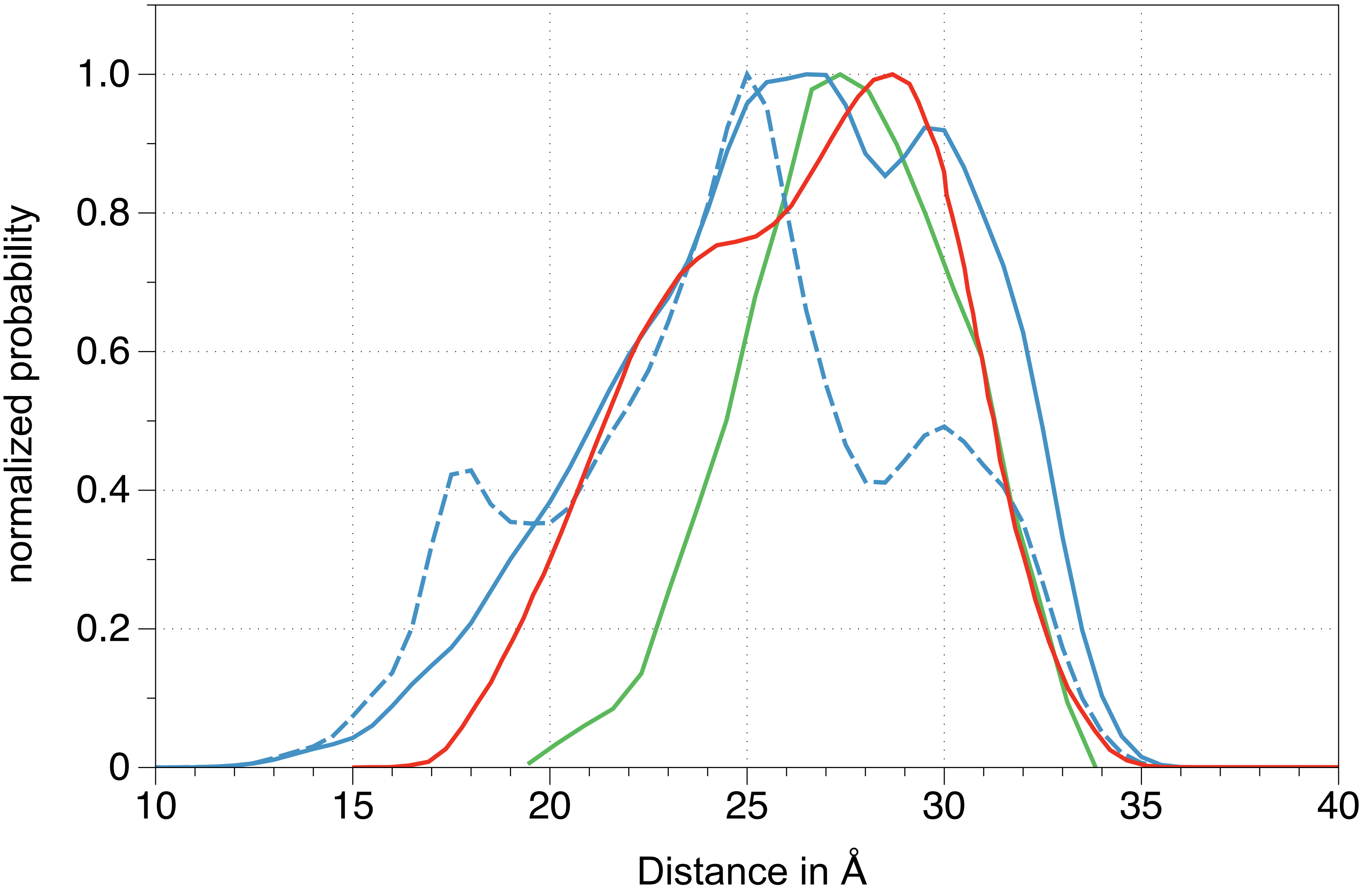

1.0
0.8
0.6
0.4
0.2
0
normalized probability
10
15
20
25
30
35
40
Distance in Å

T4L 82-155

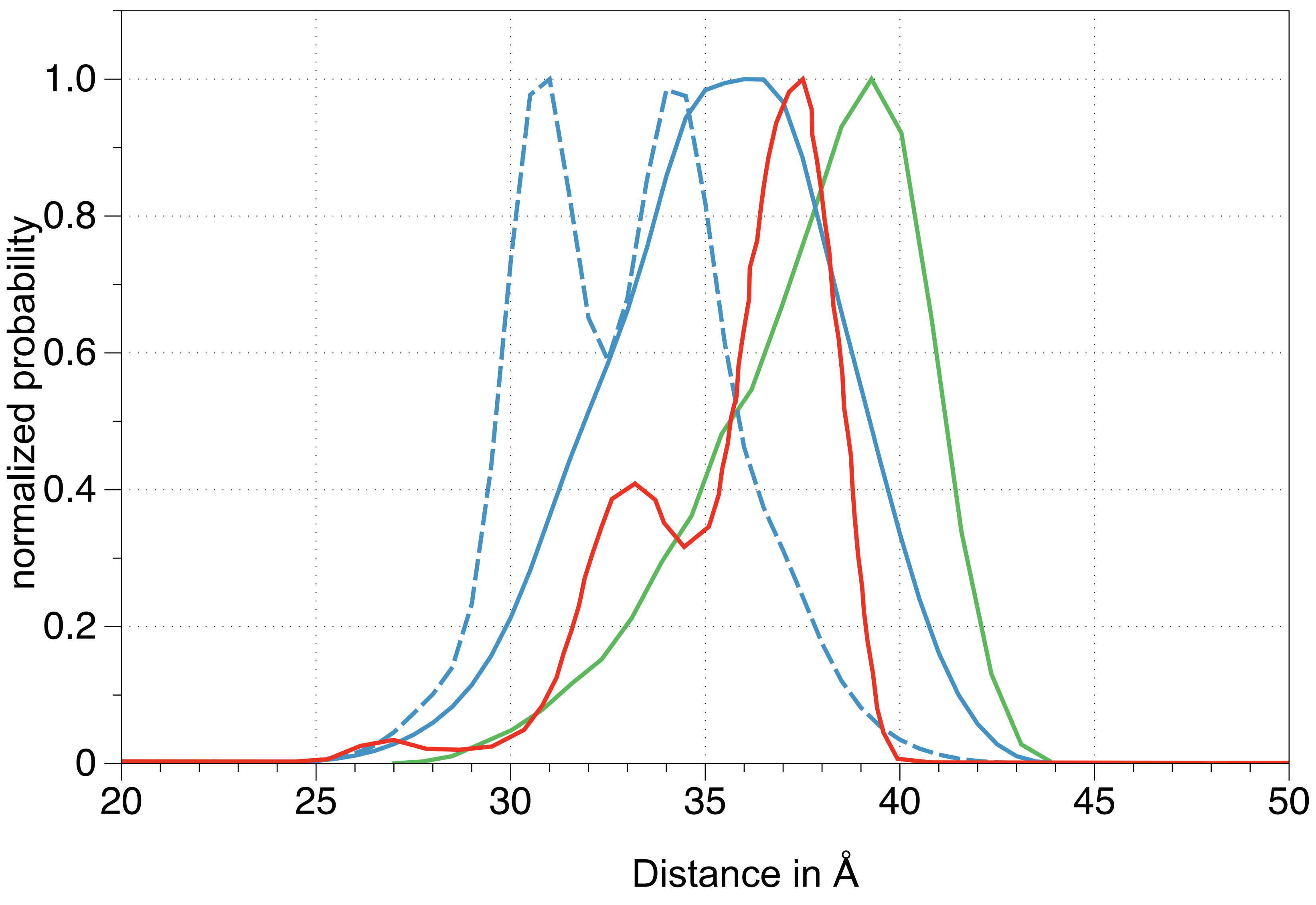

normalized probability
Distance in Å
0
0.2
0.4
0.6
0.8
1.0
20
25
30
35
40
45
50

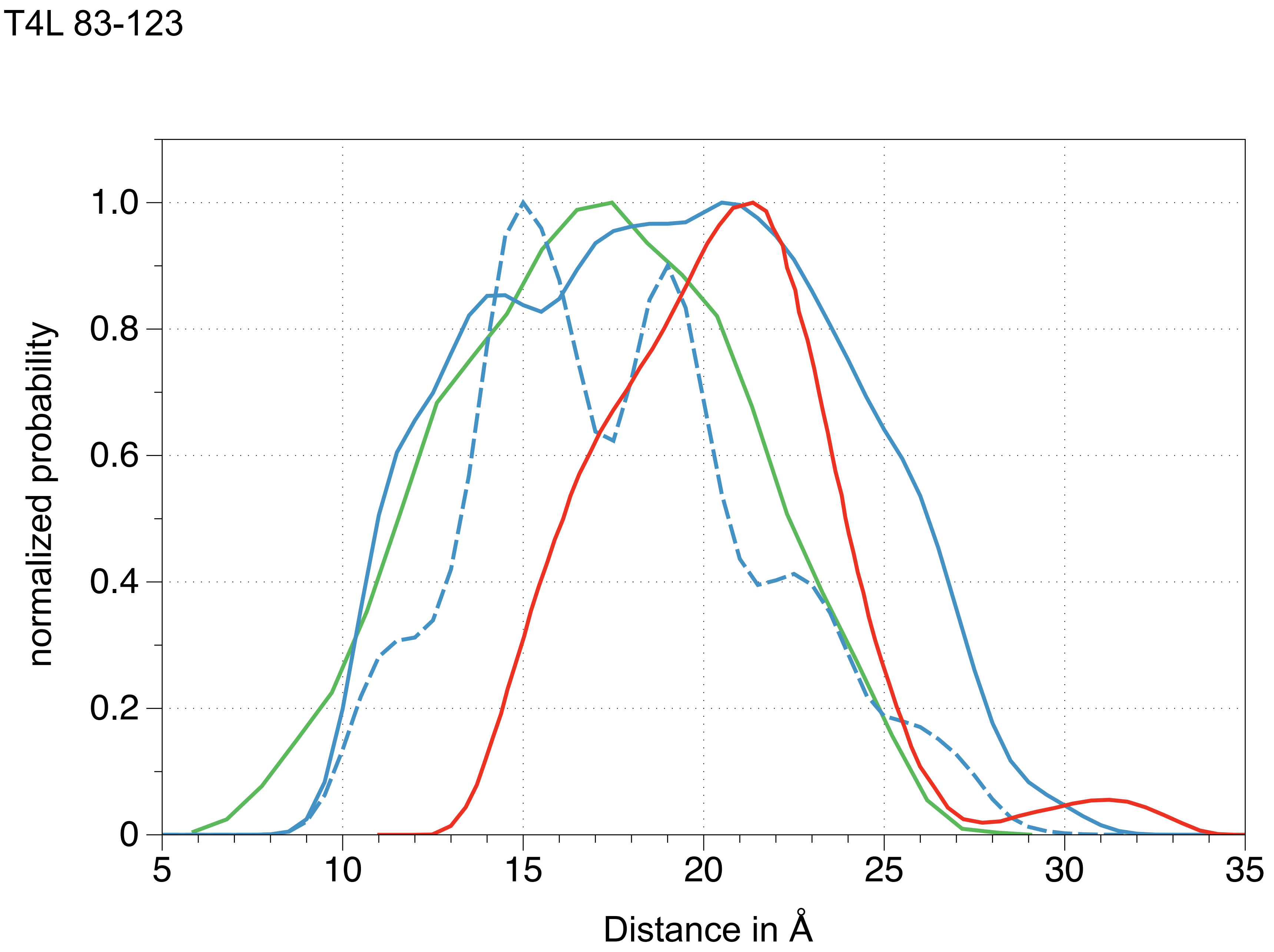
T4L 83-123
normalized probability
0
0.2
0.4
0.6
0.8
1.0
5
10
15
20
25
30
35
Distance in Å

T4L 93-112

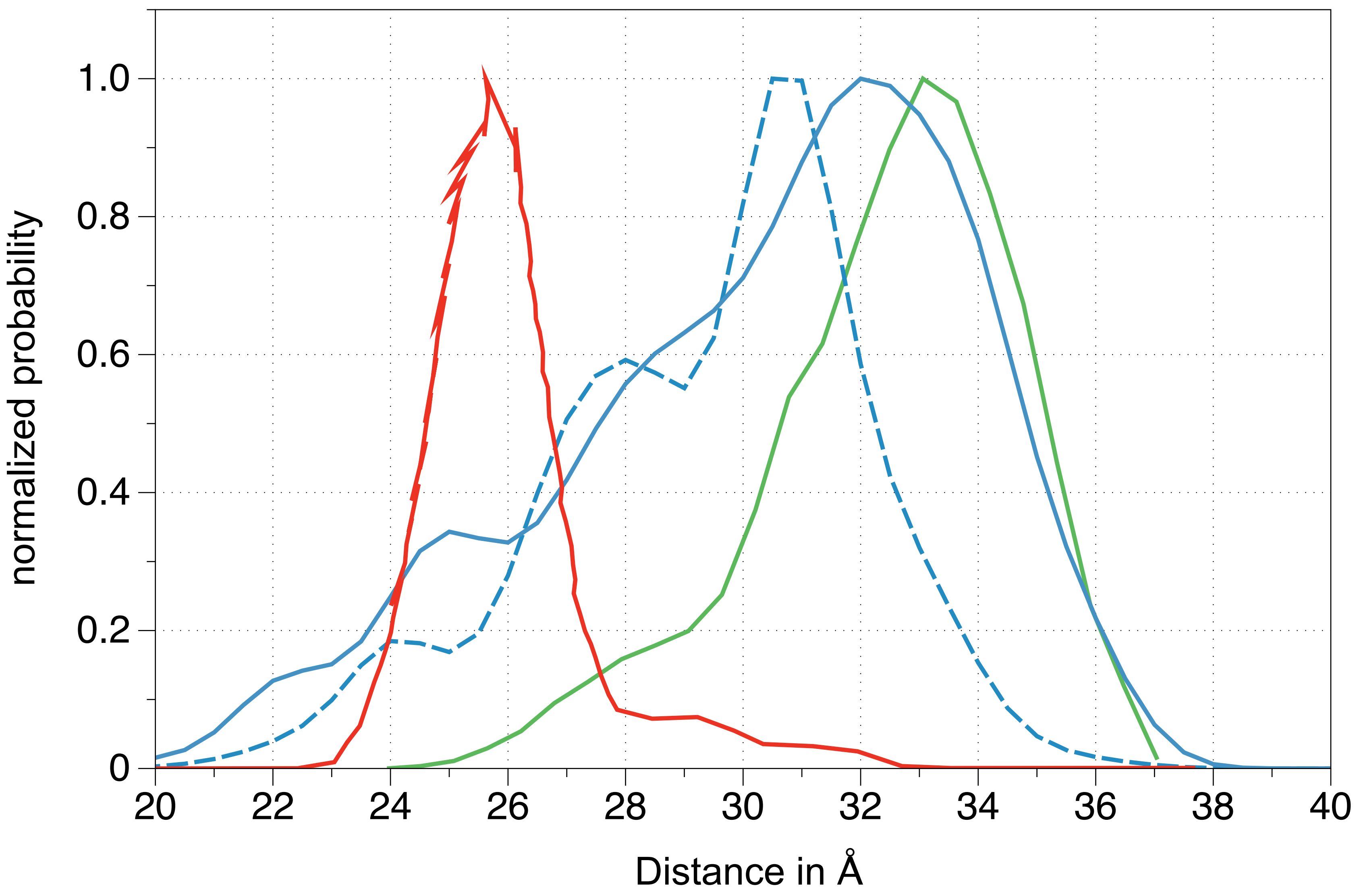
1.0
0.8
0.6
0.4
0.2
0
normalized probability
20
22
24
26
28
30
32
34
36
38
40
Distance in Å

T4L 93-123

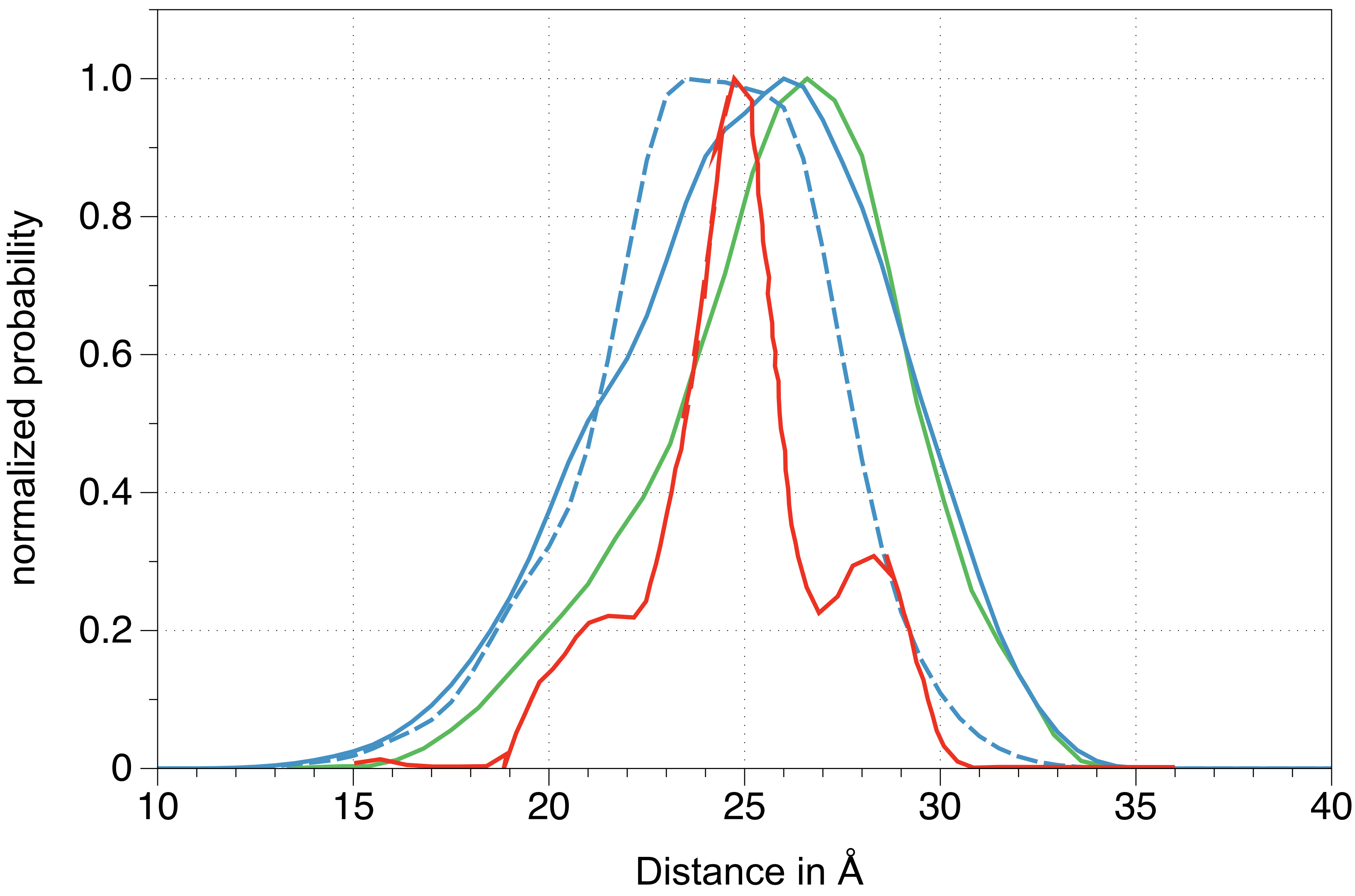

normalized probability
1.0
0.8
0.6
0.4
0.2
0
10
15
20
25
30
35
40
Distance in Å

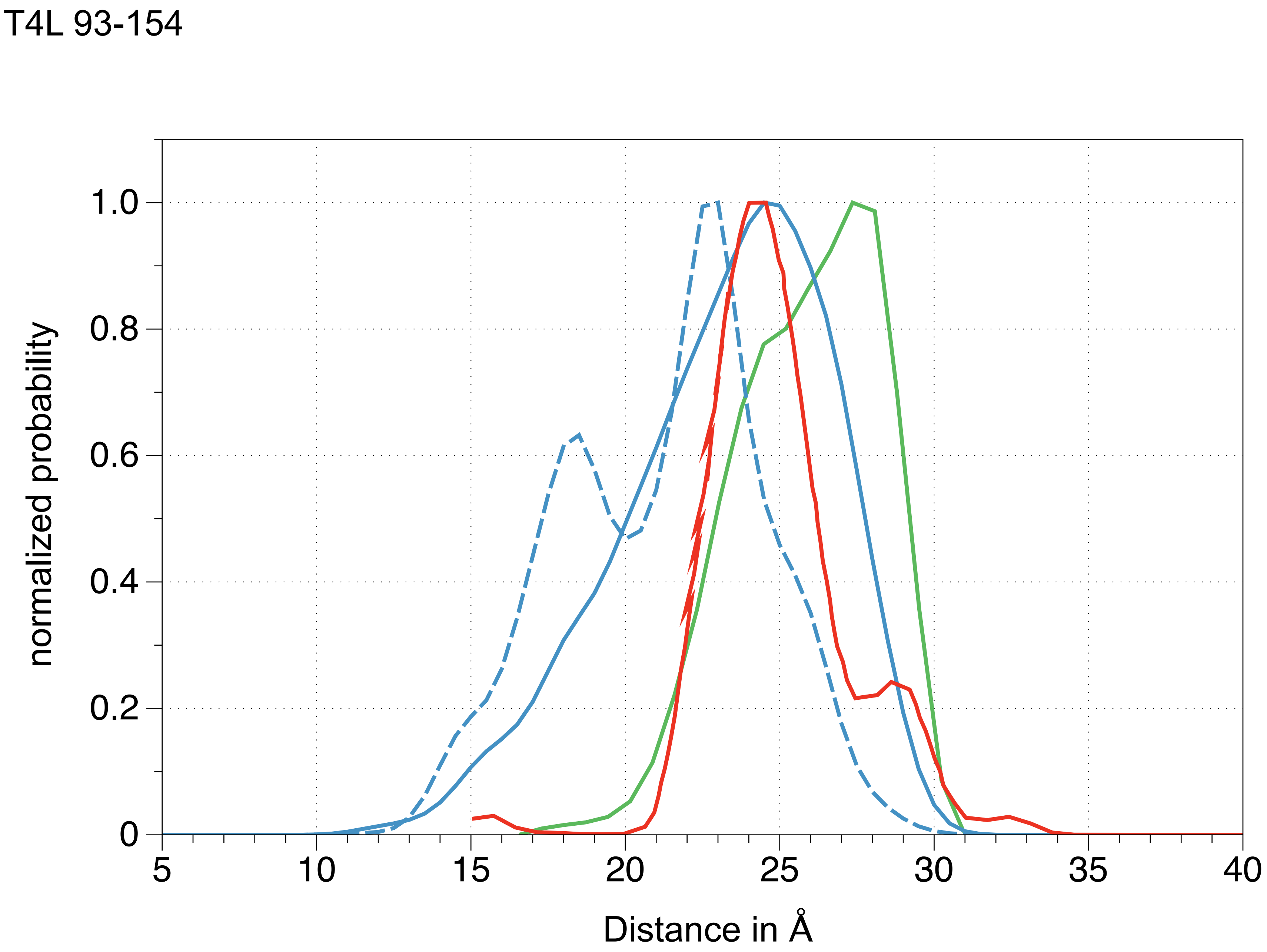

T4L 93-154
normalized probability
1.0
0.8
0.6
0.4
0.2
0
5
10
15
20
25
30
35
40
Distance in Å

T4L 94-132

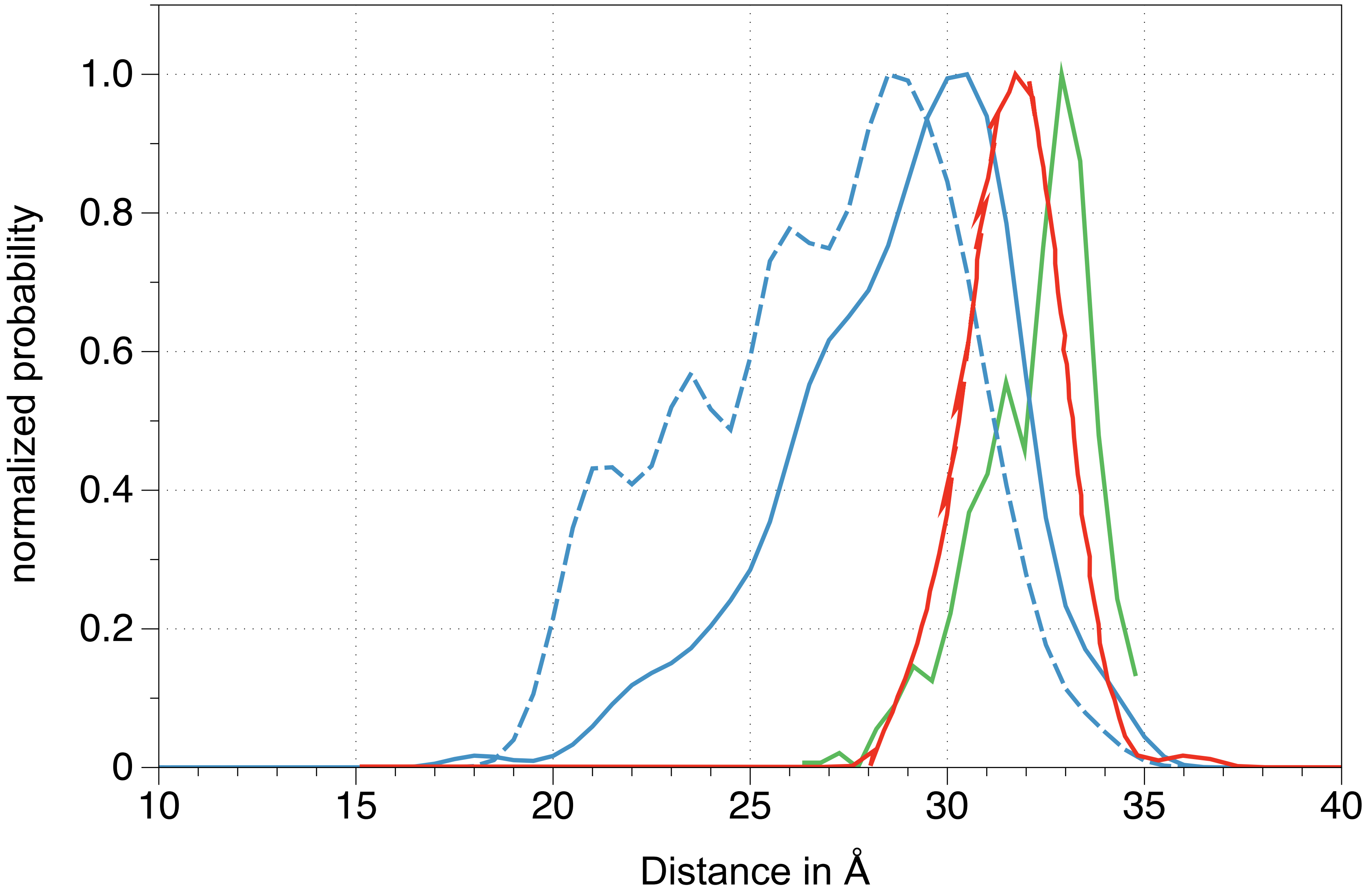

normalized probability
0
0.2
0.4
0.6
0.8
1.0
10
15
20
25
30
35
40
Distance in Å

T4L 108-155

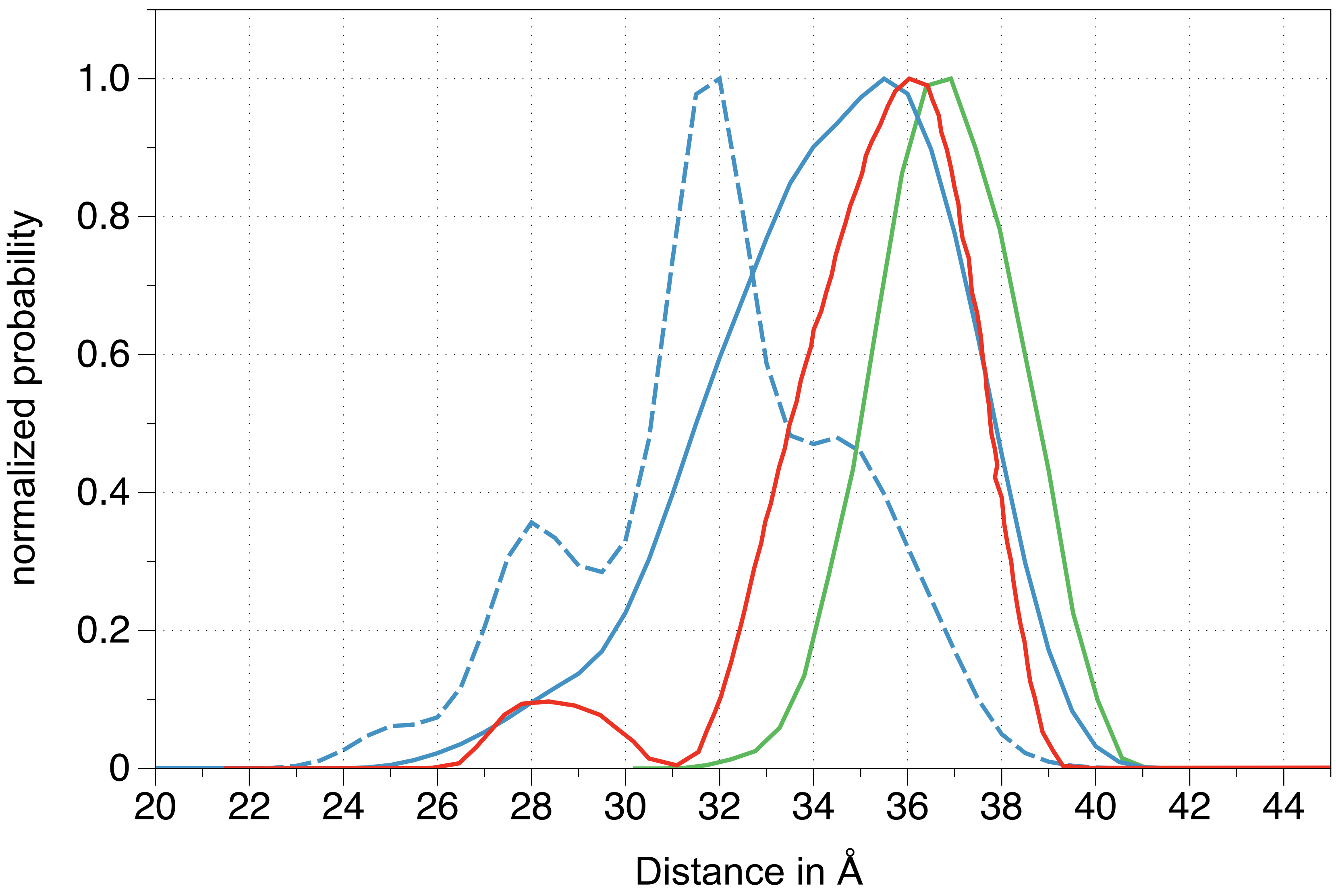

normalized probability
1.0
0.8
0.6
0.4
0.2
0
20
22
24
26
28
30
32
34
36
38
40
42
44
Distance in Å

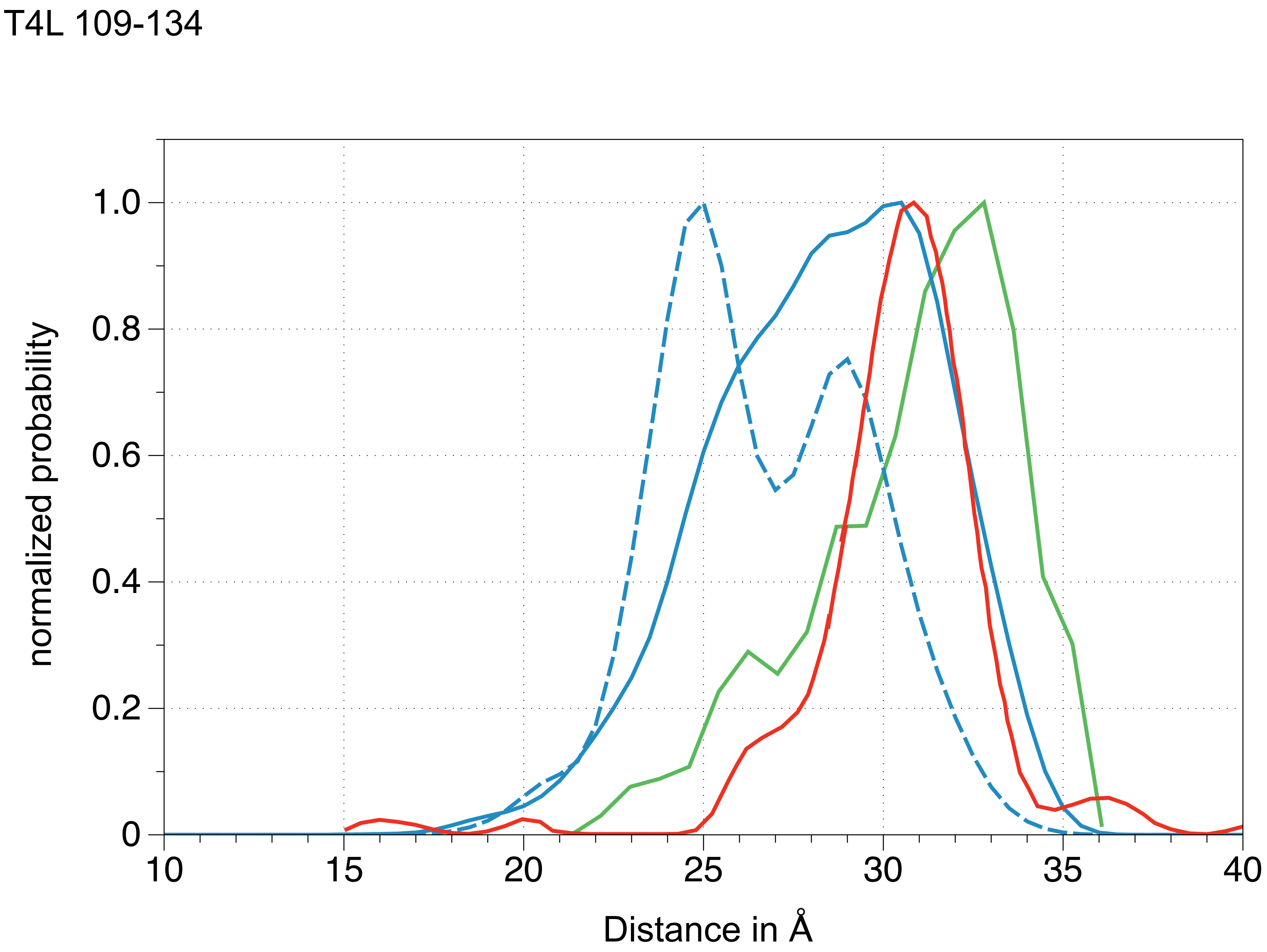
T4L 109-134
normalized probability
0
0.2
0.4
0.6
0.8
1.0
10
15
20
25
30
35
40
Distance in Å

T4L 115-155

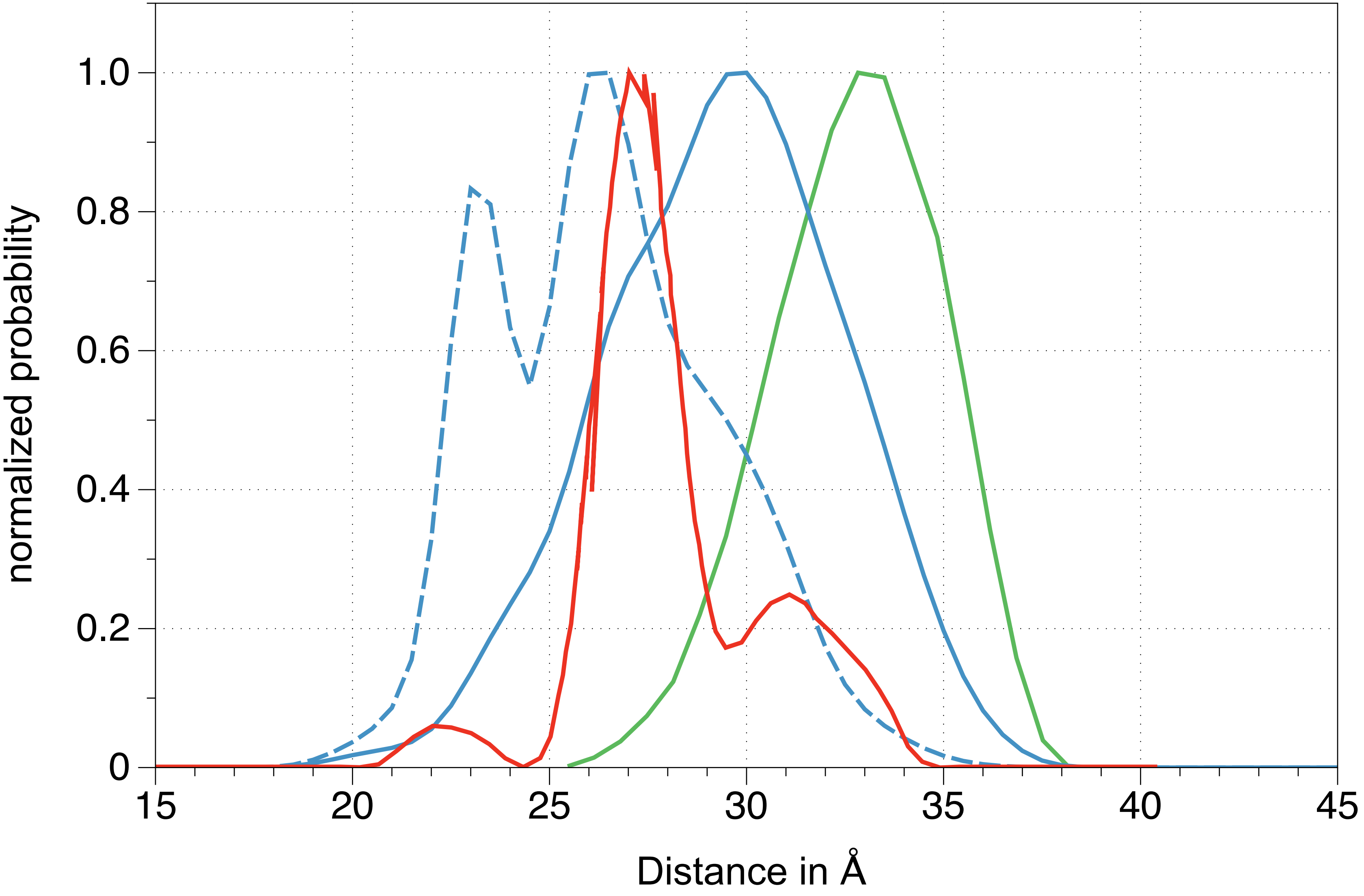

normalized probability
1.0
0.8
0.6
0.4
0.2
0
15
20
25
30
35
40
45
Distance in Å

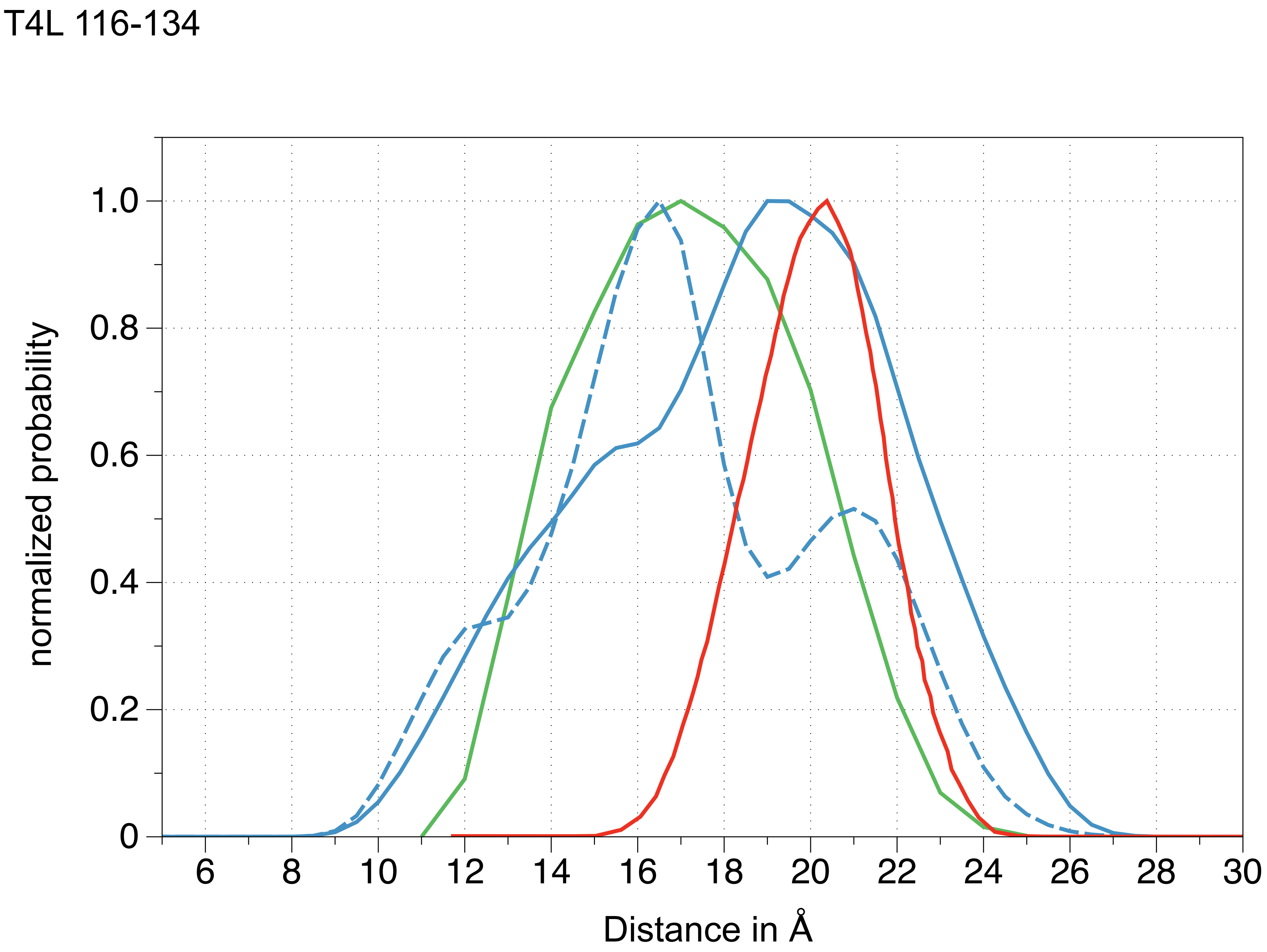
T4L 116-134
normalized probability
0
0.2
0.4
0.6
0.8
1.0
6
8
10
12
14
16
18
20
22
24
26
28
30
Distance in Å

H3V46

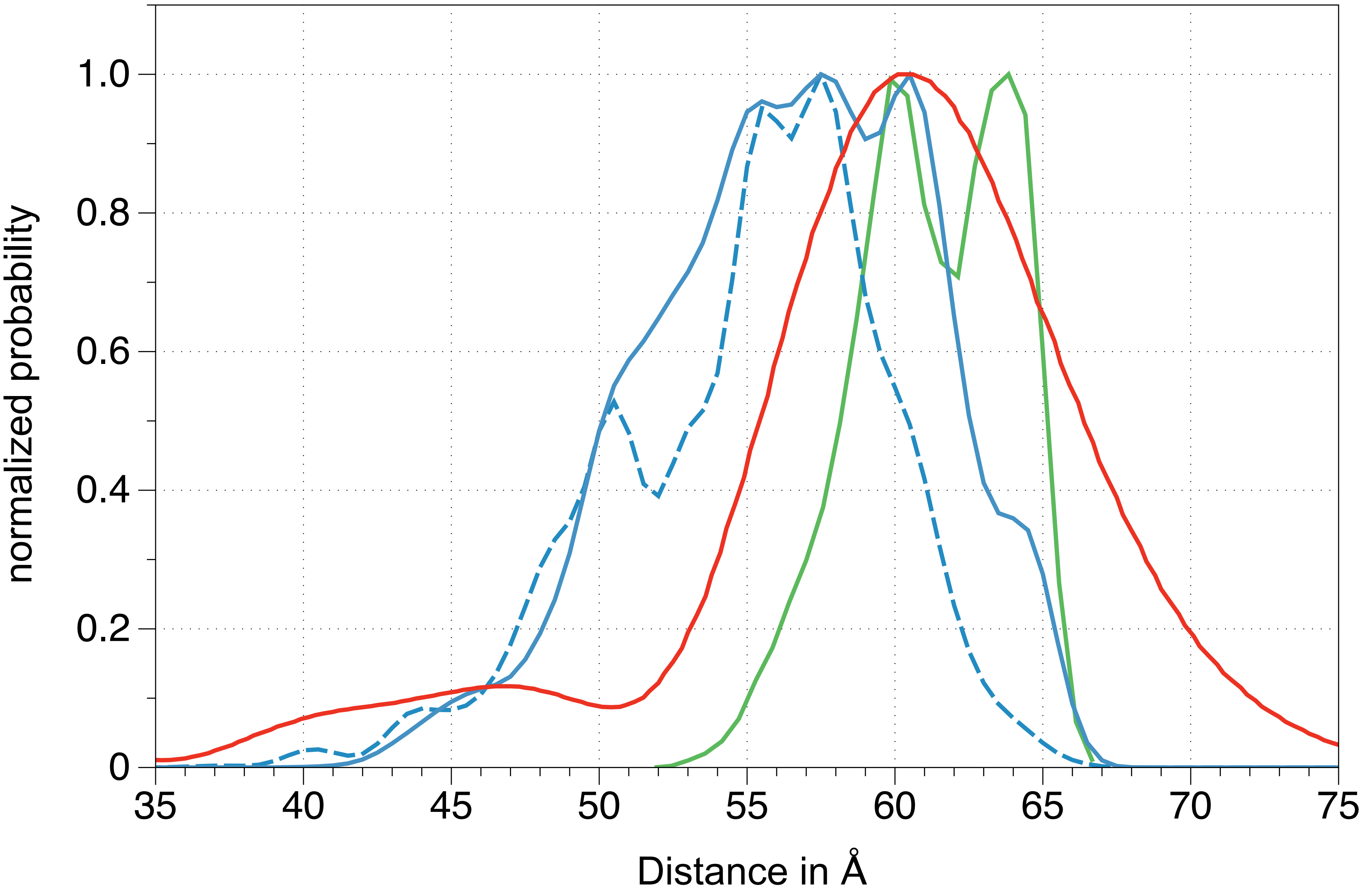
1.0
0.8
0.6
0.4
0.2
0
normalized probability
35
40
45
50
55
60
65
70
75
Distance in Å

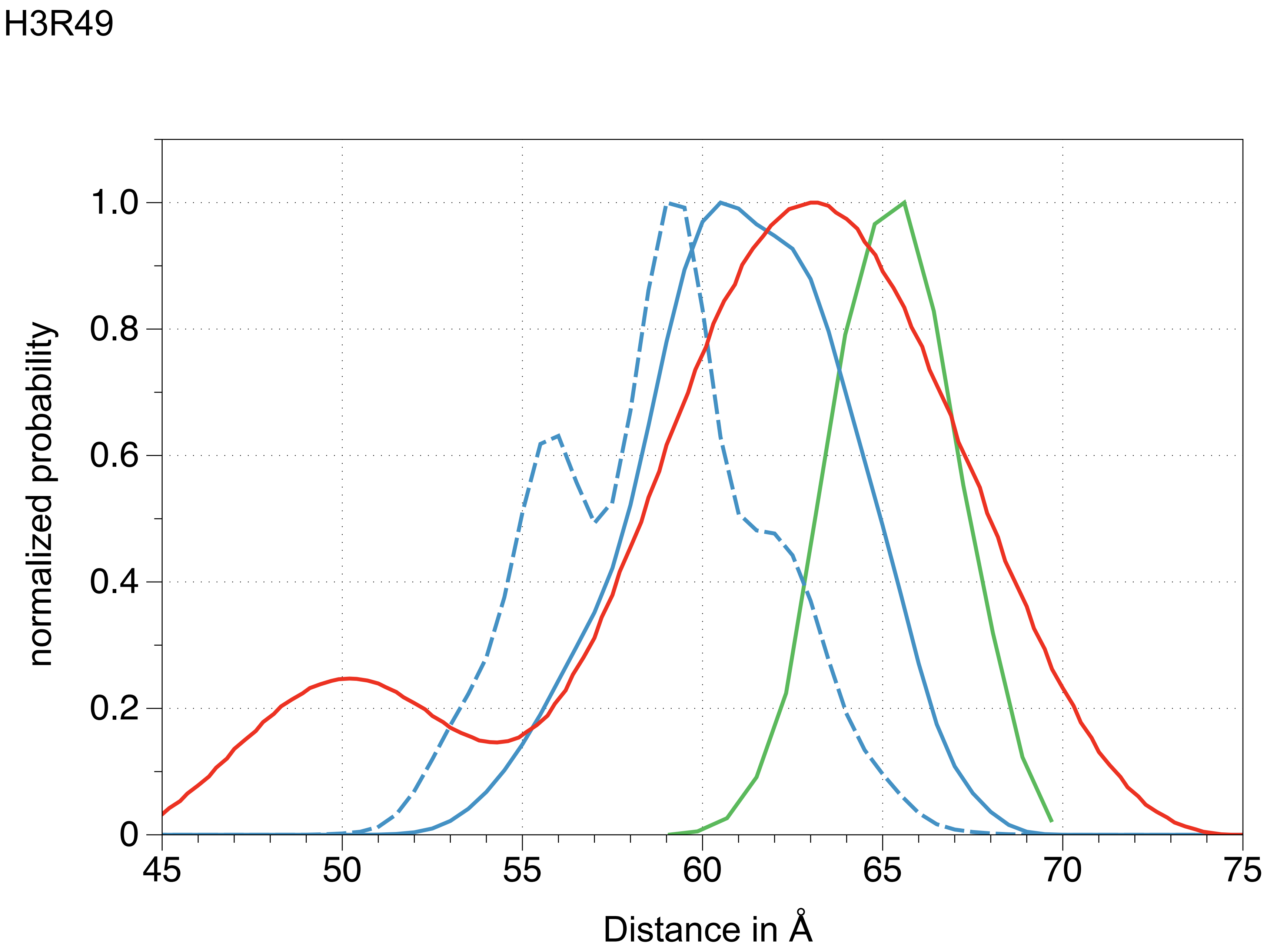
H3R49
normalized probability
1.0
0.8
0.6
0.4
0.2
0
45
50
55
60
65
70
75
Distance in Å

H3L65

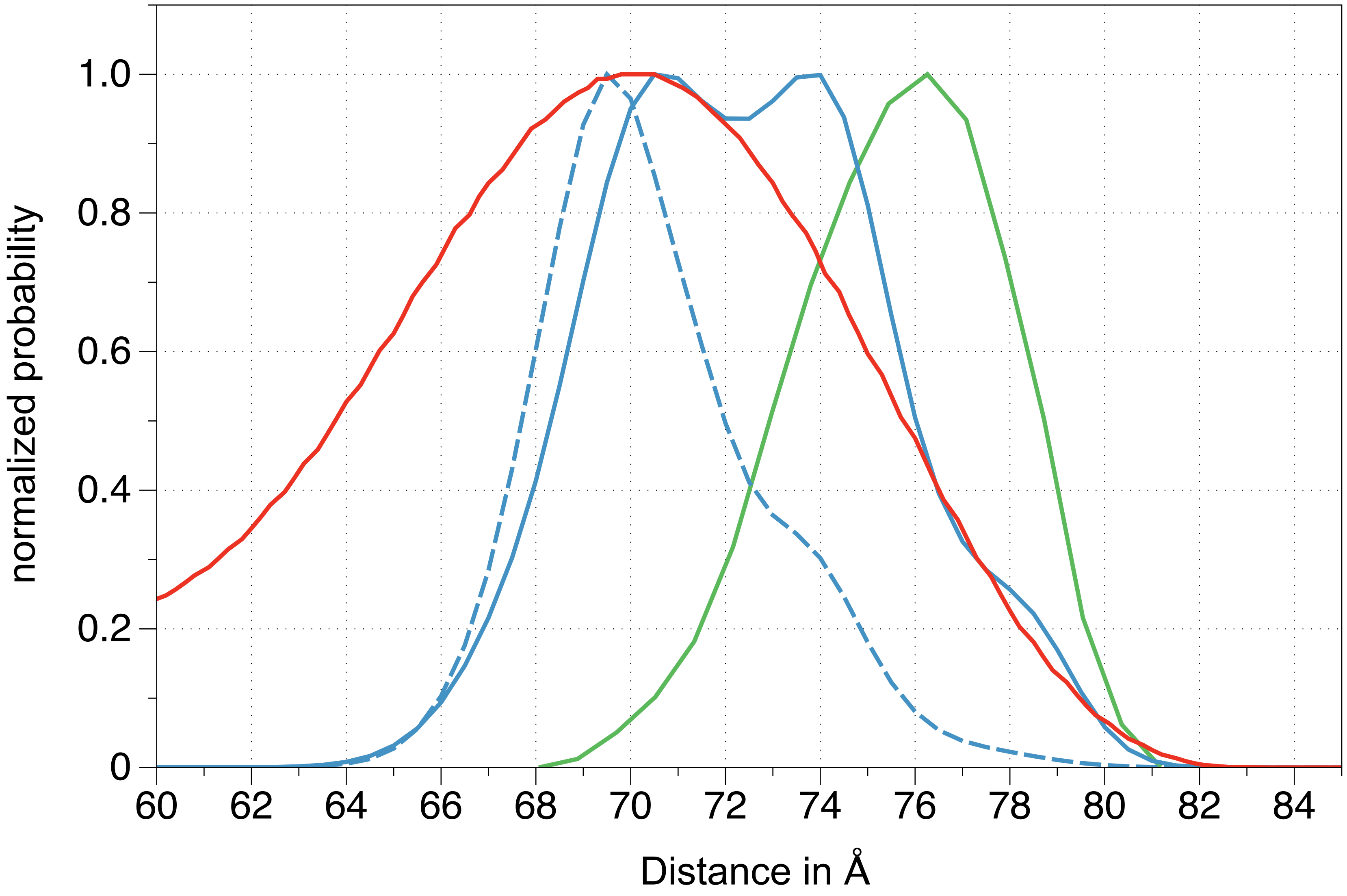

normalized probability
1.0
0.8
0.6
0.4
0.2
0
60
62
64
66
68
70
72
74
76
78
80
82
84
Distance in Å

H3Q76

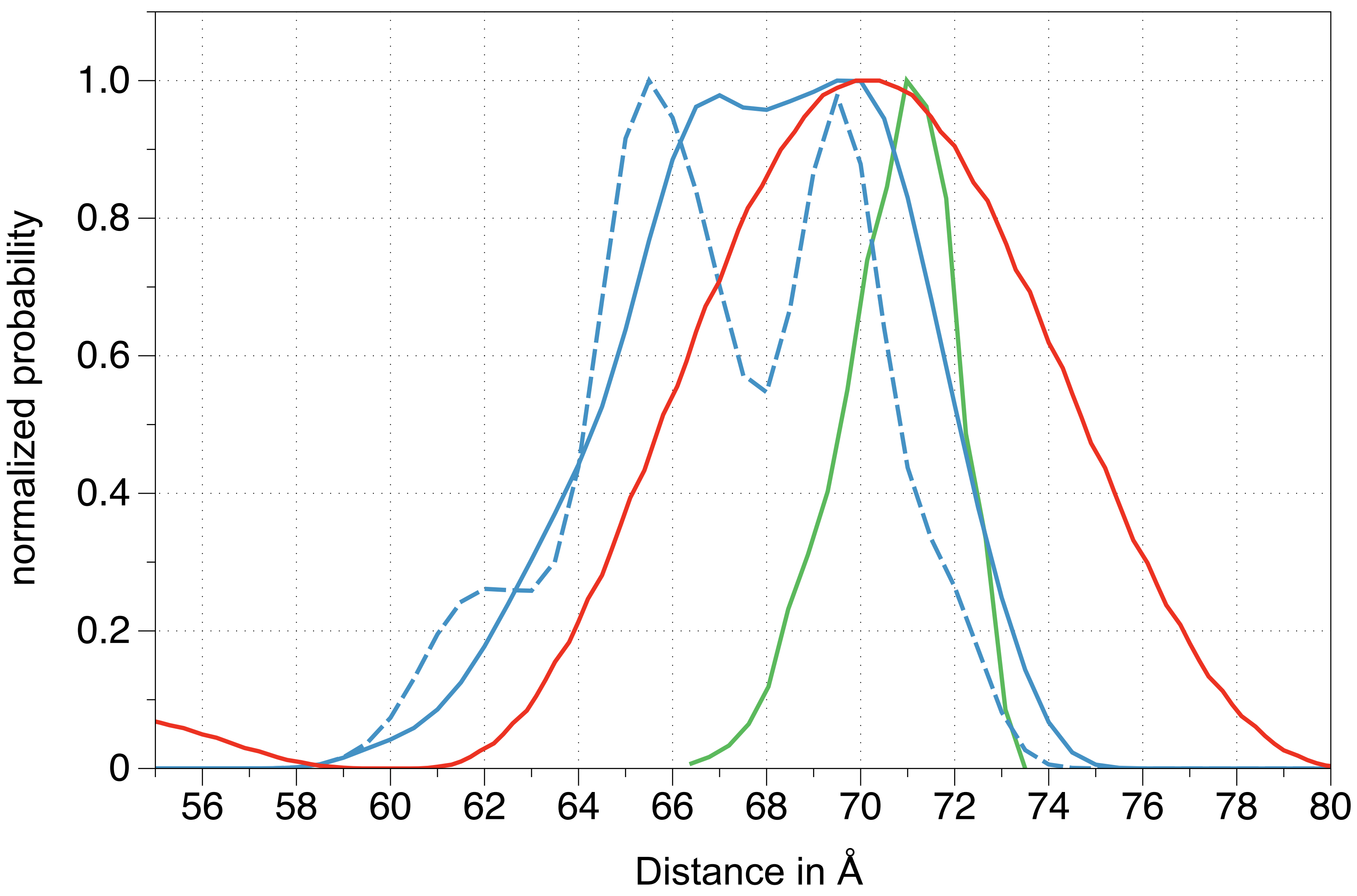

normalized probability
1.0
0.8
0.6
0.4
0.2
0
56
58
60
62
64
66
68
70
72
74
76
78
80
Distance in Å

H3M90

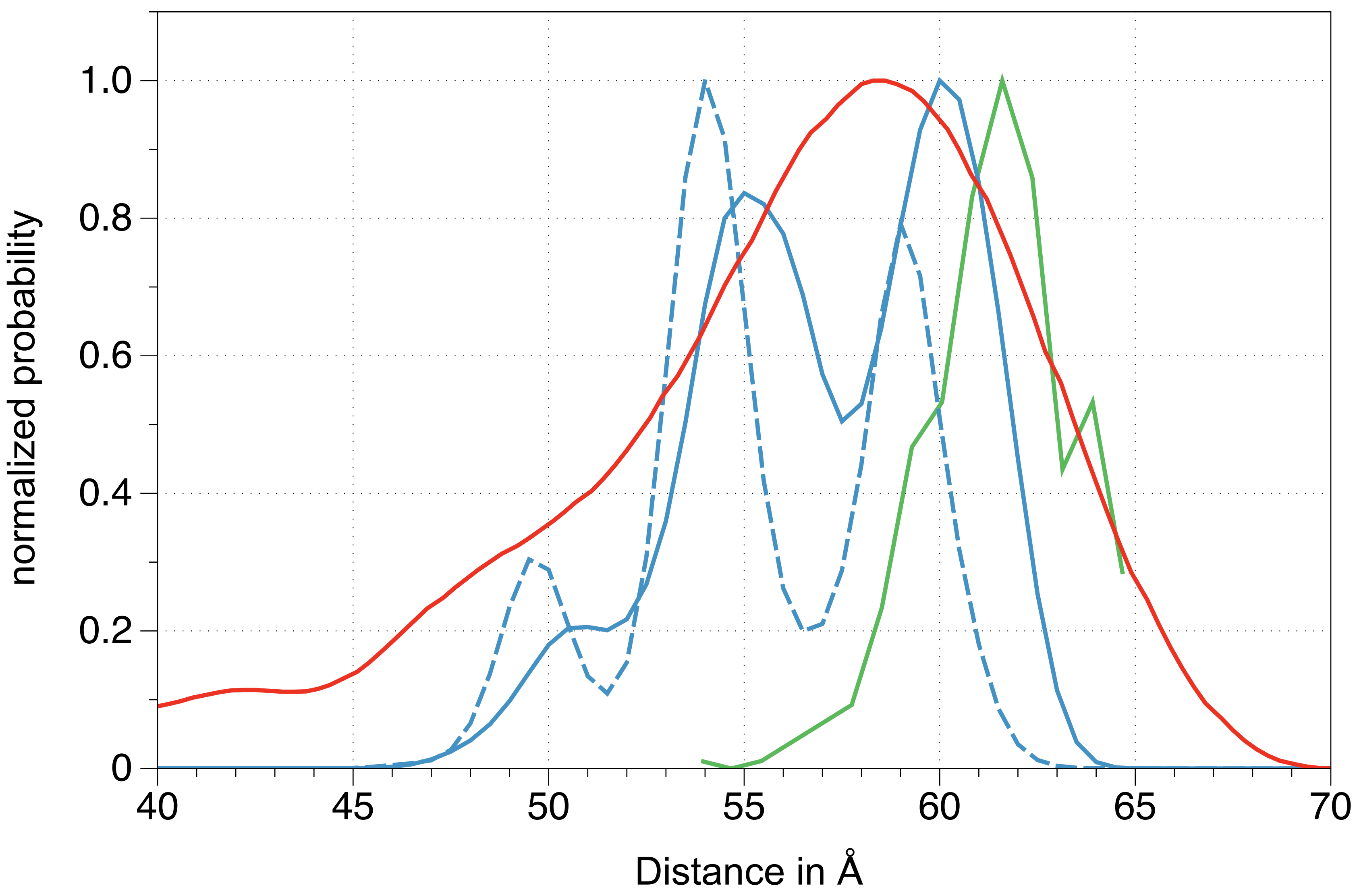

1.0
0.8
0.6
0.4
0.2
0
40
45
50
55
60
65
70
normalized probability
Distance in Å

H3Q125

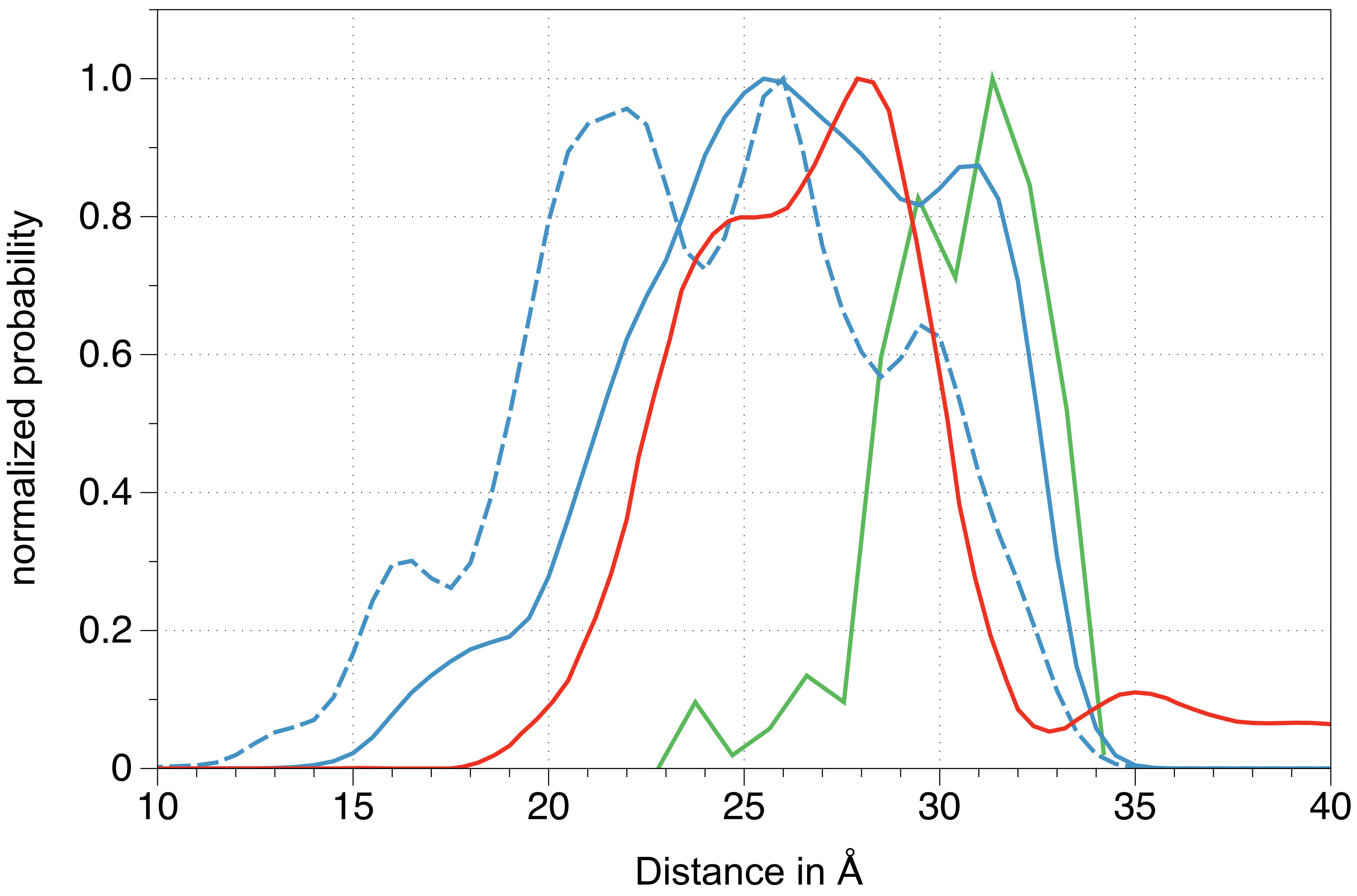
normalized probability
1.0
0.8
0.6
0.4
0.2
0
10
15
20
25
30
35
40
Distance in Å

H4N25

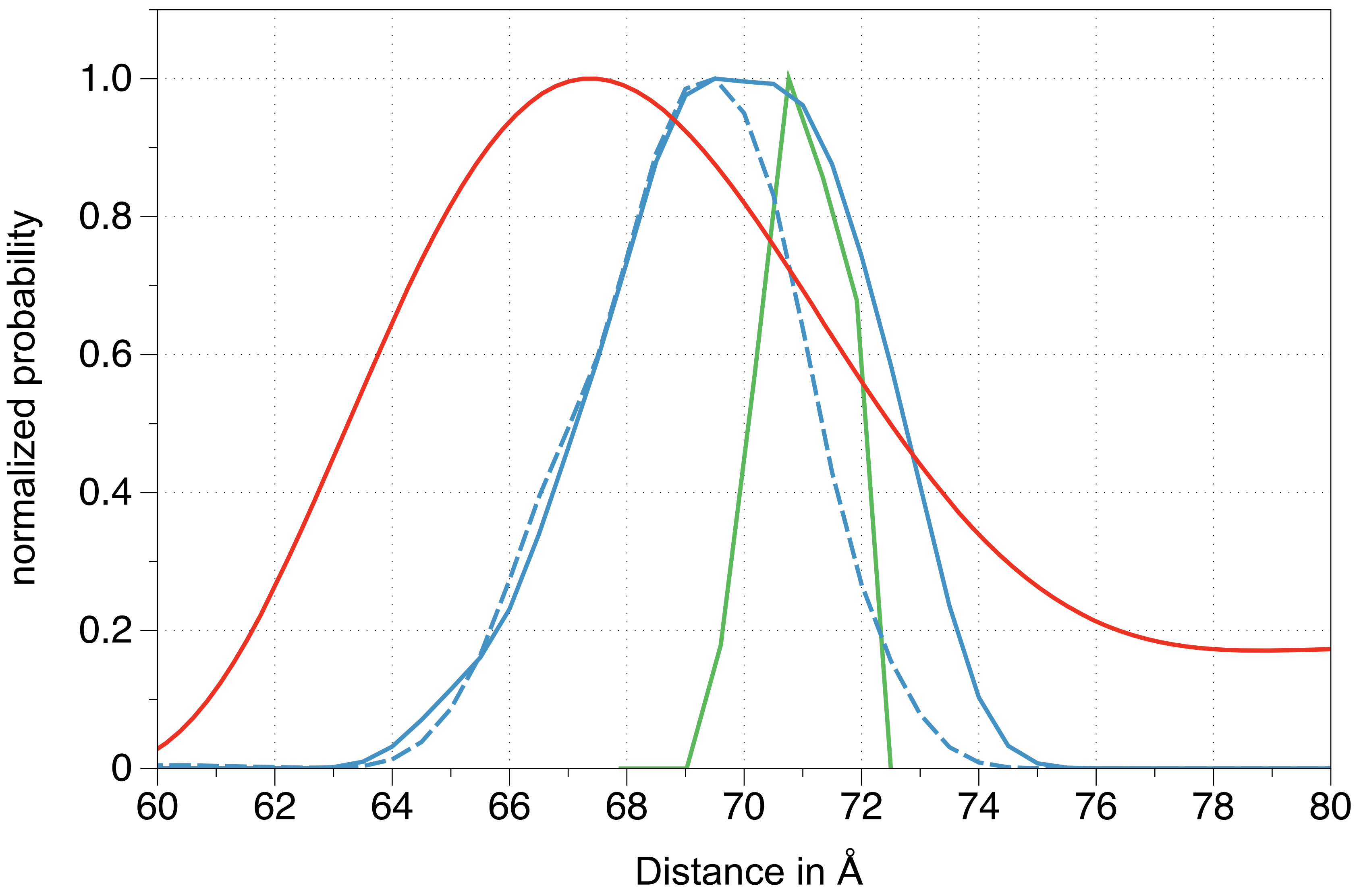

1.0
0.8
0.6
0.4
0.2
0
normalized probability
60
62
64
66
68
70
72
74
76
78
80
Distance in Å

H4T30

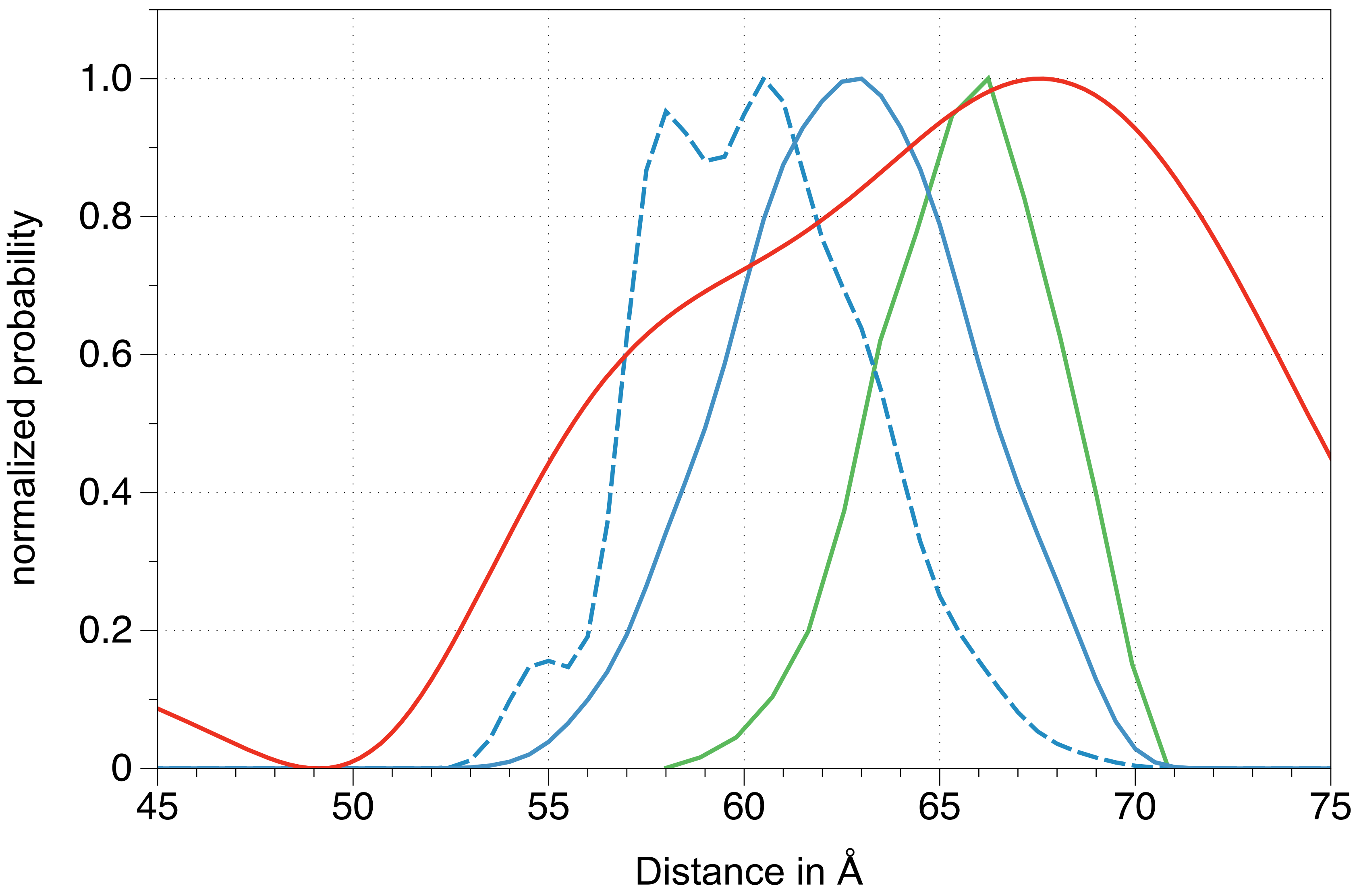

normalized probability
1.0
0.8
0.6
0.4
0.2
0
45
50
55
60
65
70
75
Distance in Å

H4R45

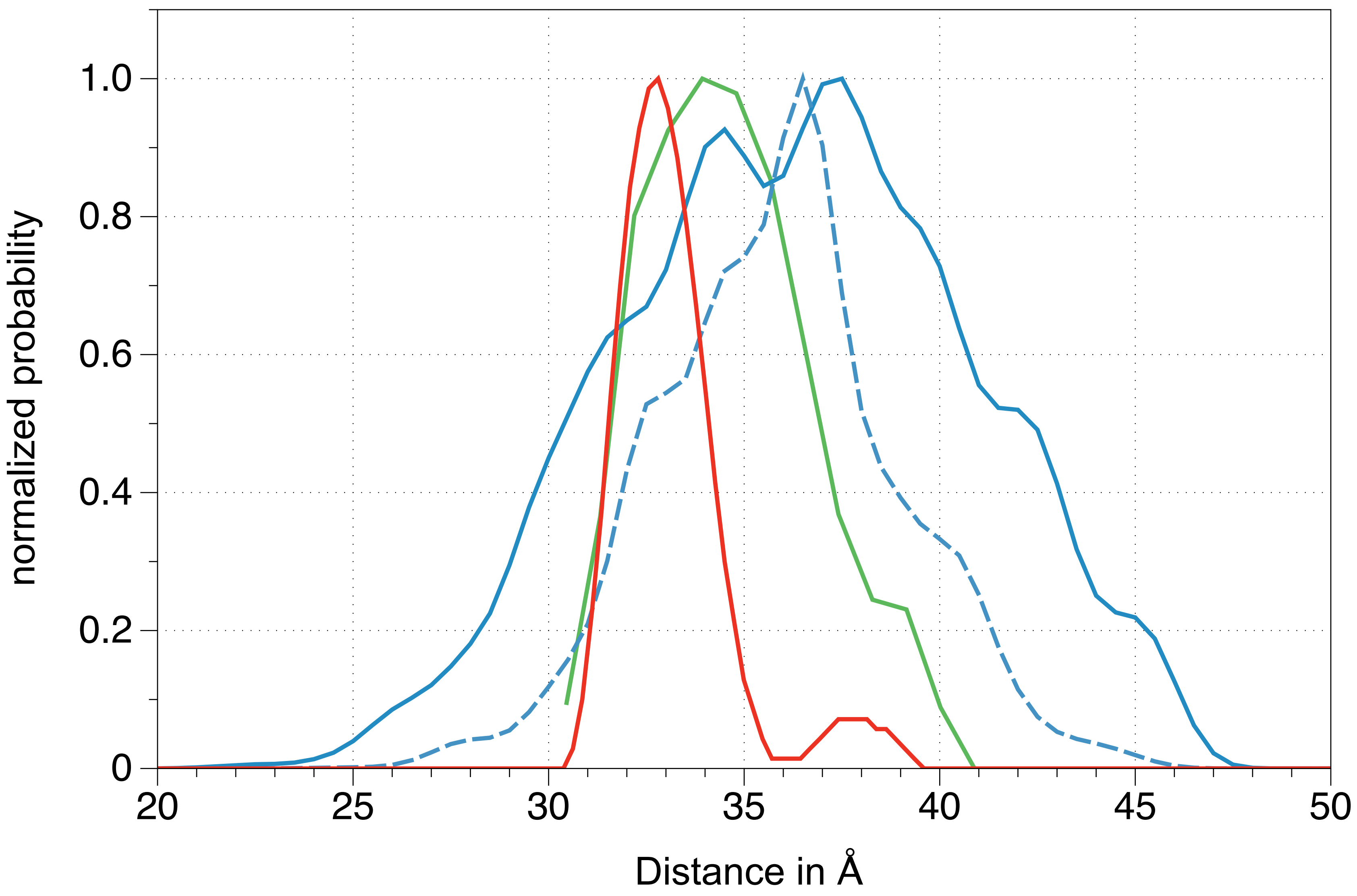

1.0
0.8
0.6
0.4
0.2
0
normalized probability
20
25
30
35
40
45
50
Distance in Å

H4L49

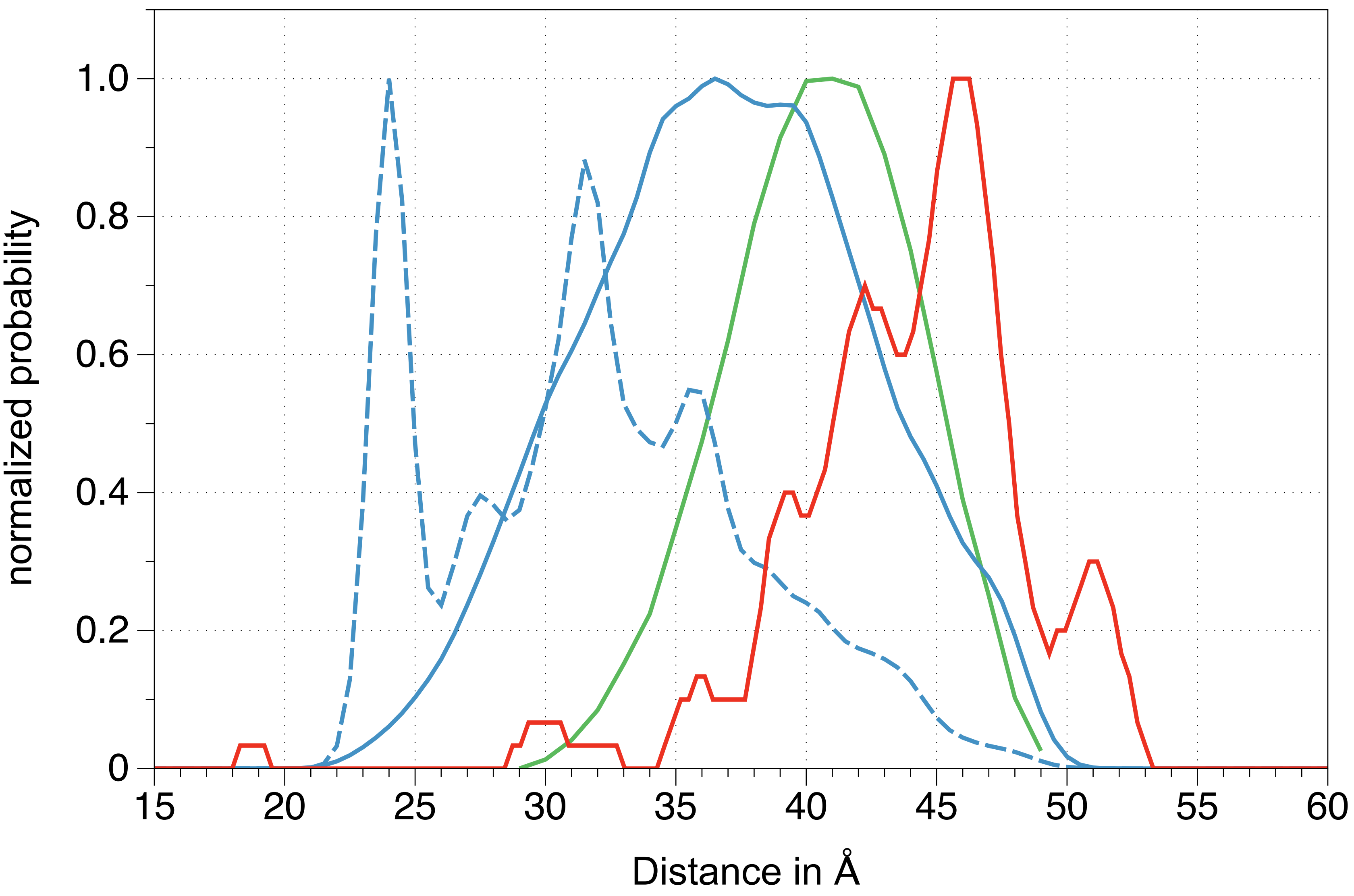

normalized probability
1.0
0.8
0.6
0.4
0.2
0
15
20
25
30
35
40
45
50
55
60
Distance in Å

H4V60

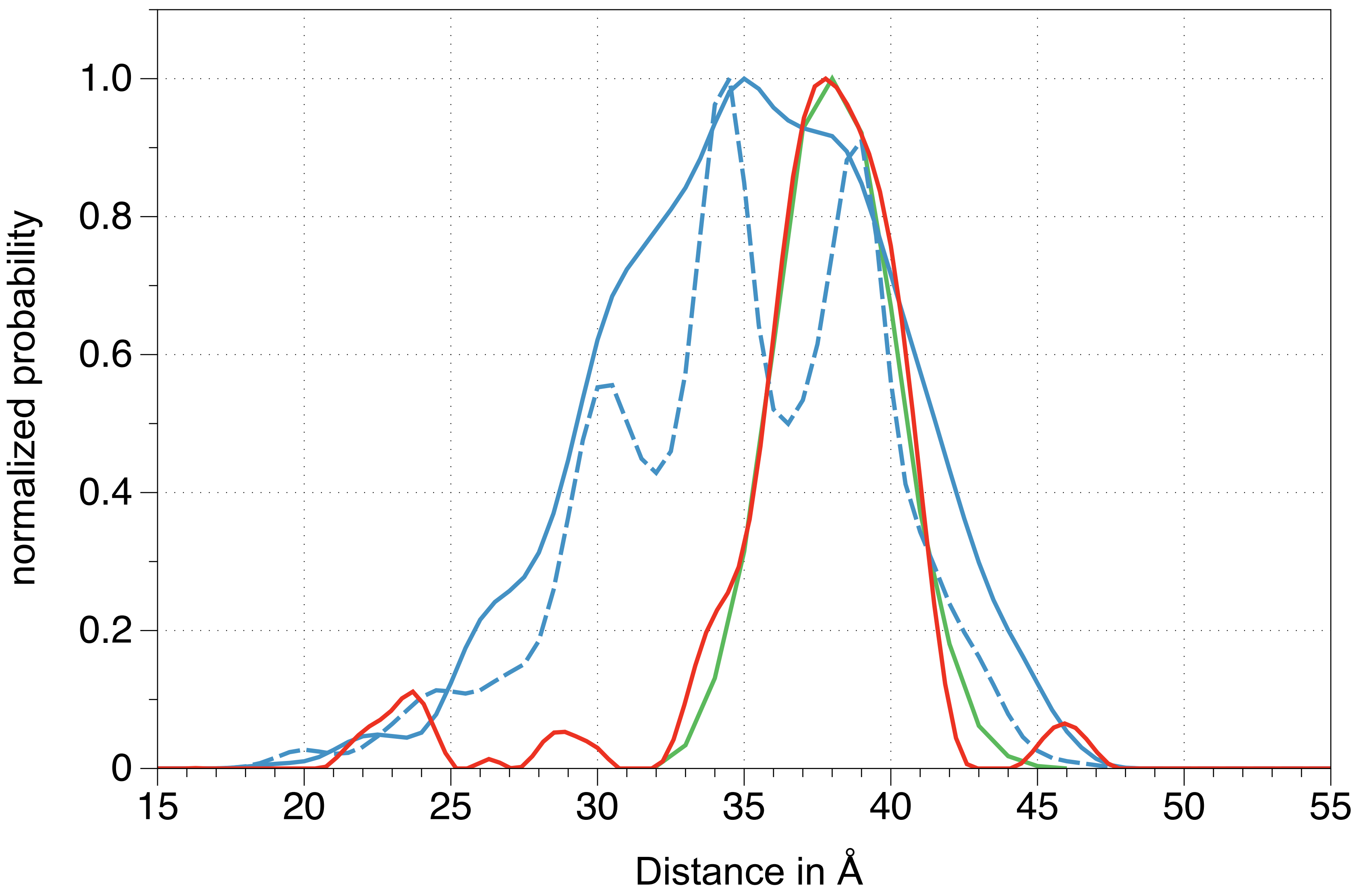

normalized probability
1.0
0.8
0.6
0.4
0.2
0
15
20
25
30
35
40
45
50
55
Distance in Å

H4E63

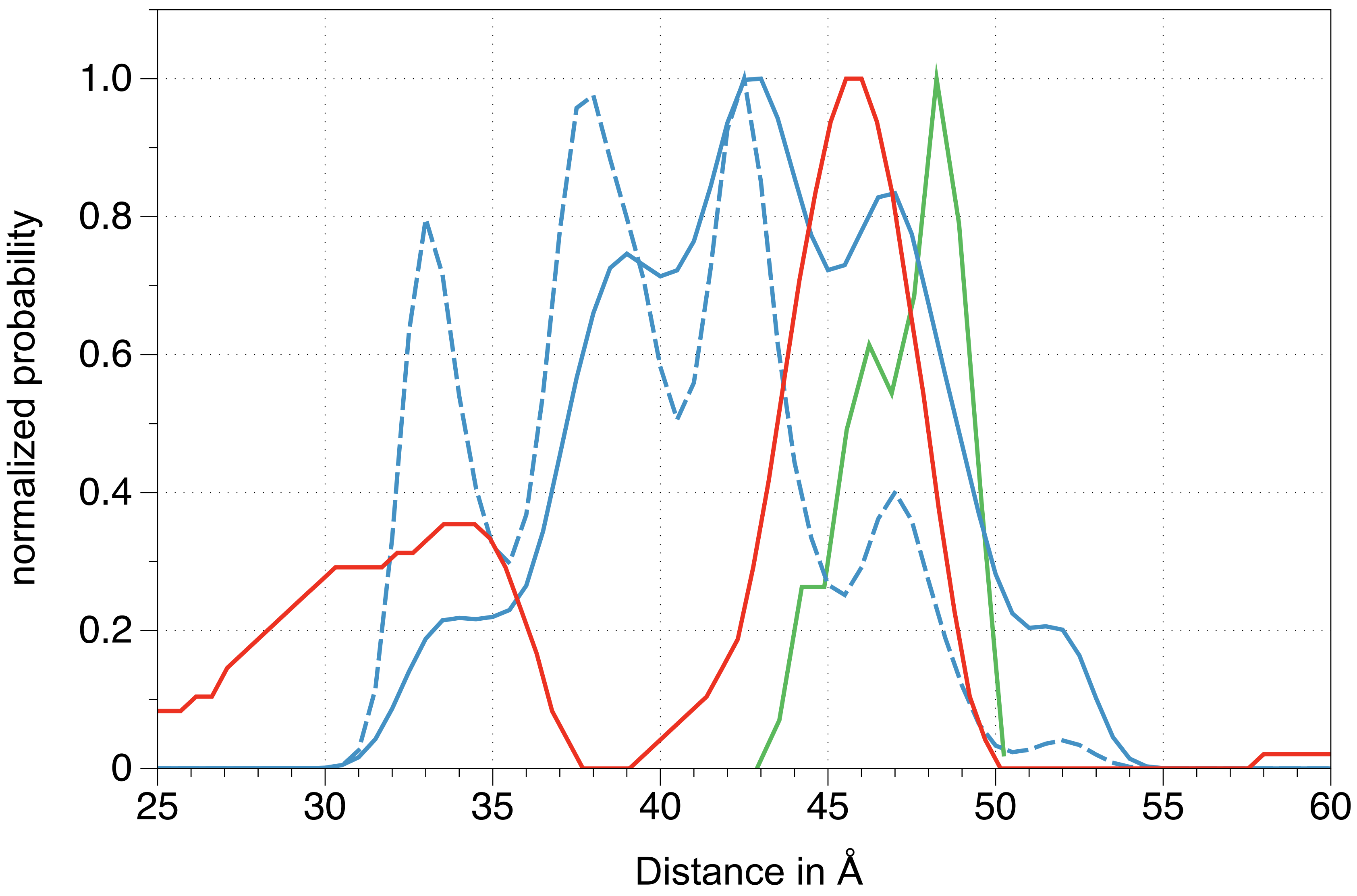

normalized probability
1.0
0.8
0.6
0.4
0.2
0
25
30
35
40
45
50
55
60
Distance in Å

H4R67

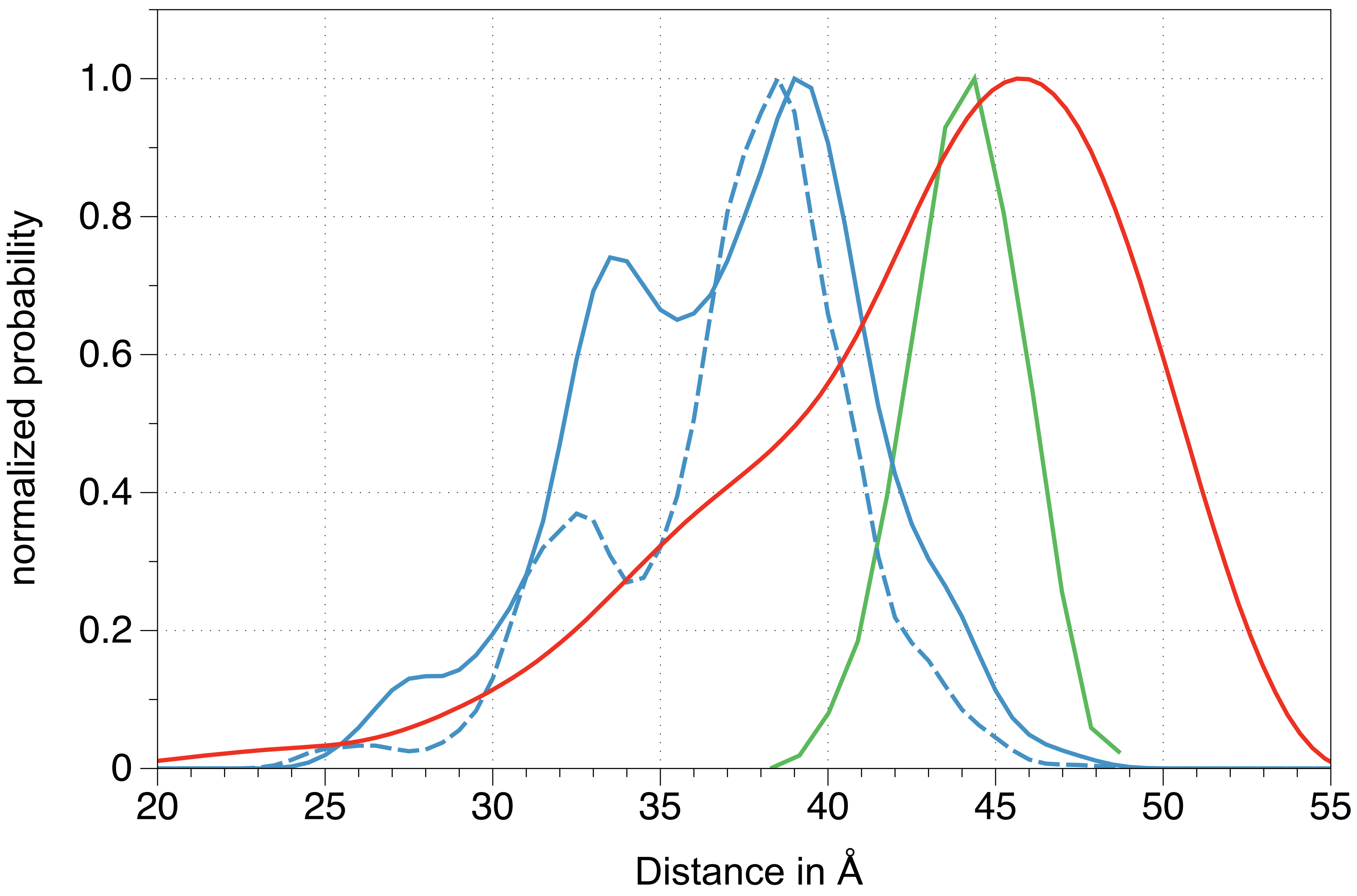

1.0
0.8
0.6
0.4
0.2
0
normalized probability
20
25
30
35
40
45
50
55
Distance in Å

H4T71

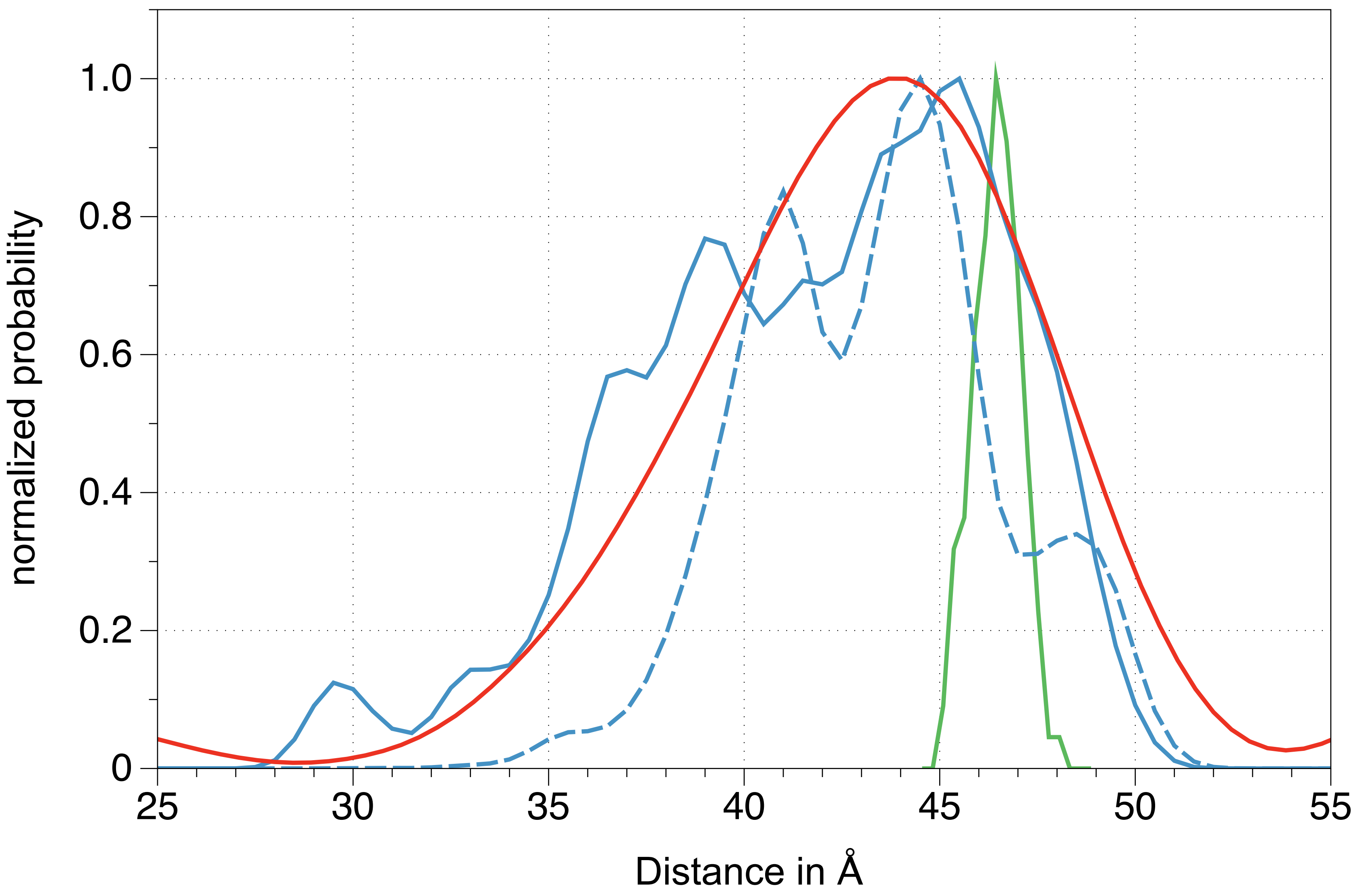

normalized probability
1.0
0.8
0.6
0.4
0.2
0
25
30
35
40
45
50
55
Distance in Å

H4T82

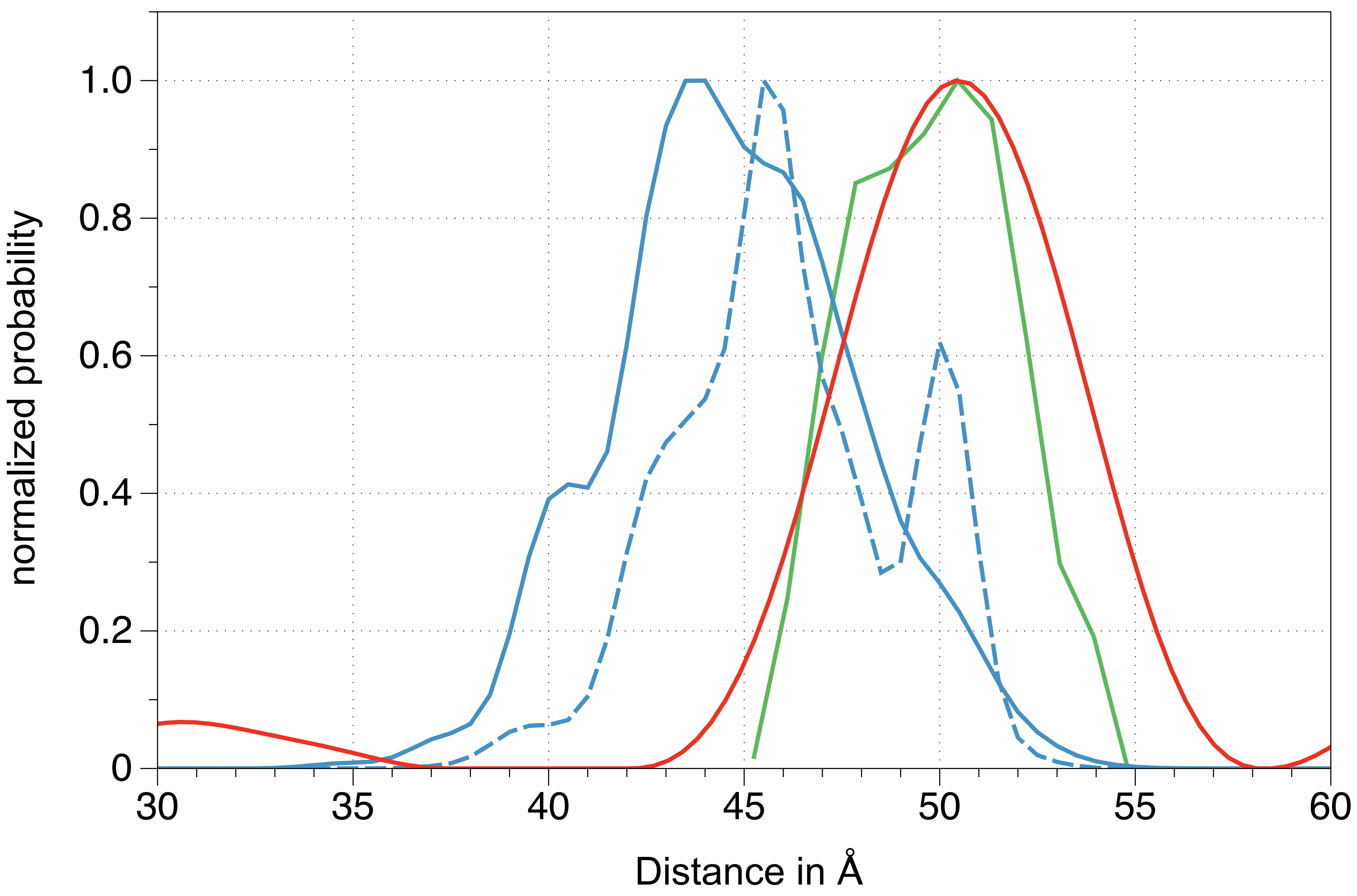

normalized probability
1.0
0.8
0.6
0.4
0.2
0
30
35
40
45
50
55
60
Distance in Å
