# Supplementary material for: MtsslWizard: In Silico Spin-Labeling and Generation of Distance Distributions in PyMOL
Source: Appl Magn Reson. 2012 Feb 3;42(3):377–91. doi: 10.1007/s00723-012-0314-0 (PMC3296949; doi:10.1007/s00723-012-0314-0)
Supplement: Supplementary file 2 — (PDF 380 kb) [file 723_2012_314_MOESM2_ESM.pdf]

Supplementary Figure 2

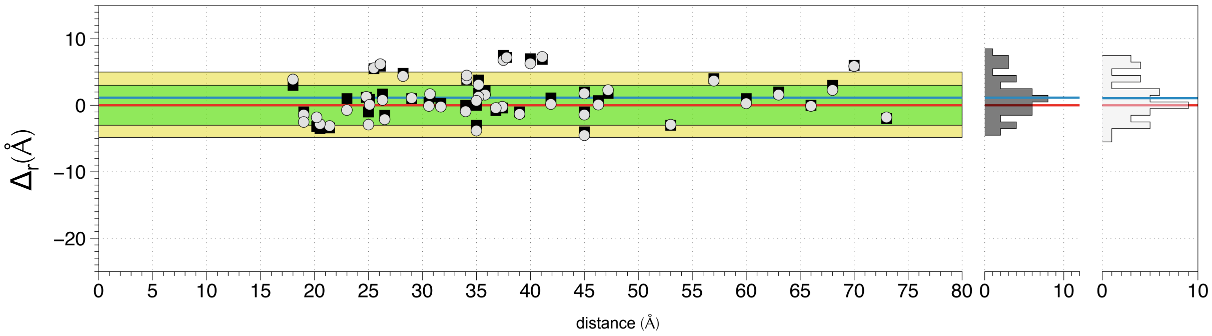

**mtsslWizard "vdW cutoff"**	**<Δr>** (Å)	**σ(Δr)** (Å)
3.4 Å	1.2	3.0
3.0 Å	1.1	3.1

Suppl. Fig. 2: Comparison of the analysis of the T4L and histone datasets with mtsslWizard using a 3.4 Å vs a 3.0 Å vdW cutoff. The x-axis shows the experimental distance and the y-axis the difference of the experimental value to the prediction (Δ_r). The ideal y=0 line is marked in red, the areas corresponding to different prediction errors are shaded with different colours (green: ≤ 3 Å, yellow: ≤ 5 Å, white: > 5 Å). The graph contains two plots, black squares show the analysis with a cutoff of 3.4 Å and grey circles with a cutoff of 3.0 Å. The two vertical histograms (dark grey: 3.4 Å; light grey: 3.0 Å) on the right visualize the spread of the residuals for both plots. The average difference ($<\Delta_r>$) between prediction and experiment and the standard deviation of this value ($\sigma(\Delta_r)$) are given in the table to the right. The average difference ($<\Delta_r>$) is also marked by blue horizontal lines in the histograms. The blue line in the residual plots (left) corresponds to the average difference with a cutoff of 3.4 Å. For this analysis the average distance of the prediction was compared to the average distance from the experimental data.
